# Regulation of cholesterol homeostasis in health and diseases: from mechanisms to targeted therapeutics

**DOI:** 10.1038/s41392-022-01125-5

**Published:** 2022-08-02

**Authors:** Yajun Duan, Ke Gong, Suowen Xu, Feng Zhang, Xianshe Meng, Jihong Han

**Affiliations:** 1 Department of Cardiology, The First Affiliated Hospital of USTC, Division of Life Sciences and Medicine, University of Science and Technology of China, Hefei, China; 2 Key Laboratory of Metabolism and Regulation for Major Diseases of Anhui Higher Education Institutes, College of Food and Biological Engineering, Hefei University of Technology, Hefei, China; 3 College of Life Sciences, Key Laboratory of Bioactive Materials of Ministry of Education, State Key Laboratory of Medicinal Chemical Biology, Nankai University, Tianjin, China

**Keywords:** Molecular medicine, Cardiovascular diseases

## Abstract

Disturbed cholesterol homeostasis plays critical roles in the development of multiple diseases, such as cardiovascular diseases (CVD), neurodegenerative diseases and cancers, particularly the CVD in which the accumulation of lipids (mainly the cholesteryl esters) within macrophage/foam cells underneath the endothelial layer drives the formation of atherosclerotic lesions eventually. More and more studies have shown that lowering cholesterol level, especially low-density lipoprotein cholesterol level, protects cardiovascular system and prevents cardiovascular events effectively. Maintaining cholesterol homeostasis is determined by cholesterol biosynthesis, uptake, efflux, transport, storage, utilization, and/or excretion. All the processes should be precisely controlled by the multiple regulatory pathways. Based on the regulation of cholesterol homeostasis, many interventions have been developed to lower cholesterol by inhibiting cholesterol biosynthesis and uptake or enhancing cholesterol utilization and excretion. Herein, we summarize the historical review and research events, the current understandings of the molecular pathways playing key roles in regulating cholesterol homeostasis, and the cholesterol-lowering interventions in clinics or in preclinical studies as well as new cholesterol-lowering targets and their clinical advances. More importantly, we review and discuss the benefits of those interventions for the treatment of multiple diseases including atherosclerotic cardiovascular diseases, obesity, diabetes, nonalcoholic fatty liver disease, cancer, neurodegenerative diseases, osteoporosis and virus infection.

## Introduction

Cholesterol is a waxy and fat-like substance with pivotal pathophysiological relevance in humans. More than two centuries ago, Michel Eugène Chevreul, a French chemist, found that cholesterol is one of the components in human gallstones.^1^ Following this event, many scientists input a lot efforts to elucidate cholesterol structure. In 1927, Heinrich Otto Wieland from Germany won the Nobel Prize in Chemistry for his work on clarifying the structure of cholesterol and bile acids. A year later, Adolf Windaus also from Germany was awarded the Nobel Prize in Chemistry for his work on sterols and the related vitamins, such as vitamin D which is derived from cholesterol.^2^ However, it was until 1932, the correct cholesterol structure was finally formulated.^1^

Cholesterol can be synthesized in our body and the biosynthesis of this complex molecule starts from acetyl coenzyme A (acetyl-CoA) with involvement of nearly 30 enzymatic reactions. Among these reactions, the step for reduction of 3-hydroxy-3-methylglutaryl coenzyme A (HMG-CoA) to mevalonate catalyzed by HMG-CoA reductase (HMGCR) is rate-limiting, indicating regulation of HMGCR expression/activity is critical for cholesterol biosynthesis. In 1964, Konrad Emil Bloch and Feodor Lynen won the Nobel Prize in the Medicine and Physiology for discovering the major intermediate reactions in the pathway for cholesterol biosynthesis.^3^

The cholesterol biosynthesis is an intensely regulated process biologically.^4^ The first demonstration of feedback inhibitory loop by the end product in biosynthetic pathways is that cholesterol inhibits its own synthesis intracellularly. In 1933, Rudolph Schoenheimer demonstrated that animals can also synthesize cholesterol, more importantly, he observed that the cholesterol synthesis in animal body was inhibited by cholesterol supplied in the diet. This finding laid the groundwork for discovering sterol regulatory element binding protein (SREBP) pathway.^5^ SREBP binds to the sterol regulatory element (SRE) in the proximal region of the promoter of *HMGCR*. The binding of SREBP triggers transcription of *HMGCR* to speed up cholesterol biosynthesis.^6^ SREBP is also able to bind to the SRE in the promoter of low-density lipoprotein receptor (*LDLR*), the molecule responsible for the LDL cholesterol (LDL-C) clearance in the liver.^6^ As a transcription factor, SREBP needs to be chaperoned by SREBP cleavage activating protein (SCAP) from endoplasmic reticulum (ER) to Golgi, where SREBP is cleaved into mature and functional form by sphingosine-1-phosphate (S1P) and S2P proteases. Cholesterol can interact with unmatured SREBP on the ER.^6,7^ Thus, when the cellular cholesterol level is reduced, the mature SREBP is increased and consequently to activate HMGCR expression. Reciprocally, increased cellular cholesterol level inhibits HMGCR expression.^8^

Mounting evidence has established the intricate link between cholesterol levels and atherosclerotic cardiovascular disease (ASCVD). In fact, atherosclerosis is a disease with a long research history. The role of cholesterol in atherosclerosis was initially reported in 1910.^9^ Adolf Windaus found that cholesterol content in atherosclerotic plaques of human diseased aorta was 25 times higher than that of normal aortas.^8^ Three years later, the first experimental recapitulation of atherosclerosis was completed by Nikolaj Anitschkow. He fed rabbits pure cholesterol contained in diet, and observed severe atherosclerosis in aortas of the animals.^10^ In history, Robert Wissler and coworkers set up the first mouse model for atherosclerosis in 1960s.^11^ Now, the mice with genetic manipulation, such as *ApoE* or *LDLR* deficient mice, is the most frequently-used animal model for investigation on atherosclerosis based on the time and cost issues.

Accumulation of cholesterol in atherosclerotic plaques may lead to formation of cholesterol crystals, a hallmark of advanced atherosclerotic plaques.^12–14^ Cholesterol crystals can stimulate the generation of NOD-, LRR- and pyrin domain-containing protein 3 (NLRP3) inflammasome to promote inflammation and accelerate atherogenesis.^15,16^ It also induces arterial inflammation and involves in destabilizing atherosclerotic plaques.^17^ Currently, the critical role of inflammation in mediating all stages of atherosclerosis has been well defined, and targeting inflammatory pathways may provide a new notion for atherosclerosis prevention and/or treatment.^18,19^

Cholesterol is a hydrophobic molecule which travels through the bloodstream on proteins called “lipoproteins”. Ultracentrifuge was used to separate lipoproteins in plasma by John Gofman. He also demonstrated that heart attacks were associated with increased blood cholesterol levels, especially LDL-C. In contrast, when blood high-density lipoprotein (HDL) levels rise, the heart attack frequency was reduced.^20–22^ Moreover, the beneficial effects of HDL cholesterol (HDL-C) and the negative effects of LDL-C on heart diseases were further confirmed by the Framingham Heart Study, one of the most important epidemiological studies in cardiovascular arena.^23^

It was first time that Carl Müller discovered the genetic link between cholesterol and heart attacks. He demonstrated that families with high plasma cholesterol levels and early-onset heart disease are autosomal dominant traits.^24^ This kind of disease is called familial hypercholesterolemia (FH). Avedis Khachadurian described two different clinical forms of FH in inbred families. Homozygous patients showed severe hypercholesterolemia at birth (the plasma cholesterol level in this kind of patients is about 800 mg/dl), and they can have heart attack as early as 5 years old, while the heterozygous patients showed cholesterol levels of 300–400 mg/dl and early-onset heart attack usually between 35-60 years old.^25^ In 1970s, Joseph Goldstein and Michael Brown discovered the essence of LDLR functional defect in FH, which led them to be awarded the Nobel Prize in 1985.^26^ The cellular uptake of LDL requires LDLR and most LDL-C is cleared from circulation by LDLR expressed in the liver. In the absence of LDLR, LDL-C reaches high level in the circulation, eventually deposits in the artery to drive the formation of atherosclerotic plaques.^27^ The seminal work by Goldstein and Brown strongly supports the importance of lipid hypothesis in onset of cardiovascular diseases (CVD). In addition to HMGCR, SREBP also regulates LDLR expression in response to cellular cholesterol levels to fine-tune the cholesterol level in cell membranes constant.^6–8^

Based on the evidence from epidemiological studies and randomized clinical trials, a cholesterol hypothesis was suggested which indicates the high circulating cholesterol level as a major risk factor for ASCVD while cholesterol-lowering strategies can reduce ASCVD risk.^28^ In 1976, Akira Endo discovered the first HMGCR inhibitor, thus inaugurating a category of cholesterol-lowering drugs called statins, which is a therapeutic milestone for CVD treatment.^29^ Statins deprive hepatocytes of endogenous synthesis as a source of cholesterol, which can alleviate the feedback inhibition of LDLR, and thus the increased LDLR expression will further reduce plasma LDL-C levels.^30^ In 1987, lovastatin (Mevacor) developed by Merck was approved as the first statin for human use to lower plasma LDL-C. Currently, statins are used as the first-line therapy to reduce LDL-C and prevent ASCVD.^31^

However, the doubled dose of a statin only leads to about 6% increase in LDL-C lowering efficacy, which may cause statin resistance/intolerance.^32^ Thus, there is a need to develop novel lipid-lowering approaches beyond statins. In 2002, ezetimibe was introduced as an intestinal cholesterol absorption inhibitor to decrease total cholesterol (TC) and LDL-C levels. In 2003, Nabil Seidah and co-workers discovered proprotein convertase subtilisin/kexin type 9 (PCSK9).^33^ PCSK9 is synthesized in the liver and then secreted into plasma. The circulating PCSK9 can bind hepatic LDLR and disrupt the recycle in which LDLR returns to the cell surface after internalization and release of the bound LDL-C.^34,35^ The decrease of cell surface LDLR results in impaired LDL-C clearance and elevated LDL-C level. In 2015, alirocumab and evolocumab, the fully human anti-PCSK9 antibodies, were approved by US FDA to treat patients with hypercholesterolemia.^36^ Likewise, a long-acting synthetic siRNA targeting *PCSK9* mRNA called inclisiran was developed by Novartis and used to treat hypercholesterolemia. In 2020, inclisiran was approved by EU.^37^ ATP citrate lyase (ACLY) is a cytoplasmic enzyme catalyzing acetyl-CoA generation, with which cholesterol biosynthesis begins.^38^ Thus, inhibition of ACLY can also reduce cholesterol synthesis. Indeed, among ACLY inhibitors, bempedoic acid was approved by US FDA in 2020 for hypercholesterolemia treatment.^39^ Notably, bempedoic acid only acts locally in the liver, thereby avoiding the muscle-related toxicities associated with statin use.^40^

Taken together, when reviewing the milestones of cholesterol research, we realize that the findings in regulation of cholesterol homeostasis determined the progress on the development of therapeutic strategies, and the feedback from clinical observations may further advance the investigation on cholesterol homeostasis, thereby promoting clinical progress. “HMGCR-statin-LDLR-rule of 6%-PCSK9” should be a typical example. To lower cholesterol synthesis in the liver, statins were initially developed to inhibit HMGCR. Later on, Brown and Goldstein proved that statins increased LDLR on hepatocyte surfaces to soak up excess blood LDL-C, thereby reducing heart attack. Associated with wide use of statins in clinics, the “rule of 6%” was observed, which was mysterious until the discovery of PCSK9. SREBP-2 activates LDLR and PCSK9 expression simultaneously and activated PCSK9 binds to LDLR toward lysosomal degradation, which clearly antagonizes the efficacy of statin-induced LDL-C clearance. Therefore, PCSK9 has become a valuable therapeutic target for cholesterol-lowering therapy and PCSK9 inhibitors have been developed rapidly.

Nowadays, the cholesterol homeostasis is involved in development of various diseases and determined by processes of biosynthesis, uptake, efflux, transport, storage, utilization, and/or excretion. Therefore, in this article, we will summarize the key regulations in cholesterol homeostasis and cholesterol-lowering interventions. Furthermore, we will discuss the benefits of the pharmaceutical interventions targeting cholesterol homeostasis on the multiple related diseases, such as ASCVD, obesity, diabetes and more.

## Methods

The references used in this review were acquired using the PubMed search engine with a time range from January 1930 to April 2022 by four researchers (Y. D., K. G., F. Z. and X. M.) independently. A list of relevant literature that met the inclusion criteria was manually searched. The following search strategy was applied by using the keywords of “cholesterol history”, “cholesterol development”, “cholesterol metabolism”, “cholesterol homeostasis”, “cholesterol synthesis”, “cholesterol transport”, “ASCVD cholesterol”, “ASCVD cholesterol ester”, “ASCVD foam cells”, “ASCVD statins”, “ASCVD ezetimibe”, “ASCVD PCSK9 inhibitor”, “ASCVD bempedoic acid”, “ASCVD bile acid sequestrants”, “ASCVD lomitapide”, “ASCVD evinacumab”, “ASCVD fibrates”, “ASCVD lipoprotein apheresis”, “ASCVD APOC3”, “ASCVD lipoprotein (a)”, “ASCVD LXRs”, “ASCVD LOX-1”, “ASCVD SR-BI”, “ASCVD LCAT”, “ASCVD MiR-33”, “ASCVD MiR-122”, “ASCVD prekallikrein”, “cholesterol homeostasis NAFLD”, “cholesterol homeostasis obesity”, “cholesterol homeostasis diabetes”, “cholesterol homeostasis Alzheimer’s disease”, “cholesterol homeostasis Parkinson’s disease”, “cholesterol homeostasis Huntington’s disease”, “cholesterol homeostasis cancer”, “cholesterol homeostasis osteoporosis”, or “cholesterol virus infection”. No additional restrictions were placed on the type of research model (in vivo*/*in vitro), article type (e.g., research article, review, editorial, letter, etc.), or publication language. References cited in articles associated with the literature search were also analyzed for additional information. The studies were excluded from the content retrieved if they are irrelevant or of limited relevance to the main topic.

## Regulatory mechanisms of cholesterol homeostasis

Disturbed cholesterol homeostasis is not only the pathological basis of cardiovascular and cerebrovascular diseases, but also participates in the progression of other kinds of diseases including neurodegenerative diseases and cancers. Maintaining cholesterol homeostasis plays a crucial role physiologically. Normally, the cholesterol homeostasis can be well maintained by a dynamic balance among the intake, biosynthesis, transport, cellular uptake and efflux, and/or esterification. Thus, we will review the state-of-the-art research on the molecular mechanisms that regulate cholesterol homeostasis, and provide future research directions.

### Sources of cholesterol: intake or biosynthesis

#### Dietary cholesterol

Two main sources of cholesterol are present in our body, one is through dietary intake, known as exogenous cholesterol or dietary cholesterol; and another one is through the de novo biosynthesis, known as endogenous cholesterol.^41^ A variety of daily foods, such as eggs, animal offal and seafood, contain cholesterol, of which eggs are the main source of dietary cholesterol.^42^ The solubility of cholesterol in an aqueous environment is extremely low, so before absorption, it must be dissolved into bile salt micelles, which can be transported to the brush edge of intestinal cells. Then the net cholesterol is absorbed, the process is regulated by Niemann-Pick C1 (NPC1) like 1 (NPC1L1) protein. Inhibition of NPC1L1 by ezetimibe can reduce cholesterol absorption, thereby improving coronary artery disease.^43^ After a series of processes, the absorbed cholesterol is esterified and then secreted into circulation as chylomicrons and eventually being taken up by the liver.^44,45^ In addition, phytosterols/phytostanols can be added into the foods to replace cholesterol in micelles, leading to less cholesterol is absorbed by enterocytes and enters the liver.^46^

To maintain hepatic cholesterol pool, the liver enhances LDL-C uptake from plasma by increasing LDLR expression and decreases cholesterol efflux, thereby reducing plasma TC and LDL-C levels.^47^ NPC1L1 promoter also contains a SRE, the sterol-sensing structural domain, therefore, NPC1L1 expression is repressed by a high-cholesterol contained diet and increased by cholesterol-depleted food.^48^ In addition, endogenous cholesterol synthesis is negatively regulated by the exogenous cholesterol. Hepatic cholesterol biosynthesis accounts for approximately three-quarters of the total endogenous cholesterol production at the low cholesterol intake situation. However, hepatic cholesterol biosynthesis is completely inhibited when 800–1000 mg exogenous cholesterol is ingested in experiments with baboons and humans.^49,50^

#### Biosynthesis of cholesterol

**Fig. 1:**
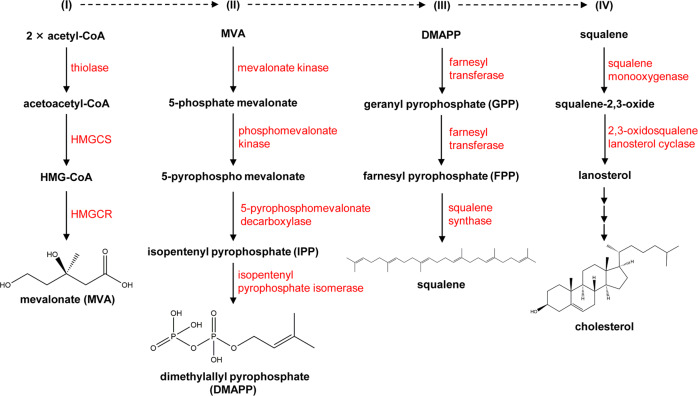
The pathway for cholesterol biosynthesis. In cholesterol biosynthesis, all the carbon atoms are derived from acetyl-CoA. The biosynthesis of cholesterol can be divided into four stages. (I) Synthesis of mevalonate (MVA). Two molecules of acetyl-CoA are reversely catalyzed by thiolase to form acetoacetyl-CoA. Acetoacetyl-CoA and acetyl-CoA are catalyzed to form 3-hydroxy-3-methylglutaryl coenzyme A (HMG-CoA) by HMG-CoA synthase (HMGCS). Finally, the HMG-CoA is catalyzed by HMG-CoA reductase (HMGCR) to convert to MVA, a step that requires two molecules of NADPH and H^+^ and determines the rate of cholesterol biosynthesis. (II) Production of isopentenyl pyrophosphate (IPP) and dimethylallyl pyrophosphate (DMAPP). MVA is sequentially phosphorylated twice by mevalonate kinase and phosphomevalonate kinase to produce 5-pyrophosphate mevalonate, which is further decarboxylated by 5-pyrophosphatemevalonate decarboxylase to produce isopentenyl pyrophosphate (IPP). IPP is converted to dimethylallyl pyrophosphate (DMAPP) catalyzed by isopentanoyl pyrophosphate isomerase, and DMAPP is used together with IPP as the starting materials for the third step of cholesterol synthesis. (III) Synthesis of squalene. IPP and DMAPP are condensed by farnesyl transferase to form geranyl pyrophosphate (GPP), followed by a second condensation reaction between GPP and IPP to form farnesyl pyrophosphate (FPP), and finally two molecules of FPP are condensed by squalene synthase to form squalene. (IV) Squalene cyclizes to form lanosterol and subsequently to synthesize cholesterol. Squalene forms a closed loop catalyzed by squalene monooxygenase and 2,3-oxidosqualene lanosterol cyclase to form lanosterol. Lanosterol is converted into cholesterol in more than twenty steps totally

Cholesterol can be synthesized by all nucleated cells, with most by hepatocytes, indicating the liver is the main site for cholesterol biosynthesis in vivo.^51^ Acetyl-CoA is used as the starting material for cholesterol biosynthesis *via* the mevalonate pathway including nearly 30 enzymatic steps (Fig. 1). The biosynthesis of cholesterol can be divided into four stages: (I) Synthesis of mevalonate (MVA); (II) Production of isopentenyl pyrophosphate (IPP) and dimethylallyl pyrophosphate (DMAPP); (III) Synthesis of squalene; (IV) Squalene cyclizes to form lanosterol and subsequently to synthesize cholesterol. The process is regulated by a negative feedback mechanism with the downstream products.^52,53^ The SREBP pathway and the HMGCR degradation pathway serve as two major negative feedback regulatory mechanisms to regulate cholesterol de novo synthesis.^54^

SREBPs, the transcription factors anchored to the ER, include three isoforms, SREBP1a, SREBP1c and SREBP2. The N-terminal sequences of SREBPs belong to the basic-helix-loop-helix-leucine zipper (bHLH-Zip) protein superfamily.^6,55^ When cellular cholesterol is depleted, the N-terminus of SREBPs can be cleaved into the form of mature and functional SREBP, which can translocate with chaperone by SCAP to the nucleus where the mature SREBP identifies and binds to the SRE in the target gene promoter, followed by activation of these genes transcription.

**Fig. 2:**
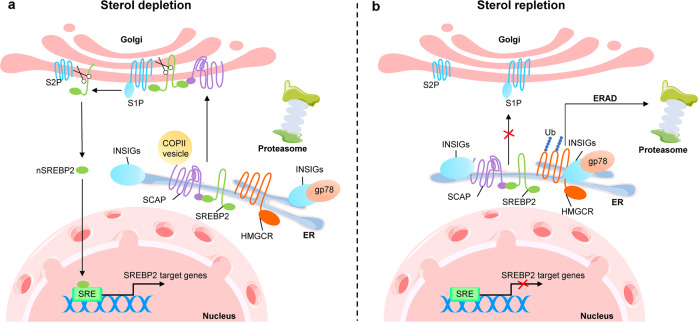
SREBP2 pathway in regulation of cholesterol biosynthesis. The process of cholesterol biosynthesis is strictly regulated by negative feedback, of which the sterol regulatory element binding protein (SREBP) pathway and the HMG-CoA reductase (HMGCR) degradation pathway are the two main mechanisms of negative feedback regulation. **a** SREBP2 forms a complex with SREBP cleavage activating protein (SCAP) at the ER. When sterol depletion occurs to cells, SCAP binds to COPII vesicles, allowing the SCAP-SREBP complex to translocate from the ER to the Golgi for cleavage. SREBP2 is sequentially cleaved by S1P and S2P in the Golgi, and the N-terminal of SREBP2 is subsequently transported to the nucleus, where the N-terminal of SREBP2 recognizes and binds to the SRE sequence on the target gene promoter to activate the target gene transcription. In addition, HMGCR is also prevented from binding to INSIGs and gp78 (ubiquitin ligase) during cholesterol depletion, thereby stabilizing HMGCR to activate cholesterol biosynthesis. **b** When the cell sterol is replete, it triggers the interaction of SCAP with INSIGs, resulting in blocking the binding of SCAP to COPII and keeping the SCAP-SREBP2 complex in the ER. At the same time, HMGCR also binds to INSIGs and gp78, which catalyzes the ubiquitination of HMGCR. The ubiquitinated HMGCR is eventually degraded in the proteasome via ER-related degradation (ERAD). Ub ubiquitin

Further studies revealed that SREBPs interact with SCAP to form a complex in a stoichiometric ratio of 4:4.^56^ When ER membrane cholesterol is depleted, SCAP binds to COPII vesicles that allows the SCAP-SREBP complex to move from ER to Golgi for cleavage. When ER membrane cholesterol exceeds 5% of total ER lipids at molar basis, cholesterol and oxysterols, such as 25-hydroxycholesterol, trigger the interaction between SCAP sterol-sensing domain (SSD) and insulin-induced gene (INSIG), thereby blocking the binding of SCAP to COPII vesicles and keeping the SCAP-SREBP complex in the ER^57,58^ (Fig. 2). At present, the structure of SCAP in cholesterol-free and cholesterol-bound states, as well as the structure of SCAP-INSIG or SCAP-COPII complex need to be verified by further ultrastructural study. In the recent studies, the conformation of SCAP-INSIG has been resolved by the cryo-electron microscopy technology.^59,60^ These findings may benefit to the screening of the small molecules affecting the conformation change of SCAP to inhibit cholesterol synthesis.f

In the process of cholesterol biosynthesis, HMGCR is subjected to strict feedback regulation^54^ (Fig. 2). As a target gene of SREBP2, HMGCR is regulated by SREBP2 at the transcriptional level. In addition to this long-term transcriptional regulation, HMGCR is also subject to short-term epigenetic modulation. Ubiquitination and phosphorylation of HMGCR are two common post-translational modifications.^61^

HMGCR is located in the ER and divided into an N-terminal transmembrane region and a C-terminal cytoplasmic region based on its function and structure. The amino acid sequence of the transmembrane region is highly conserved and the membrane structural domain can respond to increases of sterols and mediate its own degradation.^62^ In 2005, Song et al. found that gp78, also known as autocrine motility factor receptor (AMFR), functions as a ubiquitin ligase to mediate HMGCR degradation. In cells with high cholesterol levels, INSIG binds to both HMGCR and gp78, which allows gp78 to catalyze the ubiquitination of the lysine residues at position 89 and 248 of HMGCR.^63^ The ubiquitin fusion degradation 1 (Ufd1) protein contains ubiquitin binding sites, which serves as an accelerator of degradation by binding to gp78 to accelerate HMGCR degradation.^64^ Meanwhile, gp78 is also involved in the ubiquitination and proteasomal degradation of INSIGs, and promotes SREBP maturation and lipid synthesis. Surprisingly, in hepatic gp78-deficient mice, both cholesterol and fatty acid synthesis were reduced despite enhanced HMGCR enzymatic activity, which resulted from reduced SREBP maturation to suppress downstream gene expression.^65,66^ The recent studies have found that increased postprandial insulin and glucose concentrations enhance the effect of mechanistic target of rapamycin complex 1 (mTORC1) on phosphorylation of ubiquitin specific peptidase 20 (USP20). Once phosphorylated, USP20 can be recruited to HMGCR complex to antagonize HMGCR degradation. Thus, deleting or inhibiting USP20 significantly reduces diet-induced weight gain, serum and liver lipid levels, improves insulin sensitivity and increases energy expenditure.^67^ Taken together, these studies suggest that ubiquitin ligase gp78 and USP20 could be the new targets for treatment of diseases with cholesterol metabolic disorders.

In addition to ubiquitination, HMGCR is also regulated by phosphorylation. Clarke and Hardie found that Ser-872 within the catalytic fragment of rat HMGCR can be phosphorylated by AMP-activated protein kinase (AMPK), which inactivates HMGCR and reduces the flux of the formaldehyde valerate pathway.^68^ Meanwhile, Sato et al. found that AMPK-activated phosphorylation of Ser-872 did not affect sterol-mediated feedback regulation of HMGCR, but functioned when cellular ATP levels were depleted, thereby reducing the rate of cholesterol synthesis and preserving cellular energy stores.^69^ In contrast, dephosphorylation of HMGCR activates itself and increases cholesterol synthesis. Studies have shown that miR-34a, a microRNA increased in nonalcoholic fatty liver disease (NAFLD), dephosphorylates HMGCR *via* inactivating AMPK, leading to dysregulation of cholesterol metabolism and increased risk of cardiovascular disease.^70^ Subclinical hypothyroidism leads to elevated serum thyroid stimulating hormone (TSH) and elevated serum cholesterol levels. Zhang et al. found that TSH can reduce HMGCR phosphorylation to increase its activity in the liver *via* AMPK also, revealing a mechanism for hypercholesterolemia in subclinical hypothyroidism.^71^

### Uptake and transport of cholesterol

**Fig. 3:**
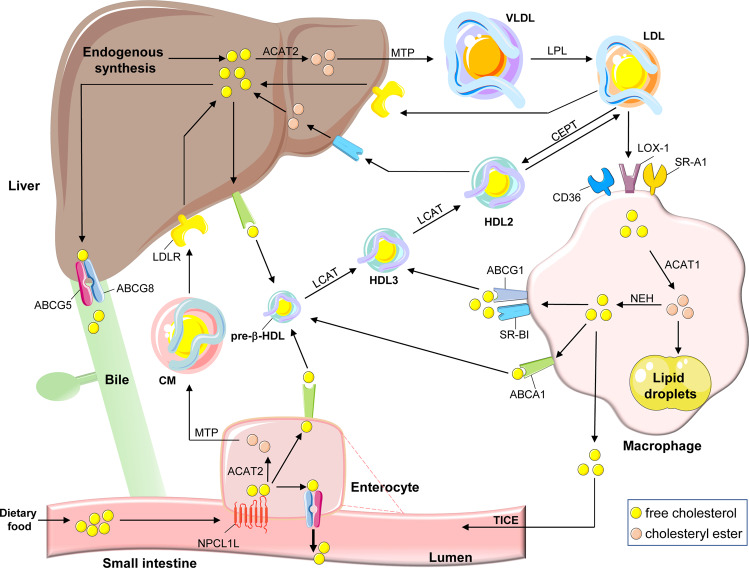
Regulation of cholesterol transport. Daily food and the hepatic endogenous synthesis are the two main sources of human cholesterol, of which dietary free cholesterol (FC) uptake is mediated by Niemann-Pick C1 Like 1 (NPC1L1) protein in enterocytes. The endocytosis of cholesterol by NPC1L1 responds to the change of cellular cholesterol concentration. FC taken up by NPC1L1 in enterocytes is esterified to cholesteryl ester (CE) by acyl-CoA:cholesterol acyltransferase 2 (ACAT2), which is loaded into ApoB-48 with triglycerides (TG) mediated by microsomal triglyceride transfer protein (MTP), to form chylomicron (CM). After TG in CM is hydrolyzed and utilized, most of the remaining cholesterol will be absorbed through low-density lipoprotein receptor (LDLR) in the liver. In contrast, some unesterified cholesterol is pumped back to the intestinal lumen by ATP-binding cassette (ABC) transport proteins G5 and G8 (ABCG5/ABCG8) or synthesized into pre-β-HDL by ABCA1 and released into circulation. Cholesterol synthesized endogenously in the liver is converted into VLDL with TG, ApoB-100, and most of VLDL is then converted into LDL, which is the main carrier for transporting endogenous cholesterol. LDL is taken up by scavenger receptors in macrophages, where expression of CD36, scavenger receptor A1 (SR-A1), and LDL receptor 1 (LOX1) is increased in atherosclerosis, further promoting cholesterol accumulation. LDL is endocytosed into macrophages and hydrolyzed by lipase (LAL) to produce FC. Excess FC is esterified by ACAT1 and stored as lipid droplets, and the excess accumulation of CE in macrophages can contribute to formation of foam cells. To mediate cholesterol efflux, macrophages hydrolyze CE into FC by the neutral cholesteryl ester hydrolase (NEH). Macrophage-mediated cholesterol efflux includes simple diffusion, SR-BI-facilitated diffusion, and ABCA1/ABCG1-mediated efflux. Among them, simple diffusion dominates cholesterol efflux in normal macrophages, regulated by cholesterol concentrations. In cholesterol overloaded macrophages, ABCA1 and ABCG1 are critical for cholesterol efflux. ABCA1 is able to bind to ApoA-I to mediate the production of pre-β-HDL, lecithin cholesterol acyltransferase (LCAT) further matures pre-β-HDL particles into HDL3, while ABCG1 and SR-BI mediate cholesterol flow directly to HDL3. HDL3 is further esterified by LCAT to produce HDL2, in which CE is eventually taken up by SR-BI in the liver and converted to FC. In addition, CE in HDL2 particles can be exchanged by cholesteryl ester transfer protein (CETP) to LDL particles, which are subsequently taken up by LDLR. Excess cholesterol in the liver is excreted into the bile mediated by ABCG5/ABCG8 and eventually enters the intestinal lumen for excretion in feces. Some other cholesterol in the blood can be excreted directly into the intestinal lumen via transintestinal cholesterol excretion (TICE) pathway in enterocytes

Dietary cholesterol absorbed by enterocytes or hepatic de novo synthesized cholesterol can form the protein-lipid complexes with lipoproteins and then release into circulation, followed by transportation to cells for utilization. In humans, about a quarter of excess cholesterol is excreted directly through enterocytes into feces, and the rest enters the liver via reverse cholesterol transport (RCT) and to be excreted with bile. Only a small percentage is re-circulated back into the free cholesterol (FC) pool^72–74^ (Fig. 3). A variety of proteins are involved in cholesterol uptake and transport. Thus, targeting these key proteins to regulate cholesterol levels is also a potential strategy for treatment of hypercholesterolemia and CVD.^75^

#### Cholesterol uptake and efflux in enterocytes

Dietary cholesterol is one of the main sources of cholesterol access in humans, and its uptake is mediated by NPC1L1 protein in enterocytes.^45^ NPC1L1 contains 13 transmembrane helices, five of which form the SSD that mediates NPC1L1 movement between the plasma membrane and the endocytic recycling compartment in response to intracellular cholesterol concentrations.^76,77^ In addition, the N-terminal structural domain of NCP1L1 has a sterol-binding pocket which interacts with cholesterol to change NPC1L1 conformation and allows cholesterol to enter cells.^78^ In earlier years, Song et al. found that the VNXXF (X for any amino acid) sequence at the C-terminus of NPC1L1 is involved in clathrin/adaptin 2-dependent endocytosis to mediate cholesterol uptake.^79,80^ However, NPC1L1-mediated cholesterol uptake is not mainly dependent on endocytosis.^81^

In 2020, the NPC1L1 structure was fully elucidated by the cryo-electron microscopy, making it easier to understand the mechanism of NPC1L1-mediated cholesterol uptake.^82^ After binding to the sterol-binding pocket, cholesterol triggers NPC1L1 conformation changes to form a delivery tunnel for cholesterol uptake by cells.^82^ Recently, Hu et al. found that SSD in NPC1L1 can respond to cholesterol concentrations by binding different amounts of cholesterol.^83^ In addition, the effective cholesterol uptake by NPC1L1 depends on its dimerization.^84^ Based on the crucial role of NPC1L1 in cholesterol uptake, ezetimibe has been developed and used clinically as an inhibitor of hypercholesterolemia, and other NPC1L1 inhibitors are being developed.^85,86^ Cellular cholesterol uptake by NPC1L1 is then esterified by acyl-CoA: cholesterol acyltransferase (ACAT) 2 in the ER and loaded with triglycerides (TG) into ApoB-48 to form chylomicrons. The mature chylomicrons are eventually transported into circulation, where TG is hydrolyzed for use in peripheral tissues and the majority of cholesterol is absorbed by the liver. In contrast, FC can be pumped back into intestinal lumen *via* ATP-binding cassette (ABC) transport protein G5 and G8 (ABCG5/8), or processed by synthesis of HDL-C and release into circulation directly *via* ABCA1.^87^

#### Cholesterol uptake, esterification and efflux in macrophages

Macrophage cholesterol homeostasis plays an essential role in the development of atherosclerosis.^88^ Excessive uptake of cholesterol, excessive intracellular cholesterol esterification and impaired cholesterol efflux can drive differentiation of macrophages into foam cells and formation of atherosclerotic plaques in the vessel wall.^89^ Macrophage cholesterol uptake is mainly mediated through multiple scavenger receptors, the molecules lack of SRE, rather than LDLR.^90^ Thus, without feedback control mechanisms, macrophage scavenger receptors may uptake cholesterol unlimitedly in patients with hypercholesterolemia. Macrophages scavenger receptors include scavenger receptor A1 (SR-A1), SR-BI, lectin-like oxidized LDL receptor 1 (LOX-1), CD36 and so on. Among them, SR-A1 and CD36 mediate most of the endocytosed LDL (75–90%).^91–93^ Meanwhile, compared with LDL, these scavenger receptors have higher affinity for modified LDL, particularly the oxidatively modified LDL (oxLDL).^94^ In atherosclerosis, expression of SR-A1, LOX-1, and CD36 in macrophages are increased. The activated scavenger receptors can elevate the levels of pro-inflammatory cytokines, oxLDL, lysophosphatidylcholine, advanced glycosyl end products (AGEs), and vasopressors in macrophages, further promoting cholesterol accumulation and foam cell formation.^89^

After endocytosis, lipoproteins will be hydrolyzed in lysosomes by action of lysosomal acid lipase (LAL, also named as cholesterol ester hydrolase or lipase A) to generate FC. The excess FC is then esterified in the ER by ACAT1, which can attenuate FC cytotoxicity. The cholesteryl ester (CE) can be stored as lipid droplets (LD) in the cytoplasm.^95^ However, if ACAT1 esterifies too much FC to CE, the excessive lipid accumulation can also result in conversion of macrophages into foam cells. Therefore, ACAT1 is also considered as a possible effective target in reduction of foam cells. Consistently, deletion or inhibition of ACAT1 in macrophages has an inhibitory effect on atherosclerosis in mouse models.^96–99^ However, the ACAT1 inhibitors failed to produce desired athero-protective effects in clinic, which may be due to excessive accumulation of FC in cells and generation of lipotoxicity, resulting in profound cell death.^100–102^ Macrophages are not able to degrade sterols, thus, CE needs to be hydrolyzed into FC for efflux. Neutral cholesteryl ester hydrolase (NEH) hydrolyzes CE to release FC.^103^ There are three main NEHs, of which carboxylesterase 1 (CES1) and neutral cholesteryl ester hydrolase 1 (NCEH1) are mainly expressed in human macrophages for CE hydrolysis.^104,105^

When cholesterol is abnormally accumulated in macrophages, the cells acquire a defense mechanism to combat the deleterious effects caused by excessive cholesterol uptake by promoting cholesterol efflux *via* the mechanisms involving simple diffusion, SR-BI-facilitated diffusion, and ABCA1 and/or ABCG1-mediated efflux.^95,106^ Among them, the simple diffusion is a passive process regulated by cellular cholesterol concentrations and dominates the cholesterol efflux in normal cells, whereas in cholesterol-overloaded cells, ABCA1 and ABCG1 are critical for cholesterol efflux.^107^ The cholesterol efflux mediated by ABCA1 is the most efficient way for macrophages to remove intracellular cholesterol. ABCA1 can bind to ApoA-I and drive cholesterol flow to ApoA-I to form nascent pre-β-HDL particles. Expression of ABCA1 strongly influences the level of plasma HDL-C.^108,109^ In 2017, the elucidation of the crystal structure of ABCA1 established that ABCA1 forms hydrophobic tunnels to transport lipids, but the mechanism for cholesterol delivery from ABCA1 to ApoA-I still remains incompletely clarified.^110^ In contrast to ABCA1, ABCG1 is not directly bound to the empty ApoA-I, instead, it mediates the cholesterol flow to pre-βHDL particles formed by ABCA1-mediated cholesterol efflux.^111,112^ Meanwhile, expression of ABCA1 and ABCG1 are strictly controlled by liver X receptors (LXRs). The increased cellular cholesterol levels promote production of hydroxysteroids, the endogenous LXR agonizts, thereby increasing ABCA1 and ABCG1 expression.^113^ Compared to ABCA1 and ABCG1, SR-BI was initially recognized as the receptor for HDL-mediated CE uptake and only a minor contributor in cholesterol efflux.^114^

#### Liver cholesterol transport and RCT

The liver is the main site of cholesterol metabolism. It is also the most essential organ for effective RCT. In general, cholesterol is transported to the liver from peripheral cells (especially macrophages) by HDL particles, which is considered to be the first step in RCT. Thus, HDL particles play a key role for lipid homeostasis as lipid receptors in lymphatic fluid and plasma.^115^ HDL is a smaller lipoprotein with a core of ApoA-I loaded with CE and TG, and an outer layer of phospholipids (PL) which allows the solubilization of FC to complete the transport.^116^ According to the particle size, HDL can be divided into two subclasses, one is HDL2, which is rich in lipids with larger volume, and the another one is HDL3, which is rich in proteins with smaller volume.^117,118^ Lipid-poor ApoA-I synthesized in hepatocytes or enterocytes accepts FC transported by ABCA1 from peripheral cells to form pre-β HDL particles.^119,120^ Afterwards, lecithin cholesterol acyltransferase (LCAT) and phospholipid transfer protein (PLTP) further mature pre-β-HDL particles to produce HDL3, and HDL3 acts as an acceptor for FC discharged by ABCG1 and/or SR-BI to produce HDL2 finally.^121–123^ Among them, LCAT mediates the cleavage of fatty acids at the *sn*-2 position of phospholipids and transesterification to the 3-β-hydroxyl group on the A ring to form CE.^124,125^ PLTP mediates the transfer of PL from ApoB-containing lipoproteins to HDL to facilitate FC influx.^126^ The liver selectively absorbs lipids from HDL *via* SR-BI and transfers CE to bile for intestinal excretion to complete the entire RCT process.^114^

Based on the key role of HDL in RCT, it is widely believed that HDL-C is a “good” cholesterol to the extent that it inhibits the progression of atherosclerosis. The results of several clinical studies found that interventions to increase plasma HDL-C concentrations by inhibiting cholesteryl ester transfer protein (CETP) or using niacin did not reduce the development of atherosclerosis.^127–129^ The esterification of cholesterol by LCAT is critical for the inhibition of atherosclerosis by RCT, whereas the rate of clearance of FC in HDL is much higher than that of LCAT esterification, due to the fact that FC can enter the liver directly through cell membrane without LCAT esterification, which may also explain the controversial protective effects of interventions targeting LCAT against atherosclerosis.^130,131^ Meanwhile, several large studies also found a U-shaped curve between HDL-C concentrations and all-cause mortality in ASCVD patients, with both too low and too high levels of HDL-C leading to an increased risk of ASCVD.^132,133^ In addition, the HDL collected from patients with CVD or chronic kidney disease lose the capacity of RCT by promoting LOX-1 mediated vascular dysfunction. Patients suffering from ASCVD with high HDL-C tend to lack PL in HDL, which leads FC to flow back to macrophages to facilitate foam cell formation.^131^ Therefore, maintaining the normal function of HDL rather than simply increase of HDL-C concentrations is the more important aspect of RCT therapy.

In addition to uptake of HDL-C via SR-BI, the liver also uptakes LDL-C *via* LDLR to directly remove atherosclerotic lipoproteins from the plasma. In the hepatic ER, ApoB-100 is the main apolipoprotein to synthesize very low-density lipoprotein (VLDL) to transport endogenous TG and cholesterol. When TG contained in VLDL is hydrolyzed by LAL, the remaining particles are converted to LDL.^134^ LDL is the primary carrier of endogenous cholesterol for transport, and two-thirds of TC in plasma binds to LDL to form LDL-C, which is absorbed and converted through hepatic LDLR. In humans, CE in mature HDL particles is also exchanged to LDL or VLDL particles by CETP, then the CE in these particles is absorbed by LDLR.^135^ In mammals, LDLR is highly expressed in the liver to mediate more than 70% of LDL-C clearance.^136^ LDLR deficiency is the most common cause of FH, in which patients present with markedly elevated LDL-C level and early ASCVD onset.^137,138^ LDLR transcription is mainly regulated by SREBP2 and can respond to changes of intracellular cholesterol.^90^ PCSK9 reduces LDLR expression in the post-translational manner. It binds to LDLR to induce LDLR entry into cells for lysosomal degradation and inhibits the ability of LDL uptake in the liver.^34^ Similarly, the inducible degrader of LDLR (IDOL) can also promote LDLR degradation through polyubiquitination and lysosomal degradation pathways.^139^ A recent cognitively subversive study found that HDL can bind to PCSK9 to increase PCSK9 activity and accelerate PCSK9-mediated LDLR degradation. This study further elucidates the interaction between circulating lipoproteins and PCSK9, and provides new therapeutic ideas for targeting PCSK9. Furthermore, coagulation factor prekallikrein (PK) was recently reported to regulate plasma cholesterol levels *via* binding to LDLR to induce its lysosomal degradation. Deficiency of PK stabilizes LDLR protein expression, promotes hepatic LDL-C clearance and inhibits atherosclerosis in mice.^140^ All the evidence above suggest that LDLR still represents a promising therapeutic target for ASCVD treatment.

### Cholesterol utilization and excretion

#### Utilization of cholesterol

As an important component in biological membranes, cholesterol accounts for more than 20% of lipids in membranes.^141,142^ Cholesterol is a largely hydrophobic molecule, and only the 3β-hydroxyl portion is a polar group, thus, cholesterol is amphiphilic and can be oriented in the phospholipid bilayer perpendicular to the membrane surface.^143–145^ In domains or pools of biological and model membranes, cholesterol is usually non-randomly distributed, in which many structural domains are thought to be important for maintaining membrane structure and function.^146–148^ Besides participating in the composition of biological membranes, cholesterol is the essential precursor for synthesis of oxysterols. Formation of oxysterols is the step converting cholesterol into more polar compounds, which can facilitate elimination of cholesterol. Meanwhile, oxysterols have different important physiological roles. Some oxysterols can activate LXR to regulate cholesterol efflux from macrophages, and some of them can bind to INSIG to regulate SREBP2 maturation, therefore, these oxysterols play an important role to maintain cholesterol homeostasis.^149,150^ Oxidoreductases, hydrolases and transferases are the three main enzymes involved in the metabolism of oxysterols. Among the oxidoreductases, the enzymes catalyzing formation of oxysterols, cytochrome P450 (CYP) has been relatively well studied. The earlier identified two enzymes, cholesterol 7α-hydroxylase (CYP7A1) and cholesterol 27-hydroxylase (CYP27A1), participate in bile acid synthesis by producing 7α-hydroxycholesterol (7α-OHC) and 27-OHC, respectively. In addition, formation of OHC by CYP7A1 is the rate-limiting step for bile acid production.^151,152^ Cholesterol 25-hydroxylase (CH25H), another key oxidoreductase, does not belong to the CYP450 superfamily.^153^ CH25H catalyzes the production of 25-OCH, which is capable of acting as an agonist of estrogen receptor α.^154^ In addition to the aforementioned enzymes, there are many other enzymes that catalyze synthesis of specific oxysterols, indicating the mechanisms for oxysterol production/metabolism still need further investigation. Moreover, cholesterol is the precursor for generation of all steroid hormones. Various steroid-producing tissues (adrenal glands, testes, ovaries) and brain cells produce steroid hormones. The inner mitochondrial membrane contains CYP450, a key enzyme to convert cholesterol to pregnenolone. Subsequently, pregnenolone leaves the mitochondria and is further catalyzed by the corresponding enzyme in the ER as a substrate for steroid hormone synthesis.^155^

#### Excretion of cholesterol

The elimination of cholesterol from the liver to remove excess cholesterol is considered as the final step in RCT. Both ABCG5/8-mediated hepatobiliary secretion and transintestinal cholesterol excretion (TICE) pathways mediate this process.^156^

During the hepatobiliary cholesterol secretion, ABCG5 and ABCG8 form a heterodimer to mediate cholesterol excretion into the bile and intestinal lumen.^157,158^ At the same time, bile salt is the main acceptor for ABCG5/8-mediated hepatic cholesterol efflux.^159,160^ Bile acids secreted from hepatocytes will combine with glycine or taurine to form bile salts. CYP7A1 is the key enzyme for bile acid synthesis, converting cholesterol (usually from LDL particles) to 7α-OCH through a multienzyme process.^151^ Subsequently, CYP450 enzymes including CYP8B1, CYP27A1 and CYP7B1 located on the ER of hepatocytes are involved in many of the subsequent reactions.^161–163^ Lee et al. determined the structure of ABCG5/8 heterodimer by extracting the crystals of phospholipid bilayer ABCG5 and ABCG8. The structure shows that the transmembrane structural domain of this heterodimer is coupled to the nucleotide binding site through different interaction networks between the active and inactive ATPases, indicating the catalytic asymmetry of ABCG5 and ABCG8 protein.^164^ Similar to ABCA1 and ABCG1, ABCG5 and ABCG8 are also transcriptionally regulated by LXR. When hepatic cholesterol is overloaded, increased oxysterols activate LXR and enhance expression of ABCG5/8.^165,166^

Another non-biliary TICE pathway of cholesterol excretion refers to cholesterol secretion directly to the proximal small intestine from the blood *via* enterocytes.^167^ In both rodents and humans, TICE mediates about 30% of the total fecal cholesterol excretion and plays a significant role in cholesterol efflux.^166,168,169^ When the synthesis of bile acids/salts is abnormal in the body, TICE takes on more to maintain normal cholesterol efflux.^170^ Stöger et al. found that interleukin 10 (IL-10) receptor 1 (IL-10R1)-deficient LDLR^−/−^ mice showed an increase in TICE-mediated cholesterol efflux and inhibited atherosclerosis, suggesting that TICE may have potential anti-atherosclerotic effects.^171^ Since enhanced hepatobiliary cholesterol secretion has the side effect of causing gallstones, promoting TICE may be a new idea to combat atherosclerosis.^172^ However, the molecular mechanism of TICE has not been fully clarified, and various factors of cholesterol metabolism can affect TICE to some extent, which is a direction worthy of the future attention.^173–175^

### Epigenetic modulation of cholesterol metabolism

In addition to the classical models of cholesterol metabolism regulation described above, the recent evidence has revealed multiple epigenetic regulatory mechanisms involved in uptake, synthesis and efflux of cholesterol, such as histone acetylation, DNA methylation and ubiquitylation.

Bromodomain and extra-terminal domain (BET) proteins are epigenetic readers that are recruited to chromatin in the presence of acetylated histones, thereby regulating gene expression. Inhibition of BET effectively reduces intracellular cholesterol levels by significant regulating genes involved in cholesterol biosynthesis, uptake and intracellular trafficking, indicating that most of the genes involved in regulation of cholesterol homeostasis can be regulated by epigenetic mechanisms.^176^

Intestinal NPC1L1 is differentially expressed in the gastrointestinal tract, with much higher levels in small intestine than colon, which is associated with high levels of methylation upstream of NPC1L1 gene start site in the colon, suggesting a possible reduction in cholesterol uptake and prevention of atherosclerosis by alteration of DNA methylation.^177^ Whereas data on the epigenetic regulation of ABCG5/8 in the intestine are very limited. A few studies in mouse liver suggest that the common promoters of ABCG5/8 are acetylated and unmethylated. Histone methyltransferase SET domain 2 (SETD2) catalyzes trimethylation on H3K36 (H3K36me3), and recent studies have revealed that STED2 is involved in regulating hepatic ABCA1 expression and cholesterol efflux homeostasis.^178^

Brahma related gene 1 (BRG1, a chromatin remodeling protein) interacts with SREBP2 and recruits histone 3 lysine 9 (H3K9) methyltransferase (KDM3A) at the promoter of SREBP2 target genes to regulate the transcription of genes involved in cholesterol synthesis.^179^ Euchromatic histone-lysine N-methyltransferase 2 (EHMT2) is a histone methyltransferase that catalyzes H3K9 of SREBP2 monomethylation and dimethylation (H3K9me1 and H3K9me2, respectively). Inhibition of EHMT2 is able to directly induce SREBP2 expression by reducing H3K9me1 and H3K9me2 at the promoter.^180^ At the same time, the complex of histone acetylase cAMP response element binding protein 1 (CREB) binding protein (CBP)/P300 bromodomain acetylates the conserved lysine residues of SREBP protein, thereby preventing the ubiquitination and degradation of SREBP, prolonging its residence time in the nucleus and promoting its transcriptional activity. In contrast, sirtuin 1 (SIRT1) can antagonize the action of CBP/P300 by deacetylating SREBP.^181^ Thus, the transcriptional activity of SREBP is regulated by multiple epigenetic mechanisms, keeping it in a complex dynamic equilibrium.

Various genes associated with cholesterol elimination, such as CYP7A1, CYP46A1 and CH25H, have been shown to be differentially regulated epigenetically. CYP7A1 can be regulated by indirect negative feedback from small heterodimeric chaperone (SHP) proteins. Several studies have identified the presence of BRG1-mediated chromatin remodeling and SIRT1-mediated histone deacetylation at the SHP promoter, which further regulates CYP7A1 expression.^182,183^ CYP46A1 is regulated by the acetylation status of histones. in vitro, treatment of hepatocytes with deacetylase inhibitor, trichostatin A, significantly upregulates CYP46A1 mRNA levels.^184^ The signal transducers and activators of transcription 1 (STAT1) pathway regulates CH25H expression, which also requires the involvement of histone acetylation.^185,186^

The epigenetic regulation of cholesterol homeostasis is a promising research area, with multiple genes being differentially regulated. Research in this area could provide the basis for transcriptional therapies for related diseases, drug development and the clinical application of dietary epigenetic modulators. However, there are still many questions and gaps in this field that need to be solved.

## Cholesterol-related diseases and interventions

### Cholesterol and ASCVD

#### Role of cholesterol in the development of ASCVD

**Fig. 4:**
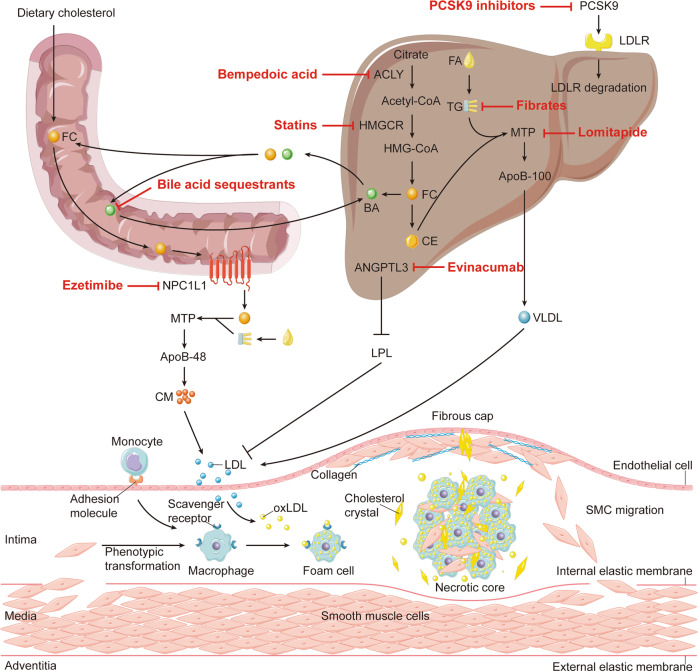
Inhibition of atherosclerosis by cholesterol-lowering interventions. Bempedoic acid and statins reduce acetyl-CoA and HMG-CoA production by inhibiting ACLY and HMGCR, respectively, thereby lowering cholesterol synthesis. Ezetimibe inhibits intestinal uptake of cholesterol by inhibiting NPC1L1. PCSK9 inhibitors reduce LDLR degradation by inhibiting PCSK9 expression/function. Bile acid sequestrants bind to BA in the small intestine, thus preventing BA from being reabsorbed into the liver. Lomitapide reduces the assembly of ApoB-containing lipoproteins in intestine and liver. Evinacumab restores LPL activity by inhibiting ANGPTL3. Fibrates reduce TG levels. All of the above interventions can reduce plasm LDL-C levels, which is the base for the development of atherosclerosis. The arterial wall consists of three layers: adventitia, media, and intima. The outermost layer, adventitia, is mainly composed of connective tissues. The middle layer, media, consists of smooth muscle cells. The innermost layer, intima, is bounded by endothelial cells (ECs) on the inner side of the lumen and internal elastic membrane on the outer side. Atherosclerotic plaques form in the intima. In the early stage of atherosclerosis, LDL particles enter the intima through EC layer and undergo oxidation and other modifications to form oxLDL, which makes it pro-inflammatory and immunogenic. ECs secrete adhesion molecules and chemokines after activation, and monocytes circulating in the blood bind to adhesion molecules and enter the intima under the promotion of chemokines. After entering the intima, the infiltrated monocytes then differentiate into macrophages and express scavenger receptors to bind and internalize oxLDL to form foam cells. A subset of smooth muscle cells from the media can also differentiate into a macrophage-like phenotype, which in turn phagocytoses oxLDL to form foam cells. As the lesion progresses, dead foam cells and SMCs aggregate with free lipoprotein and cholesterol crystals in the intima to form a necrotic core. SMCs migrate to endothelium and forms fibrous cap during the evolution of atherosclerotic plaque. As cholesterol crystals grow, they eventually penetrate the intima, causing plaque instability and further rupture of the plaques. Acetyl CoA acetyl coenzyme A, ACLY ATP citrate lyase, ANGPTL3 angiopoietin-like protein 3, BA bile acid, CE cholesteryl ester, CM chylomicron, EC endothelial cell, FA fatty acid, FC free cholesterol, HMGCR 3-hydroxy-3-methylglutaryl coenzyme A reductase, HMG-CoA 3-hydroxy-3-methylglutaryl coenzyme A, LDL low-density lipoprotein, LDLR LDL receptor, LPL lipoprotein lipase, MTP microsomal triglyceride transfer protein, NPC1L1 Niemann-Pick C1 like 1, oxLDL oxidatively modified low-density lipoprotein, PCSK9 proprotein convertase subtilisin/kexin type 9, SMC smooth muscle cell, TG triglyceride, VLDL very low-density lipoprotein

Deregulated cholesterol metabolism leads to the development of multiple human diseases, among which atherosclerosis is the major one. Atherosclerosis is the process of accumulation of lipids and fibrous substances in arterial intima, and results in ASCVD as the main cause of death worldwide.^187^ The main reason of atherosclerotic plaque formation is the excessive accumulation of cholesterol-rich lipoproteins in the arterial intima (Fig. 4).^187,188^

Accumulation and retention of ApoB-containing lipoproteins in the arterial intima are thought to induce atherosclerosis.^189^ Recent evidence has suggested that SR-BI in endothelium is an important scavenger receptor that promotes LDL transcytosis/accumulation and atherosclerosis.^190^ Retained LDL particles activate an initial immune response in the endothelium, thus, triggering chronic inflammation by releasing monocyte chemotactic protein-1 (MCP-1) and some other inflammatory factors.^191^ Endothelial chemokines and cytokines including MCP-1, intercellular adhesion molecule 1 (ICAM1), vascular cell adhesion molecule 1 (VCAM1), E-selectin, macrophage colony stimulating factor (M-CSF), IL-18 and tumor necrosis factor α (TNF-α), further promote monocyte migration to endothelium.^192,193^ Monocytes can differentiate into macrophages after migration to the underneath of endothelium, where macrophages bind and internalize modified LDL or lipoprotein residues in the intima to form foam cells.^194^

Foam cell formation is the major hallmark of early lesions in atherosclerosis.^89^ Macrophages differentiated from circulating monocytes are the main source of foam cells.^195,196^ A small number of foam cells can be derived from endothelial cells (ECs) and/or vascular smooth muscle cells (VSMCs). ECs may differentiate into VSMC-like cells while VSMCs will further differentiate into macrophage-like cells, which become foam cells after lipid overload.^197^

LDL must undergo oxidative modification before it can be rapidly taken up by macrophages and accumulated in lysosomes.^198^ LOX-1 is one of the scavenger receptors and highly expressed in ECs, which binds oxLDL and transfers it to the intima infiltrated by macrophages. Next, macrophages bind oxLDL through scavenger receptors including SR-A1, CD36, and LOX-1.^89^

The formation of CE is an important part in the transition of macrophages to foam cells. Disruption of the balance between esterification and de-esterification results in accumulation of CEs in macrophages, leading to foam cells formation.^17^ As an important part of lipoprotein metabolism, RCT can prevent foam cell formation. Imbalanced conversion between CE and FC and dysregulation of HDL function lead to formation of cholesterol crystals.^199^ As cholesterol crystals grow and accumulate in the extracellular space of the plaque necrosis core, it eventually reaches and penetrates the arterial intima.^200^ This will lead to increased plaque instability, which in turn causes plaque rupture and further thrombus formation.^17^

#### Cholesterol-lowering intervention therapy

LDL-C is involved in the occurrence and development of atherosclerosis, indicating LDL-C is the main risk factor for ASCVD. More and more studies show that lower LDL-C levels are better for cardiovascular system.^201,202^ In the following sections, we will discuss the drugs that possess cholesterol-lowering capacities (Table 1).

**Table 1:** The application of clinical cholesterol-lowering interventions on ASCVD

Clinical intervention	Target	Clinical efficacy	Adverse effects
Statins	HMGCR	↓ LDL-C, ↑ HDL-C	Myopathy
Ezetimibe	NPC1L1	↓ LDL-C, ↑ HDL-C, ↓TG	None
PCSK9 inhibitors	PCSK9	↓ LDL-C, ↑ HDL-C, ↓Lp(a)	Injection site reactions
Bempedoic acid	ACLY	↓ LDL-C, ↑ HDL-C	Hyperuricaemia
Bile acid sequestrants	Bile acids	↓LDL-C, ↑ HDL-C	Gastrointestinal adverse reactions
Lomitapide	MTP	↓ LDL-C, ↓ Lp(a), ↓TG	Gastrointestinal adverse reactions
Evinacumab	ANGPTL3	↓ LDL-C, ↓ TG	Injection site reactions, flu-like illness, headache, urinary tract infection and limb pain
Fibrates	PPARα	↓ LDL-C, ↑ HDL-C, ↓TG	Gastrointestinal adverse reactions
Lipoprotein apheresis	Plasma lipoprotein	↓LDL-C, ↓ Lp(a), ↓TG	None

##### Statins

Statins are competitive HMGCR inhibitors, which can effectively reduce the level of plasma cholesterol, especially LDL-C levels. Statins represent the mainstream therapy for CVD.^203–206^ Historical studies have confirmed that statins are able to reduce the incidence of CVD by 23% which leads to statins as the first choice for the treatment of hypercholesterolemia.^207^ Mevastatin is the first statin discovered in the world, and it was isolated from fungal species *Penicillium citrinum*.^208^ But till the 1990s, the landmark Scandinavian Simvastatin Survival study (4S) showed convincing results that support the use of statins to reduce cholesterol and CVD.^209^ By 2020, at least nine different statins have been developed, among which seven have been approved in USA and one has been withdrawn from the market.^203^ Statins inhibit HMGCR activity by competitively binding to the enzymatic site of HMGCR, resulting in decreased cholesterol synthesis and reduced plasma cholesterol levels.^210^ Low plasma cholesterol levels in turn increase hepatic LDLR expression via the SREBP2-dependent pathway. The increased LDLR expression in hepatocytes speeds up the uptake and clearance of LDL-C from plasma, another important mechanism of statins improving cholesterol metabolism systematically.^211^ However, some studies have shown that statin can also induce PCSK9 expression since PCSK9 also contains SRE in its promoter. The increased PCSK9 expression substantially attenuates the expected efficacy of statins on cholesterol lowering.^212,213^

Without the influence of PCSK9, the extent of LDL-C reduced by statins should be dose-dependent and may vary among different statins. According to the effect of lowering LDL-C, different types and doses of statin therapy are divided into three intensities: low, moderate and high. Low-intensity is defined as a daily dose of statin that can reduce LDL-C < 30%; moderate-intensity is indicated as reducing LDL-C to 30–50%; and high-intensity is to reduce LDL-C ≥ 50%.^214^ A meta-analysis showed a 10% reduction in all-cause mortality for per 1 mmol/l (equivalent 39 mg/dl) reduction in LDL-C, mainly due to a reduction in deaths from CVD.^207^ Further meta-analysis showed that statins can reduce all-cause mortality and the risk of cardiovascular events, regardless of age and sex.^215,216^ Even in patients with low cardiovascular risk, statins could reduce all-cause mortality and cardiovascular events.^217^

In addition to reduction of LDL-C, statins have been demonstrated to have many other beneficial effects, known as the pleiotropic effects of statins.^218,219^ Statins have been reported to elevate HDL-C, which also varies with dose among different statins.^220^ However, when LDL-C is below a certain level, statin-elevated HDL-C has little effect on disease regression.^221^ The anti-inflammatory and antioxidant effects of statins may also make contributions to prevention and/or reduction of ASCVD, at least confirmed by in vitro and animal studies. However, the clinical significance of these positive effects on ASCVD may need more exploration.^222,223^

Although the efficacy of statins in lowering LDL-C and treating ASCVD is unquestionable, there are still many controversies regarding the application of statins.^224^ Myopathy is one of the most common clinical adverse reactions caused by statins.^225^ The most severe form of statin-associated muscle symptoms (SAMS), rhabdomyolysis, is characterized by severe muscle pain, muscle necrosis, and myoglobinuria, which can lead to kidney failure or death.^226^ However, the nocebo effect may outweigh the side effects caused by the statins themselves.^227^ Thus, in all international guidelines, the availability of statins for the secondary prevention of ASCVD is consistent in patients without statins intolerance or adverse reactions, and the benefits of statins treatment are supported by a large amount of data.^228^ When it comes to primary prevention, the international guidelines for the treatment of isolated adult patients with elevated LDL-C (defined as ≥190 mg/dL) have not yet reached consensus. At the same time, the application of statins in patients with chronic kidney disease, diabetes, the elderly over 75 years old, and patients with heart failure also demonstrated mixed results.^229–232^ For those patients with intolerance to the recommended-intensity statins due to the adverse effects or those who do not achieve LDL-C reducing goals, the non-statin lipid-lowering drugs added to the maximally tolerated statins can be recommended.^233,234^

##### Ezetimibe

Ezetimibe is an intestinal cholesterol absorption inhibitor, which can block intestinal uptake of cholesterol by interacting with NPC1L1 without effect on absorption of TG and fat-soluble vitamins.^235,236^ In addition to lowering plasma cholesterol levels, similar to statins, ezetimibe also up-regulates LDLR expression in the liver, thereby enhancing LDL-C clearance.^237^ Experiments have also shown that ezetimibe may reduce inflammation in atherosclerotic plaques by increasing LDL-C breakdown and promoting fecal excretion of LDL-derived cholesterol.^238,239^

Ezetimibe is a good option for patients with contraindications, statin intolerance and/or insufficient LDL-C reduction.^235^ Clinical studies and meta-analyses show that ezetimibe monotherapy significantly reduces LDL-C and TC levels. It also slightly increases HDL-C levels in patients with hypercholesterolemia.^237,240^ LDL-C lowering treatment with ezetimibe reduces the risk of cardiovascular events in patients aged ≥75 years with elevated LDL-C.^241^ In a rabbit model of plaque erosion, ezetimibe lowered serum oxysterols, thereby reducing atherothrombotic complications following superficial plaque erosion.^242^

In order to achieve better therapeutic effects, ezetimibe is often used in combination with a statin. In 2018, Ezetimibe was the most prescribed non-statin lipid-lowering therapy. In patients treated with statins, the addition of ezetimibe reduced LDL-C by an additional 23.8%, and fixed-dose combination (FDC) therapy reduced LDL-C by an additional 28.4% compared with statin therapy alone. However, treatment outcomes vary widely among individuals that only a small percentage of patients achieved recommended LDL-C levels (FDC, 31.5%; separate pills, 21.0%).^243^ In addition, bempedoic acid plus ezetimibe FDC together with maximally tolerated statin therapy also significantly lowered LDL-C and had a favorable safety profile.^244^ It has been reported that co-administration of ezetimibe with a bile acid sequestrant can reduce LDL-C by an additional 10–20%.^245^ The combination of ezetimibe and PCSK9 inhibitor may have an additional effect in cholesterol lowering.^246^

Notably, age, gender, or race do not affect the pharmacokinetics of ezetimibe, and no dose adjustment was required in patients who had mild hepatic impairment or mild to severe renal impairment.^235^ Furthermore, ezetimibe also shows favorable drug interaction characteristics and has little effect on plasma levels of statins. In addition, the bioavailability of ezetimibe is not significantly affected by concurrent statin administration.^247^

##### PCSK9 inhibitors

The discovery of PCSK9 provides a new idea for controlling plasma LDL-C levels. PCSK9 inhibitors can increase LDLR expression by attenuating PCSK9 expression/function, leading to the lowering plasma LDL-C.^248^ In addition, it has been reported that inflammatory state could promote PCSK9 expression and increased PCSK9 would up-regulate LOX-1 expression, thus promoting oxLDL uptake and accelerating the progression of atherosclerosis.^249,250^ At present, there are three approved PCSK9 inhibitors, among which alirocumab and evolocumab are the full human monoclonal antibodies, and the third one, inclisiran, is a double-stranded siRNA.^251,252^

In meta-analysis, evolocumab and alirocumab could significantly reduce cardiovascular events, but had no significant effect on cardiovascular mortality.^253–256^ Evolocumab and alirocumab, either alone or in combination with statins or other lipid-lowering drugs, can reduce LDL-C levels by an average of 60%.^235^ When evolocumab and alirocumab were used in combination with the high-intensity statins, there was an additional 46–73% reduction in LDL-C compared to placebo, and an additional 30% reduction compared to ezetimibe.^235^ Inclisiran is a novel PCSK9 inhibitor, which was approved for treatment of ASCVD by US FDA in 2021.^252^ In the two phase 3 trials of inclisiran in the patients with elevated LDL-C, subcutaneous injection of inclisiran once every 6 months resulted in a 50% reduction in LDL-C levels.^257^ Adverse events at the injection site of inclisiran were more frequent than placebo, but the reaction was usually mild.^257^ Recently, a study showed that inclisiran inhibited foam cell formation by inhibiting oxLDL uptake by RAW264.7 macrophages, which was associated with activation of peroxisome proliferator-activated receptor γ pathway. This observation may provide new insights into the cholesterol-lowering mechanism of inclisiran.^258^

Itching at the injection site and flu-like symptoms are the most common side effects of PCSK9 inhibitors.^259^ PCSK9 inhibitors are effective. However, given the high cost and limited data on the long-term safety, they may be only cost-effective in patients with high risk of ASCVD, while not be available in some areas with no enough medical resources.^235^ Therefore, lower-cost alternative drugs need to be developed.

##### Bempedoic acid (ETC-1002)

Bempedoic acid, an inhibitor of ACLY, is the first FDA-approved non-statin oral cholesterol-lowering drug in nearly 20 years.^40,260^ In fact, bempedoic acid is a prodrug and needs to be converted into bempedoic acid-CoA thioester, the active form of ACLY inhibitor, by very long-chain acyl-CoA synthetase-1 (ACSVL1).^261^ Interestingly, expression of ACSVL1 is tissue-dependent with little in the muscle and high in the liver. Therefore, inhibition of ACLY activity by bempedoic acid administration simply occurs to the liver, thereby avoiding the muscle-related side effects.^262^ ACLY inhibition can also upregulate LDLR expression, which can make additional contributions to the reduction of plasma LDL-C levels.^263^ Studies have shown that in high-fat and high-cholesterol diet-fed mice, in addition to inhibition of cholesterol synthesis and activation of LDLR expression, bempedoic acid also reduces inflammation by directly inhibiting ACLY and activating AMPKβ1 activity, thereby potently preventing atherosclerosis.^262,264^

The CLEAR trials showed that adding bempedoic acid to current cholesterol-lowering therapy can further reduce LDL-C levels in patients with high risk for CVD.^244,263,265^ When combined with statins, ezetimibe lowered LDL-C by an additional 25%, while bempedoic acid add-on therapy lowered LDL-C by an additional 16%.^266,267^ This finding contrasted with the findings of the monotherapy arms in phase 3 trial, in which LDL-C was reduced by ~30% by bempedoic acid and ~21% by ezetimibe alone.^268^

The application of bempedoic acid may cause an increase in serum uric acid and increase the risk of tendon rupture, so patients with gout or a history of tendon disease should avoid using bempedoic acid.^269^ In view of some drug interactions found in clinical trials, the administration of drugs containing bempedoic acid is not recommended when using simvastatin at a dose >20 mg or pravastatin at a dose >40 mg.^268^

For patients at high risk of ASCVD, bempedoic acid alone or in combination with ezetimibe can be considered as an additional treatment of statins.^270^ Given the high cost of PCSK9 inhibitors, the use of bempedoic acid would be a higher priority than PCSK9 inhibitors, but lower than ezetimibe based on the limited data on the overall efficacy. Nonetheless, the combination of bempedoic acid or ezetimibe with statins is suggested for the patients who require greater LDL-C lowering than either drug alone. At present, the lipid-lowering ability of bempedoic acid is clear, but whether it can reduce the risk of ASCVD remains unknown, which needs further study.

##### Bile acid sequestrants

Bile acid sequestrants (BAS) are macromolecular polymers which can bind to bile acids in the small intestine, thus, BAS can prevent bile acids from being reabsorbed back into the liver.^271^ Due to bile depletion in the liver, more bile acids than usually required are synthesized from liver cholesterol, which increases the demand for cholesterol in the liver, leading to increased LDLR expression and clearance rate of circulating LDL-C.^272^ Three types of BAS have been approved for clinical use: cholestyramine, colestipol and colesevelam hydrochloride. The past clinical trials demonstrated that BAS was effective in lowering LDL-C and reduction of the risk of cardiovascular events in hypercholesterolemic patients.^272–275^

Even low-dose BAS could also cause gastrointestinal adverse reactions, which limits its application. It has been reported that use of BAS can reduce the absorption of intestinal fat-soluble vitamins and sometimes increase the level of circulating TG in some patients.^235^ In addition, BAS interacts with several commonly used drugs, so it must be used with caution in combination therapy. Among them, colesevelam is well tolerated and has less interaction with other drugs, thus, it can be used concurrently with drugs for other kinds of disease treatment.^276^

##### Lomitapide

Lomitapide is an oral microsomal TG transfer protein (MTP) inhibitor, which can reduce the assembly of lipoproteins containing ApoB in intestine and liver, so the reduction of LDL-C levels by MTP inhibitors is independent of LDLR.^277^ Lomitapide has been proved to reduce LDL-C in homozygous FH (HoFH) patients by nearly 50% in combination with other lipid-lowering drugs.^278^

In a real-world European study, lomitapide has been proved to be a very effective adjuvant drug to reduce LDL-C in HoFH patients for the longest follow-up period so far.^279^ As lomitapide blocks MTP, it leads to impaired intestinal fat transport, making gastrointestinal symptoms as the most common adverse event in patients.^280^ In terms of safety, lomitapide-related hepatic steatosis may not indirectly increase the risk of liver fibrosis, and the data suggest that lomitapide may reduce cardiovascular events in HoFH patients.^279^

##### Evinacumab

Evinacumab is a human monoclonal IgG4 antibody neutralizing angiopoietin-like protein 3 (ANGPTL3). ANGPTL3 is a protein secreted by the liver, which inhibits activity of lipoprotein lipase and endothelial lipase, the two lipases involved in the regulation of lipid hydrolysis in serum.^281^ Inhibition of ANGPTL3 by evinacumab restores activity of the two lipases, thus reducing serum cholesterol and TG levels.^282^

In 2021, evinacumab was approved in USA as an adjunctive cholesterol-lowering treatment for FH in adults and children 12 years of age or older. The previous clinical trials showed that evinacumab reduced TC and LDL-C by 45–55% in HoFH patients already receiving maximum tolerated doses of lipid-lowering drugs.^282^ An animal study showed that alirocumab, evinacumab, and atorvastatin triple therapy significantly reduced hyperlipidemia and atherosclerosis.^283,284^ Currently, no randomized clinical trials demonstrate that evinacumab can reduce cardiovascular events, so the further research is needed.

Frequent adverse events of evinacumab include mild local injection reaction, flu-like illness, headache, urinary tract infection and limb pain.^285^ In addition, no clinically apparent liver injury or serious hepatic adverse events attributable to treatment were reported.

##### Fibrates

Fibrates are PPARα agonizts, which can increase HDL-C levels and decrease TG levels in plasma by regulating molecules related to lipid metabolism.^286^ The clinical effects of fibrate class on blood lipids are different, but are estimated to reduce TG levels by 50% and LDL-C levels by ≤20%, and increase HDL-C levels by ≤20%. These effects are closely related to baseline lipid levels.^287^ Meta-analysis showed that fibrates-treated patients with high TG and low HDL-C had a decrease of major cardiovascular events without reduced CVD or total mortality.^288,289^ Recently, a novel fibrate, pemafibrate, was reported to significantly reduce TG-rich lipoproteins, such as chylomicrons and VLDL.^290^ In addition, fibrates are well tolerated with common adverse effects of myopathy, elevated liver enzymes, and cholelithiasis.^291^ Overall, the CVD benefit of fibrates requires further confirmation.

##### Lipoprotein apheresis

Lipoprotein apheresis (LA) is a non-drug lipoprotein-lowering therapy commonly used in patients with HoFH, heterozygous FH and other forms of hypercholesterolemia or CVD.^292^ Although highly effective, LA is time-consuming and expensive, and has long been the last resort for treating uncontrolled dyslipidemia.^293^

#### New targets for cholesterol-lowering therapy

In addition to the classical targets for drug mentioned above, some new targets for cholesterol lowering are also being investigated, which we will elaborate below (Table 2).

**Table 2:** New targets and their clinical advances

New targets	Function	Clinical advances
APOC3	APOC3 is mainly found in VLDL and chylomicron, and can stimulate liver to synthesize and secrete VLDL	Volanesorsen was approved for use in patients with Familial chylomicemia syndrome in Europe in May 2019; Olezarsen is currently in phase 3 clinical trials; ARO-APOC3 is currently in phase 3 clinical trials
Lipoprotein (a)	Lp(a) is a special form of LDL particle containing 35-46% CE and 6-9% cholesterol	Phase 2 trial of pelacarsen demonstrated significant Lp(a) lowering capacity; Olpasiran is currently in phase 3 clinical trials
LXRs	Activation of LXRs increases the rate of RCT by increasing ABCG1 and ABCA1 expression in macrophages but also up-regulates liver SREBP1c, leading to hepatic steatosis and hypertriglyceridemia	None
CETP	CETP promotes the transfer of cholesterol esters from HDL to LDL particles	Most CETP inhibitors have been discontinued for a variety of reasons. The latest CETP inhibitor, obicetrapib, is currently in phase 3 clinical trials
LOX-1	LOX-1 is a scavenger receptor for oxLDL and affect the uptake of oxLDL by cells	None
SR-BI	Liver SR-BI regulates RCT by taking up HDL-C and transporting cholesterol to bile	None
LCAT	LCAT is an enzyme in plasma that esterifies cholesterol	MEDI6012 was abandoned in phase 2 for safety or efficacy reasons
MiR-33 and miR-122	miR-33 inhibits expression of the genes involved in cholesterol efflux and HDL synthesis; miR-122 is the most abundant hepatic miRNA and its levels are positively correlated to human plasma cholesterol levels	None
Prekallikrein	Prekallikrein is identified as a binding protein of LDLR	None

##### APOC3

Apolipoprotein C3 (APOC3) is an apolipoprotein encoded by the gene *APOC3* and mainly found in VLDL and chylomicron.^294,295^ APOC3 can stimulate liver to synthesize and secrete VLDL.^296^ It also reduces liver clearance of TG-rich lipoproteins by regulating LDLR/LDLR-related protein 1 (LRP1) pathway.^297^ Epidemiological studies show that plasma APOC3 levels can be used to predict CVD risk and mortality.^298–301^ It has been reported that carriers of rare heterozygous deletion mutations in APOC3 have lower TG, enhanced HDL-C, little change in LDL-C and lower cardiovascular risk.^302,303^

Volanesorsen is a second-generation of antisense oligonucleotide (ASO) targeting *APOC3* mRNA in hepatocytes to decrease APOC3 expression, thereby significantly reducing plasma TG levels.^304^ APO-CIII-L_Rx_ is a next-generation of N-acetylgalactosamine-conjugated ASO targeting APOC3. In a double-blind, placebo-controlled, dose-escalation phase 1/2a study, multiple injections of 30 mg/week APO-CIII-L_Rx_ reduced APOC3, TG, VLDL, TC, LDL-C by ~80%, 70%, 70%, 15%, and 15%, respectively, and increased HDL-C by about 70%.^305^

Based on these studies, it is suggested that inhibition of APOC3 also has cholesterol lowering potential, although the mechanism remains unclear.

##### Lipoprotein (a) [Lp(a)]

Lp(a) is a special form of LDL particle encoded by *LPA*, to which part of Apo(a) is covalently bound to ApoB. Lp(a) contains 35–46% CE and 6–9% cholesterol.^306,307^ The concentration of Lp(a) is mainly determined by genes and varies greatly among individuals.^308^ In the past, multiple studies have demonstrated that Lp(a) is another risk factor for ASCVD.^309–312^

The in vitro and animal studies suggest that Lp(a) is important in the progression of atherosclerosis by influencing formation of foam cells, VSMC proliferation, and plaque inflammation and instability.^313,314^ But in individuals with high Lp(a) levels, the content of atherogenic cholesterol carried by LDL is generally much higher than carried by Lp(a).^315^ However, vascular dynamics studies have shown that Lp(a) accumulates preferentially in the vascular wall, which may indicate that the cholesterol carried by Lp(a) has more atherogenic potential than LDL-C.^316^

So far, there is no approved pharmacological approaches to reduce Lp(a) to the level which can benefit ASCVD.^317^ However, niacin, mipomersen and PCSK9 inhibitors show a certain effect on lowering Lp(a), although these effects may not translate into substantial clinical benefits.^318–320^ The recently concluded phase 2 trial of pelacarsen demonstrated significant Lp(a) lowering capacity. Pelacarsen is a hepatocyte-directed ASO targeting liver *LPA* mRNA, and can significantly reduce Lp(a) production.^321^ In addition, another siRNA drug, olpasiran, also shows a strong Lp(a)-lowering effect.^322^ Taken together, existing evidence suggests that Lp(a) is a potential target to treat ASCVD, and drugs targeting it are under intense development.

##### LXRs

The oxysterol-activated receptors, LXRα and LXRβ, are members of the nuclear transcription receptor family. LXRs play important roles in RCT through multiple mechanisms. In different mouse models, in vivo activation of LXRs increases the rate of RCT by increasing ABCG1 and ABCA1 expression in macrophages.^323–325^ In addition, activation of LXRs also has a significant anti-inflammatory effect.^326^ Therefore, targeting LXRs is a potential anti-atherosclerotic strategy. T0901317 and GW3965 are synthetic agonizts of LXRs that could significantly reduce plaque formation in atherosclerotic mice.^327,328^ However, activation of LXRs also up-regulates liver SREBP1c, leading to hepatic steatosis and hypertriglyceridemia, which limits clinical application of LXR agonizts.^329^ For this reason, some specific targeted agonizts have been developed. GW6340 is a gut-specific LXR agonist which promotes macrophage RCT but has no effect on TG levels in plasma.^330^ Furthermore, IMB-808 significantly activates cholesterol efflux from RAW264.7 and THP-1-derived macrophages while has little effect on expression of lipogenic genes in HepG2 cells.^331^

In order to avoid the side effects of LXRs agonizts, some methods of drug combination or targeted therapy have also been developed. We demonstrated that T0901317 in combination with a MEK1/2 inhibitor, U0126, inhibited atherosclerosis and blocked T0901317-induced hypertriglyceridemia.^332^ We also reported that the combined treatment of metformin and T0901317 not only blocked T0901317-induced hypertriglyceridemia, but also enhanced the atherosclerosis-inhibiting effect of T0901317 by selectively activating LXRβ but not LXRα.^333^ In view of the good targeting of nanomaterials, the side effects of liver can be avoided by using nano-carriers to deliver LXR agonizts. Last year, we reported a nanofibrous hydrogel, encapsulated T0901317 by the small peptide D-Nap-GFFY, selectively targeted macrophages but not hepatocytes. Thus, the hydrogel-encapsulated T0901317 inhibited the development of atherosclerosis without increasing TG levels.^334^ Although LXR agonizts have been shown the potential to slow atherosclerosis progression in animal models, they are still a long way from clinical use.

##### CETP

CETP inhibitors can reduce LDL-C and increase HDL-C levels by inhibiting the transfer of cholesterol esters from HDL to LDL particles.^188^ It has been reported that CETP activity is significantly elevated in patients with metabolic disorders and a high cardiovascular risk, indicating CETP can be a potential indicator of cardiovascular risk.^335^ In vivo experiments show that elimination of CETP activity inhibits cholesterol diet-induced atherosclerosis in rabbits.^336^ These results provide a basis for the potential of CETP inhibitors to improve blood lipids and reduce ASCVD risk.

CETP inhibitors to date include torcetrapib, dalcetrapib, evacetrapib, anacetrapib and obicetrapib. Since CETP is not existing in mice, most translational studies of CETP inhibitors are performed in ApoE3*CETP Leiden mice. Unfortunately, the first CETP inhibitor, torcetrapib, has been observed to increase the incidence of cardiovascular events and overall mortality, although it increased HDL-C while decreased LDL-C.^337^ When used in treatment of patients with acute coronary syndrome, dalcetrapib had no effect on reduction of the recurrent cardiovascular events, therefore, use of dalcetrapib was discontinued early.^338^ Similarly, evacetrapib adversely affected the cardiovascular outcomes in patients who had high risk of vascular disease.^339^ On the other hand, anacetrapib significantly improved lipids and reduced the incidence of major coronary events in patients with a good tolerance.^340^ However, anacetrapib was also discontinued due to its long half-life. A 12-week monotherapy trial of obicetrapib, the latest CETP inhibitor, showed a 45.3% reduction in LDL-C compared to placebo.^341^ Current studies are evaluating obicetrapib in patients who are intolerant of statins in a phase 3 study.

##### LOX-1

LOX-1 is a scavenger receptor for oxLDL and plays an important role in oxLDL uptake by cells.^342^ In atherosclerotic plaques and surrounding tissues, LOX-1 is highly expressed. It promotes uptake of oxLDL by ECs, VSMCs, monocytes and macrophages, resulting in foam cell formation.^342^ At the same time, some studies have shown that LOX-1 deletion significantly reduces oxidative stress, nitric oxide degradation and inflammatory responses, reducing the progression of atherosclerosis.^343,344^ Therefore, it is suggested that LOX-1 promotes the atherosclerosis progression. Contradictorily, liver overexpression of LOX-1 promoted oxLDL uptake, decreased plasma oxLDL, and inhibited the progression of atherosclerosis in ApoE-deficient mice.^345^ Hence, LOX-1 is also a key regulator in the mechanisms of atherosclerotic plaque formation, progression and instability which may need further investigation.

Currently, some natural products, such as Tanshinone II-A, curcumin and Gingko biloba extract, have been shown to prevent atherosclerosis through LOX-1 inhibition.^346–348^ The LOX-1 molecule consists of a hydrophobic channel that is the primary binding site for the phospholipid moiety of oxLDL.^349^ Chemically synthesized small molecules targeting this channel can effectively reduce oxLDL uptake in vitro.^350^ In addition to chemically synthesized inhibitors, many monoclonal antibodies are available to block LOX-1 activity. However, these antibodies are currently limited to cell and animal experiments because LOX-1 molecule contains a highly conserved C-type lectin-like domain in mammals, making it challenging to develop human LOX-1 antibodies.^351^ At present, the research of chimeric LOX-1 antibody is still in progress.

##### SR-BI

SR-BI is a member of the scavenger receptor family. Liver SR-BI regulates RCT by taking up HDL-C and transporting cholesterol to bile. Liver SR-BI regulates HDL composition, mediates cholesterol efflux, and reduces inflammation and oxidation through selective uptake of HDL lipids. In macrophages and ECs, SR-BI is important in inhibiting atherosclerosis and reducing foam cell formation by regulating cholesterol transport.^352^ Therefore, SR-BI is a potential multifunctional target for inhibiting atherosclerosis.

The current study has identified the protective role of SR-BI in mice with atherosclerosis. Genomic analysis reveals increased risk of CVD in loss-of-function carriers of scavenger receptor class B member 1 (*SCARB1*) variant, which encodes SR-BI, suggesting the protective role of SR-BI in atherosclerosis.^353^ Given the recent appreciation of endothelial SR-BI in LDL transcytosis, SR-BI targeted therapies need to be assessed with caution.^354^ At present, the mechanism by which SR-BI works in human body is still unclear, so exploring its detailed mechanism is crucial for the development of new treatments for atherosclerosis.

##### LCAT

LCAT is the only enzyme in plasma that esterifies cholesterol, and its activity is a major determinant of HDL-C levels.^355^ LCAT plays a central role in HDL metabolism and RCT, so it is generally considered to be anti-atherosclerotic. However, studies in humans and animals obtained different results, so whether its activity can improve the function of HDL is controversial.^356,357^ This may be related to the levels of LDL-C, the presence or absence of CETP and SR-BI, and the degree of overexpression of LCAT.^356^

AlphaCore Pharmaceuticals developed the original recombinant human LCAT (rhLCAT) for clinical testing. In a phase 1 clinical trial, this early rhLCAT formulation, ACP501, increased plasma HDL-C by 50% and promoted cholesterol efflux without serious adverse reactions.^358^ Since then, a new formulation of rhLCAT, MEDI6012, has been developed, which can raise plasma HDL-C in patients with atherosclerosis by injection three times a week.^359^ However, it was abandoned in phase 2 for safety or efficacy reasons. Compound A is the first identified small molecular activator of LCAT that can covalently bind to residue C31 of LCAT, and has been shown to increase LCAT activity in vitro with unclear function on atherosclerosis.^360,361^

In addition, another class of activators bind LCAT in a non-covalent and reversible manner. Previous studies have shown that such activators stabilize the open, active conformation of the enzyme, thereby facilitating lipid transport to the active site.^362^ DS-8190a is an orally bioavailable and novel small-molecular LCAT activator that can directly interact with human LCAT. It inhibited atherosclerosis in mice expressing human LCAT, which was associated with enhanced the RCT process. Oral administration of DS-8190A also stimulated RCT process in primate cynomolgus monkeys.^363^ These studies suggest that LCAT activation may help to reduce residual risk of ASCVD.

##### MiR-33 and miR-122

MicroRNAs (miRNAs) belong to a family of endogenous noncoding RNAs that can regulate gene expression post-transcriptionally. By binding to the 3′-untranslated region (3′UTR) of target genes, miRNAs promote translational repression or mRNA degradation.^364^ Recent studies have shown that miRNAs are involved in cholesterol uptake, synthesis, and efflux, and are expected to be potential targets for regulating cholesterol metabolism.^365–367^

miRNA-33 (miR-33) is composed of miR-33a and miR-33b, located in the *SREBP2* and *SREBP1* gene introns, respectively, and co-expressed under different stimulation conditions.^368,369^ miR-33 inhibits expression of the genes involved in cholesterol efflux and HDL synthesis, such as *ABCA1* and *ABCG1*.^370^ Studies have shown that inhibition of miR-33 induces hepatic ABCA1 expression, thereby increasing plasma HDL-C levels, and the inhibition also promotes RCT in macrophages and regression of atherosclerosis.^371,372^ In addition, some studies have investigated the role of miR-33 on VLDL/LDL metabolism. It has been reported that global knockout of miR-33 in mice increases plasma LDL-C/VLDL-C levels.^373^ However, mice may experience these effects due to their genetic background. The levels of VLDL-C and VLDL-TG were increased in LDLR deficient mice but not ApoE deficient mice fed Western diet after miR-33 knockout, which may be due to a high basal level of VLDL in ApoE deficient mice.^374,375^ Based on the existing studies, although inhibition of miR-33 can effectively improve cholesterol efflux and HDL synthesis, its side effects remain to be clarified.

miRNA-122 (miR-122) is the most abundant hepatic miRNA. Its levels are positively correlated to human plasma cholesterol levels, suggesting that miR-122 can be involved in regulation of cholesterol metabolism.^376^ miR-122 inhibitors have been reported to reduce plasma TC levels in mice and non-human primates.^377–379^ However, miR-122 deletion is accompanied by significant hepatic steatosis, so the safety of miR-122 treatment remains to be investigated.^380^ Moreover, to designate miR-122 as a potential therapeutic target for regulating cholesterol metabolism, the further elucidation on its physiological role is required.

##### Prekallikrein

Recently, the coagulation factor PK [encoded by the kallikrein B1 (*KLKB1*) gene] was identified as a binding protein of LDLR.^140^ In this study, it was found that PK binds to LDLR and causes LDLR lysosomal degradation, while plasma PK concentrations in humans are positively correlated to LDL-C levels. Loss of KLKB1 increases hepatic LDLR and reduces FC, attenuating atherosclerosis progression in multiple rodent models. In addition, the use of anti-competitive neutralizing antibodies can also reduce plasma lipids by up-regulating liver LDLR. This study suggests that PK may represent a potential treatment target for ASCVD.

### Benefits of improving cholesterol homeostasis in other diseases

In addition to ASCVD, cholesterol metabolic disorders are also involved in the pathogenesis of other diseases and cholesterol lowering can ameliorate them. Interestingly, improving cholesterol homeostasis may be beneficial to several diseases even the role of cholesterol in these diseases remains unclear.

#### NAFLD

NAFLD is a chronic liver disease caused by excessive lipid deposition in liver cells without significant alcohol intake.^381^ NAFLD includes nonalcoholic fatty liver (NAFL) and nonalcoholic steatohepatitis (NASH).^382^ The accumulation of FC in the liver is also relevant to the pathogenesis of NAFLD.^383,384^ Epidemiological studies have found that intake of excess dietary cholesterol significantly increases the risk of NAFL and NASH.^385,386^ A study of lipidomic analysis of liver biopsies from patients with NAFLD showed that hepatic FC level was positively correlated to the severity of liver histopathology.^382^ Animal studies also showed that exogenous induction of FC accumulation in the liver can promote the progression of NAFL to NASH.^387,388^

In NAFLD, hepatic cholesterol homeostasis is imbalanced, resulting in elevated levels of hepatic cholesterol.^389^ This dysregulation may involve multiple metabolic pathways, including activation of cholesterol biosynthetic pathway (elevated expression and activity of SREBP2 and HMGCR), and cholesterol de-esterification (enhanced hydrolysis of CE to FC by hepatic neutral CE hydrolase), and reduced cholesterol export and BA synthesis (reduced expression of ABCG8 and CYP7A1).^70,384,390,391^ However, the contributions of these pathways to NAFLD need to be further explored.

The exact mechanism of excess cholesterol toxicity in NAFLD remains incompletely described. Excess cholesterol accumulation in hepatocytes stimulates the formation of cholesterol crystals.^392^ The presence of cholesterol crystals in hepatocytes activates NLRP3 inflammation, ultimately leads to hepatocyte death. Küpffer cells (KCs) aggregate around necrotic hepatocytes and trigger the formation of “crown-like structures”. Subsequently, KCs process these cholesterol crystals released from the dead hepatocytes and transform into foam cells.^383,392^ Meanwhile, cholesterol crystals-induced activation of KCs triggers the activation of hepatic stellate cells (HSCs) by releasing inflammatory cytokines and transforming growth factor β, further accelerating the progression of NASH to fibrosis.^393^ Furthermore, transcriptional coactivator with PDZ-binding motif (TAZ) is a transcriptional regulator that promotes NASH fibrosis and its expression is significantly increased in the NASH process.^394–396^ Wang et al. firstly demonstrated that cholesterol prevents TAZ proteasomal degradation *via* the soluble adenylate cyclase-protein kinase A-inositol trisphosphate receptor-calcium-RhoA pathway.^397^ This provides a new mechanism for the importance of hepatocyte cholesterol in the development of NASH. In summary, the cholesterol accumulation in hepatocytes and hepatic non-parenchymal cells accelerates the pathological process of NAFLD.

Clinical data show that statin treatment in patients with NAFLD reduces intrahepatic cholesterol levels.^398–400^ Interestingly, the effect of ezetimibe on NAFLD in clinical trials is controversial. Several clinical studies suggest that ezetimibe may be beneficial for NAFLD.^401,402^ However, a randomized, double-blind, placebo-controlled trial showed that ezetimibe had no significant effect on liver histology in NASH patients,^403^ indicating more studies are needed to address the effect of ezetimibe. In addition to classic cholesterol-lowering drugs, other interventions to lower cholesterol may also be beneficial for NAFLD. Lanifibranor is a pan-PPAR agonist. In a recent phase 2b clinical study, lanifibranor not only showed good tolerability but also significantly improved liver fibrosis in NASH patients.^404^ Lanifibranor improved NASH may be partially related to lowering cholesterol. Yang et al. found that knockout of E3 ligase SH3 domain-containing ring finger 2 (*SH3RF2*) in hepatocytes resulted in accumulation of acetyl-CoA, which directly promoted cholesterol synthesis and aggravated the development of NAFLD.^405^ Furthermore, miRNAs are key factors in regulating hepatic cholesterol synthesis.^406^ Targeting *SH3RF2* or miRNAs may be a new approach to alleviate NAFLD by lowering cholesterol.

#### Obesity

Obesity is the manifestation of metabolic syndrome in the adipose tissue, which is associated with various chronic diseases, particularly CVD, diabetes, and certain types of cancers.^407–409^ Changes in diet composition are one of the main reasons for the increasing trend of obesity. Chung et al. demonstrated that high dietary consumption of cholesterol was sufficient to induce an increase in visceral adipose cholesterol content and promote inflammation with adipose tissue in monkeys.^410^ In addition, the genome-wide association studies have found the significant association between NPC1 and obesity.^411^ This may provide a new explanation for familial obesity.

Adipose tissue plays a central role in energy metabolism and adaptation to the nutritional environment, and about 25% of the person’s cholesterol is stored in adipose tissues.^412^ In obesity, cholesterol imbalance triggers inflammation in adipocytes and fat-resident immune cells, thus disrupting metabolic homeostasis.^413^ In the initial stages of obesity, white adipose tissue exhibits physiological expansion and releases acute pro-inflammatory factors in order to store more energy.^414^ Therefore, this initial pro-inflammatory response may be only physiologically adaptive. However, when cholesterol crystals accumulate in adipocytes and immune cells, it activates NLRP3 inflammasome, leading to increased inflammation.^415^ Meanwhile, local inflammation in adipose tissue may directly affect brown adipocyte thermogenesis and beige adipocyte recruitment, which also hinders thermogenesis.^414^ Taken together, excessive accumulation of cholesterol in adipose tissues causes inflammation and adipocyte dysfunction. Therefore, cholesterol-lowering therapies may be beneficial for obesity.

Triiodothyronine (T3) is the biologically active form of thyroid hormone. Grover et al. demonstrated that T3 regulates cholesterol metabolism *via* acting thyroid hormone receptor β signaling.^416^ Both clinical and animal studies have shown that T3 treatment increased the rate of cholesterol metabolism.^416,417^ However, the pharmacological benefits of T3 are limited by its side effects, particularly on heart rate. A novel strategy preferentially delivers T3 to the liver, thus mitigating its side effects.^418^ Some new cholesterol-lowering targets may also be beneficial for obesity. Berbe´e et al. demonstrated that β3-adrenergic receptor-stimulated activation of brown adipose tissue reduces obesity by decreasing plasma cholesterol levels.^419^ The selective thyroid hormone receptor modulator GC-1 has been shown to have better cholesterol-lowering efficacy than atorvastatin in animal studies.^420^ These observations deserve further studies and hopefully offer new perspective for the treatment of lipid disorders and obesity. Interestingly, diet and lifestyle changes can also lower cholesterol. In a clinical trial with 82 healthy overweight and obese subjects, an isocaloric Mediterranean diet intervention was found to lower plasma cholesterol and alter the microbiome and metabolome.^421^ Moreover, dietary and exercise interventions produced better outcomes for obese children.^422^ Solving the obesity problem is a daunting challenge that seems to inevitably require multiple interventions. The development of drugs to treat obesity has been underway for more than a century and is continuing.^423^ Consequently, for obese patients, lowering cholesterol may need to be used in combination with other interventions.

#### Diabetes

The relationship between TG and diabetes has been proposed at a fairly early stage.^424–426^ However, the role of cholesterol has been underrecognized. The specific cholesterol homeostasis in pancreatic β cells plays a key role in insulin secretion. In 2007, two studies demonstrated that excess cholesterol inhibits insulin secretion from β cells. Brunham et al. reported that mice with specific knockout of ABCA1 in β cells had increased cholesterol levels and impaired glucose-stimulated insulin secretion.^427^ Likewise, Hao et al. proved that accumulation of cholesterol in β cells influenced the translocation and activation of glucokinase, further inhibiting insulin secretion.^428^ Subsequently, Vergeer et al. confirmed that carriers of loss-of-function mutant ABCA1 have pancreatic β-cell dysfunction.^429^ The final step in insulin secretion is the fusion of insulin granules with plasma membrane and then secreted outside the cell through exocytosis. Xu et al. found that excess cholesterol can reduce insulin exocytosis through a dynamic-dependent process activated by phosphatidylinositol 4,5-bisphosphate.^430^ Meanwhile, cholesterol accumulation also induces apoptosis of pancreatic β cells by enhancing mitochondrial bioenergetic damage, inflammation, oxidative stress and ER stress.^431–433^ In addition, imbalanced cholesterol homeostasis in β cells increases obesity, reduces skeletal muscle mass and causes systemic inflammation.^434^ This may provide a new explanation for the link between diabetes and obesity.

Given the harmful effects of cholesterol on β-cell function, cholesterol-lowering therapies may be therapeutically beneficial. In a randomized, double-blinded study, subjects taking a CETP inhibitor significantly increased postprandial insulin secretion.^435^ This may be due to increased cholesterol efflux from pancreatic β cells.^435^ Surprisingly, there is growing evidence showing that statin therapy could increase the risk of diabetes in a dose-dependent manner.^436–438^ A recent animal study explains that atorvastatin impairs β-cell function by modulating small G protein, which subsequently dysregulating islet mTOR signaling and reducing functional β-cell mass.^439^ Therefore, statins may need to be combined with other drugs for a better use in diabetic patients with hypercholesterolemia. Interestingly, ezetimibe promotes insulin secretion and protects β-cell function in diabetic mice.^440^ Exploring the specific mechanism of ezetimibe to promote insulin secretion will be an interesting future investigation. Moreover, miR-33a and miR-145 can downregulate ABCA1, leading to cholesterol accumulation and reduction of insulin secretion.^441,442^ Thus, targeting microRNAs or other epigenetic mechanisms may offer a promising therapeutic strategy for diabetes and its complications.

#### Neurodegenerative diseases

The brain is the cholesterol-rich organ in the body, accounting for approximately 20% of the body’s cholesterol.^443^ Cholesterol homeostasis in the brain must be accurately controlled to ensure the brain to work properly.^444^ Imbalance of cholesterol homeostasis in the brain is involved in the development of neurodegenerative diseases including Alzheimer’s disease (AD), Parkinson’s disease (PD), and Huntington’s disease (HD).

Several reviews have linked cholesterol to the pathophysiology of AD, revealing the importance of cholesterol homeostasis in AD.^445–447^ In an early clinical study, FH was shown to be an early risk factor for AD.^448^ Plasma cholesterol can be oxidized to 27-hydroxycholesterol, which is able to cross the blood-brain barrier (BBB) and reach the central nervous system (CNS).^449^ This establishes a critical link between FH and increased brain cholesterol. Xiong et al. stained brain sections from AD patients and found that cholesterol levels increased with disease progression.^450^ A recent animal study has shown that a high-cholesterol diet disrupts BBB and impairs cognitive function.^448^ Cutler et al. found that oxidative stress induced disturbances in cholesterol metabolism, leading to enrichment of cholesterol in neurons, which exacerbates the process of AD.^451^ It is necessary to note that lipoproteins can’t cross the intact BBB.^444^ The accumulation of cholesterol in the brain may be due to a disruption of BBB or a disturbance in the brain’s own cholesterol metabolism. However, the exact mechanism needs to be further explored.

Amyloid protein is cleaved to β-amyloid (Aβ) by β and γ-secretase. Aβ aggregation is the predominant pathological marker of AD.^445^ Sparks et al. identified the effect of cholesterol on Aβ accumulation in 1994.^452^ They found that feeding a cholesterol-rich diet to rabbits for eight weeks led to accumulation of intracellular Aβ in neurons in the hippocampal region. Many subsequent experiments have also demonstrated that cholesterol promotes Aβ accumulation. A key reason for the sensitivity of Aβ to cholesterol is that the activity of β and γ secretase is positively correlated to cholesterol levels.^446,453^ Furthermore, cholesterol not only promotes Aβ secretion, but also impairs autophagy-mediated clearance of Aβ. Pathological accumulation of phosphorylated Tau (pTau) is another major biochemical marker of AD. Meanwhile, hyperphosphorylation of tau is accompanied with formation of neurofibrillary tangles (NFTs).^454,455^ Imbalance in cholesterol homeostasis also increases pTau. A case-control study found a significant tau deposition in the brains of Niemann-Pick type C patients.^456^ CE are the major storage form of excess cholesterol, and Kant et al. found that CE inhibited pTau degradation by inhibiting proteasome activity.^457^ Conversely, Fan et al. demonstrated that cholesterol deficiency also leads to tau hyperphosphorylation,^458^ indicating the exact mechanism of cholesterol effects on p-Tau remains to be further explored.

PD is the second most common progressive neurodegenerative disease after AD, and its pathological features include the loss of dopaminergic neurons and the formation of Lewy bodies from the accumulation of α-synuclein.^459^ Increasing evidence suggests that cholesterol metabolism may also play a role in the pathogenesis of PD. However, the role of TC in PD is controversial. Some clinical studies found no difference in TC levels between PD patients and healthy controls.^460,461^ In contrast, other prospective studies even found that high levels of TC were associated with a lower risk of PD.^462,463^ This may be due to the fact that cholesterol levels decrease with age, and PD usually occurs more often in older age. As reported by Hu et al., the high TC levels increases the risk of PD in individuals aged 25-54 years, but this association is not significant after 55 years.^464^ Thus, high TC levels in young and middle-aged individuals may promote PD development, which has been demonstrated in animal models with high-fat diets.^465,466^

In spite of the unclear role of cholesterol in PD pathogenesis, several possible hypotheses have been proposed. Bar-On et al. treated B103 cells with cholesterol and found more α-synuclein aggregates while statin can reduce the aggregation.^467^ The subsequent studies found that α-synuclein has a similar structure to apolipoproteins.^468,469^ Thus, there is an interaction between cholesterol and α-synuclein. Fantini et al. found that cholesterol promotes α-synuclein insertion into lipid rafts through a virus-like fusion mechanism.^469^ Hsiao et al. found that α-synuclein promotes cholesterol efflux in SH-SY5Y cells.^470^ However, the relationship between cholesterol and α-synuclein remains to be further explored.

HD is an autosomal dominant neurodegenerative disorder caused by an abnormal expansion of the CAG trinucleotide repeat of the Huntington (*HTT*) gene.^471^ Cholesterol homeostasis is altered in HD, which may be an effective disease-modifying strategy in the future.^472^ An early investigation showed no significant changes in plasma cholesterol concentrations in HD patients.^473^ However, another study found reduced mRNA levels of HMGCR, and 7-dehydrocholesterol reductase in postmortem tissues of HD patients.^474^ Subsequently, Leoni et al. reported reduced blood cholesterol levels in HD patients.^475^ Similarly, reduced brain cholesterol levels were also found in a variety of HD animal models.^476–478^

Interestingly, reduced cholesterol level is more likely a phenomenon in the process of HD pathogenesis. There is evidence showing that mutant Huntington (m*HTT*) interferes with SREBP2 activation, leading to reduced expression of HMGCR and cholesterol synthesis.^479^ Brain-derived neurotrophic factor (BDNF) can also stimulate cholesterol synthesis.^480^ Normal *HTT* promotes vesicular transport of BDNF vesicles along microtubules.^481^ However, this process is inhibited by m*HTT*, resulting in decreased BDNF levels in the striatum, which may be another pathway leading to reduced cholesterol synthesis.^478^ In contrast, cholesterol accumulates in mHTT­expressing neurons despite the downregulation of cholesterol synthesis.^482^ Daniel et al. found that *mHTT*­expressing neurons show elevated levels of the lipid raft marker ganglioside GM1, suggesting that cholesterol accumulation is associated with an increase in lipid rafts.^483^ The present evidence suggests that reduced cholesterol synthesis and cholesterol accumulation in neurons are the main manifestations of imbalanced cholesterol homeostasis in HD. Determining which aspect of cholesterol dysregulation primarily affects the pathological process of HD will be a major challenge in the future.

Based on the reports above, modulation of cholesterol homeostasis could be a potential therapeutic target for neurodegenerative diseases. Lipophilic statins can cross the BBB and have the potential to modulate cholesterol homeostasis in the brain.^484^ Several preclinical trials have shown multiple potential benefits of statins in neurodegenerative diseases.^484–487^ Although the protective effects of statins in preclinical trials are consistent, the results of clinical trials remain controversial. Epidemiological studies have shown a 70% reduction in incidence of AD in subjects taking statins.^488^ Treatment of subjects with statin at doses used in the clinical management of hypercholesterolemia resulted in a nearly 40% reduction in Aβ production in human plasma.^489^ Li et al. reported that NFT burden was significantly reduced in subjects who had taken statins by brain autopsy.^490^ By contrast, a cohort study that included 2798 individuals found that statin treatment was not associated with the risk of AD.^491^ Similarly, most observational studies have shown that the use of statins reduces the risk of PD,^492–494^ whereas some clinical trials have found that statins have no effect on PD or even increase the odds of PD.^495,496^ However, no clinical trials have been conducted to evaluate the role of statins in HD to date. Due to the specificity of cholesterol homeostasis in HD, the benefit of statins in HD may be through anti-inflammation, anti-oxidative stress, and neuroprotection, rather than the ability to regulate cholesterol metabolism. Therefore, well-designed preclinical trials are needed to prove the effects of statins on HD. Other cholesterol-lowering drugs have also shown protection against neurodegenerative diseases in preclinical animal models. Efavirenz reduces p-Tau in a dose-dependent manner by decreasing CE production.^457^ BM15.766, a specific inhibitor of cholesterol synthesis, showed inhibition of Aβ in transgenic AD mice model.^497^ In addition, LXRs are major regulators of cholesterol homeostasis and inflammation in the CNS.^498^ LXRs agonizts were shown to have alleviating effect in neurodegenerative diseases in preclinical trials.^499–501^ β-Cyclodextrin and its derivatives also have a beneficial effect on the neurodegenerative diseases as drugs or drug carriers.^502,503^ The pathogenesis of neurodegenerative diseases is mediated by a variety of factors, and cholesterol disorders may intricately aggravate the disease process. Considering the importance of cholesterol for the brain cell membrane integrity, cholesterol-lowering drugs should be used precisely with tailored needs. In other words, they are recommended for patients of neurodegenerative diseases with a relatively high cholesterol background.

#### Cancers

Cholesterol is an essential neutral lipid which is necessary for membrane integrity and fluidity.^504^ The increasing evidence demonstrate that tumor cells need an increased supply of cholesterol and can accumulate it.^505–507^ It has been reported that during cancer progression, cholesterol influx and synthesis is increased and cholesterol efflux is decreased.^508^ Aberrant activation of SREBPs is the main cause of increased tumor cholesterol synthesis. For example, in hepatocellular carcinoma, the sustained activation of protein kinase B (PKB) phosphorylates phosphoenolpyruvate carboxykinase 1, which in turn activates SREBPs and promotes tumor growth.^509^ The alteration of the extracellular microenvironment of tumor cells also leads to activation of SREBPs. In breast cancer models, hypoxia induces PKB phosphorylation, which in turn activates hypoxia-inducible factor 1 and subsequently upregulates expression of SREBPs.^510^ In addition, increased inflammatory factors, lower pH and excess glucose in the microenvironment can also activate SREBPs.^510,511^ LXR promotes expression of cholesterol efflux proteins, ABCA1, ABCG1 and ABCG5, to reduce intracellular cholesterol concentrations. However, LXR is inhibited in tumors, which contributes to cholesterol accumulation in cancer cells.^512,513^ Interestingly, CE levels were also significantly increased in tumors.^513,514^ ACAT involves in synthesis of CE, which has been shown to be associated with a variety of tumors.^513,515^ A latest study found that loss of *P53* increased ubiquitin specific peptidase 19, which in turn stabilized ACAT1 and led to CE accumulation.^516^ This study provides an important mechanism indicating the involvement of CE in hepatocellular carcinogenesis.

Similar to tumor cells, activation of cholesterol synthesis pathway is necessary to maintain T cell function. However, excessive cholesterol in the tumor microenvironment leads to ER stress in CD8^+^ T cells. Furthermore, the ER stress sensor X-box-binding protein 1 is activated to regulate transcription of programmed death 1 and natural killer cell receptor 2B4, which ultimately leads to T cell exhaustion.^517^ It can be seen that the effect of increased extrinsic supply of cholesterol on T cells seems to be negative in the situation where tumor cells have a greater capacity to absorb cholesterol. In another study, ovarian cancer cells promoted tumor-associated macrophage (TAM) cholesterol efflux by secreting hyaluronic acid, which induced TAM conversion from M1 to M2 type and promoted tumor growth.^518^

Statins have been shown to have good inhibitory effects on estrogen receptor-negative breast cancer, multiple myeloma, prostate cancer and some other specific tumors.^519–521^ However, in several phase 3 clinical trail studies, treatment of 40 mg/day pravastatin or simvastatin to patients with small cell lung cancer, metastatic colorectal cancer, advanced hepatocellular carcinoma, or advanced gastric cancer had no additional benefit.^522–525^ Therefore, a precision medicine approach is necessary if statins are to be incorporated into the treatment of cancer patients. Avacizimibe, a potent inhibitor of ACAT1, has been shown to affect the survival and proliferation of tumor cells in several preclinical studies.^526–528^ The clinical application of Avacizimibe in anti-tumor needs to be further explored. In addition, drugs targeting the absorption and efflux of cholesterol have been tried for cancer treatment. LXR agonist, T0901317, suppressed the development of prostate cancer by upregulating ABCA1 and ABCG1 expression.^529^ Ezetimibe significantly inhibited the growth of prostate and liver cancers.^530,531^ Yuan et al. found that the tumor microenvironment could inhibit LDLR expression in CD8^+^ T cells *via* activating PCSK9, which suppressed the antitumor activity of CD8^+^ T cells.^532^ Therefore, PCSK9 may be a novel target for tumor immunotherapy. The anti-tumor effects of PCSK9 inhibitors need to be further explored. In summary, drugs targeting cholesterol metabolic pathways have been demonstrated in many cancers. Considering the complexity of cancer metabolism, there are still many open questions that need to be addressed. For example, at what stage of tumorigenesis do these drugs act specifically, such as tumor metastasis? Do statins affect the function of circulating tumor cells? How do statins affect tumor cell metabolism in tumor microenvironment?

#### Osteoporosis

Osteoporosis most commonly occurs to postmenopausal women caused by impaired bone formation and/or excessive bone resorption. Bone mineral density (BMD) is considered as the key standard for determining osteoporosis.^533^ Vitamin D, one of the important metabolites of cholesterol, induces synthesis of calcium-binding proteins to promote Ca^2+^ absorption and enhances BMD.^534^ Interestingly, epidemiological evidence indicates that high serum cholesterol levels represent a risk factor for osteoporosis.^535–538^ Also, this phenomenon has been confirmed in several animal experiments.^539–541^

Previous studies have given several possible explanations for why cholesterol increases the risk of osteoporosis. Cutillas-Marco et al. found that vitamin D levels were negatively associated with TC and LDL-C levels in a population-based survey.^542^ This may be the most important cause of osteoporosis due to high cholesterol. However, the exact mechanism needs to be further explored. Bone homeostasis is maintained by osteoclastic bone resorption and osteoblastic bone formation. Experimental animal studies have shown that osteoclast functions are significantly cholesterol-dependent.^543,544^ A high cholesterol diet leads to increased osteoclast numbers and bone resorption.^544^ Conversely, inhibition of proliferation and differentiation of osteoblast MC3T3-E1 cells by cholesterol was determined in a dose-dependent manner, while resulted in decreased expression of the bone formation markers, bone morphogenetic protein-2 and runt-related transcription factor 2.

The clinical use of statins to prevent and/or treat osteoporosis is controversial. In 2018, an investigation found a reduced risk of osteoporosis in stroke patients using statins.^545^ Ann et al. showed that statin increased BMD and appeared to be more effective in men with osteoporosis by meta-analysis.^546^ However, in 2019, a cross-sectional retrospective study of healthy subjects reported that high doses of statins significantly increased the risk of osteoporosis.^547^ This may indicate that statins are more appropriate for patients with severe hypercholesterolemia and high risk for osteoporosis. Furthermore, less of the statins reach the bone after the drug has been metabolized. This explains the fact that statins are often used at much higher doses than clinical ones to relieve osteoporosis.^548^ Consequently, local delivery of statins needs further exploration.

#### Virus infection

A lipid raft is a subdomain of the plasma membrane enriched in cholesterol and sphingolipids, which also act as vectors for viruses to enter the host cells.^549,550^ Studies have shown an association between cholesterol levels and virus infections.^551–553^ Louie et al. found that additional 2% cholesterol in the diet causes inflammatory imbalance and exacerbates morbidity in mice infected with influenza A virus.^554^ Wang et al. proved that pseudorabies virus (PRV) increases self-infection capability by suppressing LXR expression to increase total intracellular cholesterol levels.^555^ COVID-19 is caused by an infection with severe acute respiratory syndrome coronavirus 2 (SARS-CoV-2). Sphingolipid- and cholesterol-rich regions recruit several receptors and molecules involved in pathogen recognition and cell signaling.^556^ Angiotensin-converting enzyme 2 (ACE2) can be recruited to these regions as the primary functional receptor for SARS-CoV-2.^556^ Therefore, cholesterol may be functionally important as a mediator of COVID-19 infection. Radenkovic et al. suggested that lipid rafts rich in ACE2 receptors may be increased in a state of high cholesterol levels, thus enhancing the endocytosis process of SARS-CoV-2.^557^ Sanders et al. proved that SARS-CoV-2 requires cholesterol for viral entry and pathological syncytia formation.^558^ Similarly, Li et al. also found that cholesterol depletion impaired virus entry in vitro.^559,560^ In addition, cholesterol plays a role in binding and altering the SARS-CoV N-terminal fusion peptide oligomeric state, which is required for virus entry into the host cells.^561^ Although many reports suggest that cholesterol plays an important role in virus entry, this still needs to be confirmed in vivo. In particular, the effect of SARS-CoV-2 on cholesterol homeostasis remains unclear and the molecular mechanisms need to be further explored.

PCSK9 is another interesting mediator involved in viral infection. Several clinical studies have found that hepatitis C virus (HCV) infection is associated with increased PCSK9 serum levels.^562–564^ PCSK9 negatively regulates the hepatocyte surface proteins (LDLR, SR-BI, VLDLR) involved in HCV entry in vitro.^565^ Meanwhile, HCV infection upregulates PCSK9 expression.^566^ This indicated a complex interaction between PCSK9 and HCV. A recent preclinical study indicated that dengue virus (DENV) infection also induced PCSK9 expression, which led to downregulation of LDLR expression with a sequester of cholesterol in the intracellular space, providing a more favorable environment for virus entry.^567^ Therefore, PCSK9 appears to contribute to DENV infection. However, the relationship between PCSK9 and SARS-CoV-2 infection is unclear.

25-hydroxycholesterol (25HC) is one of the metabolites of cholesterol catalyzed by CH25H.^568^ Unlike cholesterol, 25HC and its synthetic enzyme CH25H have been shown to have potent broad-spectrum antiviral activity.^569^ Li et al. reported that 25HC and CH25H protected hosts from Zika virus infection in a mouse model.^570^ Xiang et al. found that 25HC and CH25H inhibited HCV infection by blocking SREBP maturation to inhibit viral genome replication.^571^ Similarly, several studies have also shown that 25HC and CH25H inhibit SARS-CoV-2 infection by blocking membrane fusion.^572,573^ LXR has been shown to induce the activation of interferon-γ (IFN-γ), which stimulates the expression of CH25H.^569,574^ Interestingly, our studies reported that 25HC can also induce CH25H expression in an LXR-dependent manner, and demonstrated that LXR activation, interaction between CH25H and IFN-γ, and 25HC metabolism may form an antiviral system in which LXR plays a central role.^575,576^

There is an interaction between COVID-19 infection and CVD. Li et al. reported an increased prevalence of CVD in patients after COVID-19 infection.^577^ Similarly, patients infected by COVID-19 who previously experienced CVD had an increased case fatality rate.^578^ Thus, lowering cholesterol levels may reduce the risk of COVID-19-induced complications. Statins have been reported to have anti-viral activity.^579^ Therefore, they were quickly used in clinical trials for patients with COVID-19 infection. An observational study of hospitalized COVID-19 infected patients indicated that statins might be effective against COVID-19.^580^ Similar observations have been reported in several subsequent studies.^581–583^ Subir et al. recommended that COVID-19 infected patients at a high CVD risk should continue statin therapy unless absolutely contraindicated.^584^ Statins may lower membrane cholesterol levels, thereby decreasing the attachment and internalization of SARS-CoV-2.^557^ Surprisingly, Reiner et al. identified several statins as potential SARS-CoV-2 major protease inhibitors by molecular docking, especially pitavastatin with the strongest binding.^585^ Therefore, the benefits of statins for patients with COVID-19 may be exerted through their direct cholesterol lowering effects and beyond. Future research is needed to depict the precise mechanism of cholesterol-aimed viral entry, survival and discover the new cholesterol-lowering therapies in COVID-19 patients. In addition, a preclinical study has shown that LXR agonist, T0901317, significantly inhibits herpes simplex virus type 1 infection.^576^ Similarly, T0901317 also showed better prevention of PRV infection in mice.^555^ A monoclonal antibody of PCSK9 (alirocumab) was shown to inhibit DENV infection in vitro.^567^ Boccara et al. firstly evaluated the efficacy and safety of evolocumab in reducing LDL-C levels in HIV patients in a multinational, randomized, double-blind study.^586^ However, no clinical trials on the effects of PCSK9 inhibitors in SARS-CoV-2-infected patients to date. Nevertheless, experts believe that use of PCSK9 inhibitors is still beneficial for COVID-19 patients with familial hypercholesterolemia.^587,588^

## Summary and outlook

High circulating cholesterol level is a major risk factor for ASCVD and promotes the progression of atherosclerosis, making key molecules involved in cholesterol homeostasis as the attractive therapeutic targets for ASCVD treatment. By reducing cholesterol biosynthesis and enhancing cholesterol metabolism, statins are used widely to reduce the levels of plasma TC and LDL-C to prevent or reduce CVD. However, due to the side effects and intolerance of statins, non-statin cholesterol-lowering drugs are being developed and more other novel targets than cholesterol lowering have been characterized. Moreover, combination of non-statin cholesterol-lowering drugs (for example, ezetimibe or PCSK9 inhibitors) with statins may be more effective in reducing LDL-C levels. A very exciting development is the concept “the lower the better” of LDL-C reduction, indicating that a lower LDL-C is tightly correlated to a better attenuation of ASCVD. In addition, cholesterol lowering has been demonstrated to be beneficial in many other diseases (Table 3). Therefore, cholesterol-lowering therapy is a rapidly developing field with various new targets and drugs.

**Table 3:** Cholesterol and diseases

Diseases	Cholesterol-induced pathogenesis	Cholesterol-lowering therapies
ASCVD	Promotes macrophage foaminess	refer to Table 1
NAFLD	Induces inflammation, Küpffer cell foaminess, formation of “crown-like structures”	Statins and ezetimibe (controversial) in clinical studies; Lanifibranor in animal model; Potential new targets: *SH3RF2*, miRNAs
Obesity	Induces inflammation in adipose tissue, less thermogenic effect	Triiodothyronine in clinical studies; Diet and lifestyle changes; Potential new targets: β3-adrenergic receptor, GC-1
Diabetes	Induces islet β-cell dysfunction; Induces inflammation, oxidative stress and ER stress	CETP inhibitor in clinical studies; Potential new targets: miR-33a and miR-145
Neurodegenerative diseases	Increases Aβ, p-Tau and NFTs: Reduces Aβ clearance Increases α-synuclein aggregates mHTT leads to an unbalanced cholesterol homeostasis	Statins (controversial) in clinical studies; Ewfavirenz, BM15.766, LXRs agonizts and β-cyclodextrins in animal model
Cancer	Promotes the process of cancer; Leads to T cell exhaustion	Statins in clinical studies; Avacizimibe, T0901317 and ezetimibe in animal model
Osteoporosis	Increases bone resorption; Decreases bone formation	Statins in clinical studies (controversial)
Virus infection	Increases the density of lipid rafts; Promotes viral endocytosis 25HC and CH25H inhibit virus infection PCSK9 promotes DENV infection PCSK9 inhibits HCV infection	Statins in clinical studies T0901317 in animal model Evolocumab in clinical study Alirocumab in animal model

In the future, the investigations related to cholesterol may face more challenges. For example, characterizing the relationship between inflammation and cholesterol metabolic disorders and developing the specific anti-inflammatory therapeutic intervention in reducing inflammation in ASCVD. Beyond LDL-C, the intervention on other lipoproteins needs more efforts to investigate. Nowadays, various cholesterol-lowering drugs are used in clinics. However, the studies on personalized therapy, lifestyle and targeting the right patient with the right time still need more attention. Moreover, exploring the role of cholesterol in other diseases, especially the complications of metabolic disorders, may accelerate the translation of research to the clinic.

## References

[1] Vance DE, Van den Bosch H (2000). Cholesterol in the year 2000. Biochim. Biophys. Acta.

[2] Nicholls M (2019). Adolf otto reinhold windaus. Eur. Heart J..

[3] Kennedy EP, Westheimer FH (1964). Nobel laureates: Bloch and Lynen win prize in medicine and physiology. Science.

[4] Brown MS, Goldstein JL (2009). Cholesterol feedback: from Schoenheimer’s bottle to Scap’s MELADL. J. Lipid Res..

[5] Schoenheimer R, Breusch F (1933). Synthesis and destruction of cholesterol in the organism. J. Biol. Chem..

[6] Brown MS, Goldstein JL (1997). The SREBP pathway: regulation of cholesterol metabolism by proteolysis of a membrane-bound transcription factor. Cell.

[7] Brown MS, Ye J, Rawson RB, Goldstein JL (2000). Regulated intramembrane proteolysis: a control mechanism conserved from bacteria to humans. Cell.

[8] Goldstein JL, Brown MS (2015). A century of cholesterol and coronaries: from plaques to genes to statins. Cell.

[9] Endo A (2010). A historical perspective on the discovery of statins. Proc. Jpn Acad. Ser. B Phys. Biol. Sci..

[10] N. A (1913). Über die veränderungen der kaninchenaorta bei experimenteller cholesterinsteatose. Beitr. Pathol. Anat..

[11] Lee YT (2017). Mouse models of atherosclerosis: a historical perspective and recent advances. Lipids Health Dis..

[12] Tall AR, Yvan-Charvet L (2015). Cholesterol, inflammation and innate immunity. Nat. Rev. Immunol..

[13] Grebe A, Latz E (2013). Cholesterol crystals and inflammation. Curr. Rheumatol. Rep..

[14] Baumer Y, Mehta NN, Dey AK, Powell-Wiley TM, Boisvert WA (2020). Cholesterol crystals and atherosclerosis. Eur. Heart J..

[15] Rajamaki K (2010). Cholesterol crystals activate the NLRP3 inflammasome in human macrophages: a novel link between cholesterol metabolism and inflammation. PLoS One.

[16] Duewell P (2010). NLRP3 inflammasomes are required for atherogenesis and activated by cholesterol crystals. Nature.

[17] Janoudi A, Shamoun FE, Kalavakunta JK, Abela GS (2016). Cholesterol crystal induced arterial inflammation and destabilization of atherosclerotic plaque. Eur. Heart J..

[18] Soehnlein O, Libby P (2021). Targeting inflammation in atherosclerosis—from experimental insights to the clinic. Nat. Rev. Drug Discov..

[19] Libby P, Ridker PM, Maseri A (2002). Inflammation and atherosclerosis. Circulation.

[20] Gofman JW, Lindgren FT, Elliott H (1949). Ultracentrifugal studies of lipoproteins of human serum. J. Biol. Chem..

[21] Gofman JW, Lindgren F (1950). The role of lipids and lipoproteins in atherosclerosis. Science.

[22] Gofman JW (1956). Serum lipoproteins and the evaluation of atherosclerosis. Ann. N. Y. Acad. Sci..

[23] Castelli WP, Anderson K, Wilson PW, Levy D (1992). Lipids and risk of coronary heart disease. framingham study Ann. Epidemiol..

[24] Müller C (1938). Xanthomata, hypercholesterolemia, angina pectoris. Acta Med. Scand..

[25] Khachadurian AK (1964). The inheritance of essential familial hypercholesterolemia. Am. J. Med..

[26] Steinberg D (2005). Thematic review series: The pathogenesis of atherosclerosis: an interpretive history of the cholesterol controversy, part III: Mechanistically defining the role of hyperlipidemia. J. Lipid Res..

[27] Brown MS, Goldstein JL (1986). A receptor-mediated pathway for cholesterol homeostasis. Science.

[28] Grundy, S. M. & Feingold, K. R. Guidelines for the Management of High Blood Cholesterol. in Endotext (eds K. R. Feingold et al.) (2000).

[29] Endo A, Kuroda M, Tanzawa K (1976). Competitive inhibition of 3-hydroxy-3-methylglutaryl coenzyme A reductase by ML-236A and ML-236B fungal metabolites, having hypocholesterolemic activity. FEBS Lett..

[30] Brown MS, Faust JR, Goldstein JL, Kaneko I, Endo A (1978). Induction of 3-hydroxy-3-methylglutaryl coenzyme A reductase activity in human fibroblasts incubated with compactin (ML-236B), a competitive inhibitor of the reductase. J. Biol. Chem..

[31] Cho L (2020). A practical approach to the cholesterol guidelines and ASCVD prevention. Cleve Clin. J. Med..

[32] Davidson MH (2003). Efficacy of simvastatin and ezetimibe in treating hypercholesterolemia. J. Am. Coll. Cardiol..

[33] Seidah NG (2003). The secretory proprotein convertase neural apoptosis-regulated convertase 1 (NARC-1): Liver regeneration and neuronal differentiation. Proc. Natl Acad. Sci. USA.

[34] Lagace TA (2006). Secreted PCSK9 decreases the number of LDL receptors in hepatocytes and in livers of parabiotic mice. J. Clin. Investig..

[35] Zhang DW (2007). Binding of proprotein convertase subtilisin/kexin type 9 to epidermal growth factor-like repeat A of low density lipoprotein receptor decreases receptor recycling and increases degradation. J. Biol. Chem..

[36] Kim EJ, Wierzbicki AS (2020). The history of proprotein convertase subtilisin kexin-9 inhibitors and their role in the treatment of cardiovascular disease. Ther. Adv. Chronic Dis..

[37] Lamb YN (2021). Inclisiran: first approval. Drugs.

[38] Sanjay KV (2021). ATP citrate lyase inhibitor Bempedoic acid alleviate long term HFD induced NASH through improvement in glycemic control, reduction of hepatic triglycerides & total cholesterol, modulation of inflammatory & fibrotic genes and improvement in NAS score. Curr. Res Pharm. Drug Discov..

[39] Govindaraju A, Sabarathinam S (2021). Bempedoic acid: a nonstatin drug for the management of hypercholesterolemia. Health Sci. Rep..

[40] Feng X, Zhang L, Xu S, Shen AZ (2020). ATP-citrate lyase (ACLY) in lipid metabolism and atherosclerosis: an updated review. Prog. Lipid Res..

[41] Kapourchali FR, Surendiran G, Goulet A, Moghadasian MH (2016). The role of dietary cholesterol in lipoprotein metabolism and related metabolic abnormalities: a mini-review. Crit. Rev. Food Sci. Nutr..

[42] Rosenson RS, Song WL (2019). Egg yolk, source of bad cholesterol and good lipids?. Am. J. Clin. Nutr..

[43] Myocardial Infarction Genetics Consortium, I. (2014). Inactivating mutations in NPC1L1 and protection from coronary heart disease. N. Engl. J. Med.

[44] Iqbal J, Hussain MM (2009). Intestinal lipid absorption. Am. J. Physiol. Endocrinol. Metab..

[45] Altmann SW (2004). Niemann-Pick C1 Like 1 protein is critical for intestinal cholesterol absorption. Science.

[46] Fumeron F, Bard JM, Lecerf JM (2017). Interindividual variability in the cholesterol-lowering effect of supplementation with plant sterols or stanols. Nutr. Rev..

[47] Cusack LK, Fernandez ML, Volek JS (2013). The food matrix and sterol characteristics affect the plasma cholesterol lowering of phytosterol/phytostanol. Adv. Nutr..

[48] Huff MW, Pollex RL, Hegele RA (2006). NPC1L1: evolution from pharmacological target to physiological sterol transporter. Arterioscler. Thromb. Vasc. Biol..

[49] Dietschy JM, Gamel WG (1971). Cholesterol synthesis in the intestine of man: regional differences and control mechanisms. J. Clin. Investig..

[50] Nestel PJ, Poyser A (1976). Changes in cholesterol synthesis and excretion when cholesterol intake is increased. Metabolism.

[51] Rudney H, Sexton RC (1986). Regulation of cholesterol biosynthesis. Annu. Rev. Nutr..

[52] Bloch K (1965). The biological synthesis of cholesterol. Science.

[53] Goldstein JL, Brown MS (1990). Regulation of the mevalonate pathway. Nature.

[54] DeBose-Boyd RA (2008). Feedback regulation of cholesterol synthesis: sterol-accelerated ubiquitination and degradation of HMG CoA reductase. Cell Res.

[55] Yokoyama C (1993). SREBP-1, a basic-helix-loop-helix-leucine zipper protein that controls transcription of the low density lipoprotein receptor gene. Cell.

[56] Gong X (2016). Complex structure of the fission yeast SREBP-SCAP binding domains reveals an oligomeric organization. Cell Res.

[57] Brown MS, Radhakrishnan A, Goldstein JL (2018). Retrospective on cholesterol homeostasis: the central role of SCAP. Annu. Rev. Biochem..

[58] Luo J, Yang H, Song BL (2020). Mechanisms and regulation of cholesterol homeostasis. Nat. Rev. Mol. Cell Biol..

[59] Kober DL (2021). SCAP structures highlight key role for rotation of intertwined luminal loops in cholesterol sensing. Cell.

[60] Yan R (2021). A structure of human SCAP bound to INSIG-2 suggests how their interaction is regulated by sterols. Science.

[61] Sharpe LJ, Coates HW, Brown AJ (2020). Post-translational control of the long and winding road to cholesterol. J. Biol. Chem..

[62] Luskey KL, Stevens B (1985). Human 3-hydroxy-3-methylglutaryl coenzyme A reductase. Conserved domains responsible for catalytic activity and sterol-regulated degradation. J. Biol. Chem..

[63] Song BL, Sever N, DeBose-Boyd RA (2005). Gp78, a membrane-anchored ubiquitin ligase, associates with INSIG-1 and couples sterol-regulated ubiquitination to degradation of HMG CoA reductase. Mol. Cell.

[64] Cao J (2007). Ufd1 is a cofactor of gp78 and plays a key role in cholesterol metabolism by regulating the stability of HMG-CoA reductase. Cell Metab..

[65] Lee JN, Song B, DeBose-Boyd RA, Ye J (2006). Sterol-regulated degradation of INSIG-1 mediated by the membrane-bound ubiquitin ligase gp78. J. Biol. Chem..

[66] Liu TF (2012). Ablation of gp78 in liver improves hyperlipidemia and insulin resistance by inhibiting SREBP to decrease lipid biosynthesis. Cell Metab..

[67] Lu XY (2020). Feeding induces cholesterol biosynthesis via the mTORC1-USP20-HMGCR axis. Nature.

[68] Clarke PR, Hardie DG (1990). Regulation of HMG-CoA reductase: Identification of the site phosphorylated by the AMP-activated protein kinase in vitro and in intact rat liver. EMBO J..

[69] Sato R, Goldstein JL, Brown MS (1993). Replacement of serine-871 of hamster 3-hydroxy-3-methylglutaryl-CoA reductase prevents phosphorylation by AMP-activated kinase and blocks inhibition of sterol synthesis induced by ATP depletion. Proc. Natl Acad. Sci. USA.

[70] min HK (2012). Increased hepatic synthesis and dysregulation of cholesterol metabolism is associated with the severity of nonalcoholic fatty liver disease. Cell Metab..

[71] Zhang X (2015). Thyroid-stimulating hormone decreases HMG-CoA reductase phosphorylation via AMP-activated protein kinase in the liver. J. Lipid Res..

[72] Rosenson RS (2012). Cholesterol efflux and atheroprotection: advancing the concept of reverse cholesterol transport. Circulation.

[73] van der Velde AE (2007). Direct intestinal cholesterol secretion contributes significantly to total fecal neutral sterol excretion in mice. Gastroenterology.

[74] Schwartz CC, Vlahcevic ZR, Halloran LG, Swell L (1981). An in vivo evaluation in man of the transfer of esterified cholesterol between lipoproteins and into the liver and bile. Biochim. Biophys. Acta.

[75] Brandts J, Ray KK (2021). Familial hypercholesterolemia: JACC focus seminar 4/4. J. Am. Coll. Cardiol..

[76] Yu L (2006). Cholesterol-regulated translocation of NPC1L1 to the cell surface facilitates free cholesterol uptake. J. Biol. Chem..

[77] Wang J (2009). Membrane topology of human NPC1L1, a key protein in enterohepatic cholesterol absorption. J. Lipid Res..

[78] Infante RE (2008). Purified NPC1L1 protein: II. Localization of sterol binding to a 240-amino acid soluble luminal loop. J. Biol. Chem..

[79] Xie C (2012). Ezetimibe blocks the internalization of NPC1L1 and cholesterol in mouse small intestine. J. Lipid Res..

[80] Li PS (2014). The clathrin adaptor Numb regulates intestinal cholesterol absorption through dynamic interaction with NPC1L1. Nat. Med..

[81] Johnson TA, Pfeffer SR (2016). Ezetimibe-sensitive cholesterol uptake by NPC1L1 protein does not require endocytosis. Mol. Biol. Cell.

[82] Huang CS (2020). Cryo-EM structures of NPC1L1 reveal mechanisms of cholesterol transport and ezetimibe inhibition. Sci. Adv..

[83] Hu M (2021). Structural insights into the mechanism of human NPC1L1-mediated cholesterol uptake. Sci. Adv..

[84] Long T, Liu Y, Qin Y, DeBose-Boyd RA, Li X (2021). Structures of dimeric human NPC1L1 provide insight into mechanisms for cholesterol absorption. Sci. Adv..

[85] Clader JW (2004). The discovery of ezetimibe: a view from outside the receptor. J. Med. Chem..

[86] Zhang R (2022). Niemann-Pick C1-Like 1 inhibitors for reducing cholesterol absorption. Eur. J. Med. Chem..

[87] Jia L, Betters JL, Yu L (2011). Niemann-Pick C1-Like 1 (NPC1L1) protein in intestinal and hepatic cholesterol transport. Annu. Rev. Physiol..

[88] Yan J, Horng T (2020). Lipid metabolism in regulation of macrophage functions. Trends Cell Biol..

[89] Chistiakov DA, Melnichenko AA, Myasoedova VA, Grechko AV, Orekhov AN (2017). Mechanisms of foam cell formation in atherosclerosis. J. Mol. Med..

[90] Dandan M (2021). Turnover rates of the low-density lipoprotein receptor and PCSK9: added dimension to the cholesterol homeostasis model. Arterioscler Thromb. Vasc. Biol..

[91] Moore KJ, Freeman MW (2006). Scavenger receptors in atherosclerosis: beyond lipid uptake. Arterioscler. Thromb. Vasc. Biol..

[92] Kzhyshkowska J, Neyen C, Gordon S (2012). Role of macrophage scavenger receptors in atherosclerosis. Immunobiology.

[93] Kunjathoor VV (2002). Scavenger receptors class A-I/II and CD36 are the principal receptors responsible for the uptake of modified low density lipoprotein leading to lipid loading in macrophages. J. Biol. Chem..

[94] Wang D (2019). Targeting foam cell formation in atherosclerosis: therapeutic potential of natural products. Pharm. Rev..

[95] Moore KJ, Sheedy FJ, Fisher EA (2013). Macrophages in atherosclerosis: a dynamic balance. Nat. Rev. Immunol..

[96] Melton EM (2019). Myeloid Acat1/Soat1 KO attenuates pro-inflammatory responses in macrophages and protects against atherosclerosis in a model of advanced lesions. J. Biol. Chem..

[97] Huang LH (2016). Myeloid Acyl-CoA: cholesterol acyltransferase 1 deficiency reduces lesion macrophage content and suppresses atherosclerosis progression. J. Biol. Chem..

[98] Shao D (2020). Grape seed proanthocyanidins suppressed macrophage foam cell formation by miRNA-9 via targeting ACAT1 in THP-1 cells. Food Funct..

[99] Wang B, He PP, Zeng GF, Zhang T, Ou Yang XP (2017). miR-467b regulates the cholesterol ester formation via targeting ACAT1 gene in RAW 264.7 macrophages. Biochimie.

[100] Meuwese MC (2009). ACAT inhibition and progression of carotid atherosclerosis in patients with familial hypercholesterolemia: the CAPTIVATE randomized trial. JAMA.

[101] Tardif JC (2004). Effects of the acyl coenzyme A: cholesterol acyltransferase inhibitor avasimibe on human atherosclerotic lesions. Circulation.

[102] Warner GJ, Stoudt G, Bamberger M, Johnson WJ, Rothblat GH (1995). Cell toxicity induced by inhibition of acyl coenzyme A: cholesterol acyltransferase and accumulation of unesterified cholesterol. J. Biol. Chem..

[103] Ouimet M, Marcel YL (2012). Regulation of lipid droplet cholesterol efflux from macrophage foam cells. Arterioscler. Thromb. Vasc. Biol..

[104] Zhao B, Song JSt, Clair RW, Ghosh S (2007). Stable overexpression of human macrophage cholesteryl ester hydrolase results in enhanced free cholesterol efflux from human THP1 macrophages. Am. J. Physiol. Cell Physiol..

[105] Sekiya M (2009). Ablation of neutral cholesterol ester hydrolase 1 accelerates atherosclerosis. Cell Metab..

[106] Favari E (2015). Cholesterol efflux and reverse cholesterol transport. Handb. Exp. Pharm..

[107] Adorni MP (2007). The roles of different pathways in the release of cholesterol from macrophages. J. Lipid Res..

[108] Yancey PG (2003). Importance of different pathways of cellular cholesterol efflux. Arterioscler. Thromb. Vasc. Biol..

[109] Anastasius M (2016). Cholesterol efflux capacity: an introduction for clinicians. Am. Heart J..

[110] Qian H (2017). Structure of the human lipid exporter ABCA1. Cell.

[111] Wang N, Lan D, Chen W, Matsuura F, Tall AR (2004). ATP-binding cassette transporters G1 and G4 mediate cellular cholesterol efflux to high-density lipoproteins. Proc. Natl Acad. Sci. USA.

[112] Out R (2008). Combined deletion of macrophage ABCA1 and ABCG1 leads to massive lipid accumulation in tissue macrophages and distinct atherosclerosis at relatively low plasma cholesterol levels. Arterioscler. Thromb. Vasc. Biol..

[113] Costet P, Luo Y, Wang N, Tall AR (2000). Sterol-dependent transactivation of the ABC1 promoter by the liver X receptor/retinoid X receptor. J. Biol. Chem..

[114] Shen WJ, Azhar S, Kraemer FB (2018). SR-BI: A unique multifunctional receptor for cholesterol influx and efflux. Annu. Rev. Physiol..

[115] Lim HY (2013). Lymphatic vessels are essential for the removal of cholesterol from peripheral tissues by SR-BI-mediated transport of HDL. Cell Metab..

[116] Phillips MC (2014). Molecular mechanisms of cellular cholesterol efflux. J. Biol. Chem..

[117] De Lalla OF, Gofman JW (1954). Ultracentrifugal analysis of serum lipoproteins. Methods Biochem. Anal..

[118] Kontush A (2015). Structure of HDL: particle subclasses and molecular components. Handb. Exp. Pharm..

[119] Marques LR (2018). Reverse cholesterol transport: molecular mechanisms and the non-medical approach to enhance HDL cholesterol. Front. Physiol..

[120] Glickman RM, Green PH, Lees RS, Tall A (1978). Apoprotein A-I synthesis in normal intestinal mucosa and in Tangier disease. N. Engl. J. Med..

[121] Dieplinger H, Zechner R, Kostner GM (1985). The in vitro formation of HDL2 during the action of LCAT: The role of triglyceride-rich lipoproteins. J. Lipid Res..

[122] Vaughan AM, Oram JF (2005). ABCG1 redistributes cell cholesterol to domains removable by high density lipoprotein but not by lipid-depleted apolipoproteins. J. Biol. Chem..

[123] Ji Y (1997). Scavenger receptor BI promotes high density lipoprotein-mediated cellular cholesterol efflux. J. Biol. Chem..

[124] Fielding CJ, Shore VG, Fielding PE (1972). A protein cofactor of lecithin: cholesterol acyltransferase. Biochem. Biophys. Res. Commun..

[125] Jonas A (1991). Lecithin-cholesterol acyltransferase in the metabolism of high-density lipoproteins. Biochim. Biophys. Acta.

[126] Bruce C, Beamer LJ, Tall AR (1998). The implications of the structure of the bactericidal/permeability-increasing protein on the lipid-transfer function of the cholesteryl ester transfer protein. Curr. Opin. Struct. Biol..

[127] Group HTC (2014). Effects of extended-release niacin with laropiprant in high-risk patients. N. Engl. J. Med..

[128] Davidson MH (2012). HDL and CETP inhibition: will this define the future?. Curr. Treat. Options Cardiovasc. Med..

[129] Furtado JD (2022). Pharmacological inhibition of CETP (cholesteryl ester transfer protein) increases HDL (high-density lipoprotein) that contains APOC3 and other HDL subspecies associated with higher risk of coronary heart disease. Arterioscler. Thromb. Vasc. Biol..

[130] Rousset X, Shamburek R, Vaisman B, Amar M, Remaley AT (2011). Lecithin cholesterol acyltransferase: an anti- or pro-atherogenic factor?. Curr. Atheroscler. Rep..

[131] Pownall HJ, Rosales C, Gillard BK, Gotto AM (2021). High-density lipoproteins, reverse cholesterol transport and atherogenesis. Nat. Rev. Cardiol..

[132] Madsen CM, Varbo A, Nordestgaard BG (2017). Extreme high high-density lipoprotein cholesterol is paradoxically associated with high mortality in men and women: two prospective cohort studies. Eur. Heart J..

[133] Hamer M, O’Donovan G, Stamatakis E (2018). High-density lipoprotein cholesterol and mortality: too much of a good thing?. Arterioscler. Thromb. Vasc. Biol..

[134] Nordestgaard BG, Tybjaerg-Hansen A (1992). IDL, VLDL, chylomicrons and atherosclerosis. Eur. J. Epidemiol..

[135] Barter PJ (2003). Cholesteryl ester transfer protein: a novel target for raising HDL and inhibiting atherosclerosis. Arterioscler. Thromb. Vasc. Biol..

[136] Brown MS, Goldstein JL (1984). How LDL receptors influence cholesterol and atherosclerosis. Sci. Am..

[137] Shu H (2017). A novel indel variant in LDLR responsible for familial hypercholesterolemia in a Chinese family. PLoS One.

[138] Jiang L (2017). Analysis of LDLR variants from homozygous FH patients carrying multiple mutations in the LDLR gene. Atherosclerosis.

[139] Calkin AC (2011). FERM-dependent E3 ligase recognition is a conserved mechanism for targeted degradation of lipoprotein receptors. Proc. Natl Acad. Sci. USA.

[140] Wang JK (2022). Ablation of plasma prekallikrein decreases low-density lipoprotein cholesterol by stabilizing low-density lipoprotein receptor and protects against atherosclerosis. Circulation.

[141] Liscum L, Underwood KW (1995). Intracellular cholesterol transport and compartmentation. J. Biol. Chem..

[142] Simons K, Ikonen E (2000). How cells handle cholesterol. Science.

[143] Harris JS, Epps DE, Davio SR, Kezdy FJ (1995). Evidence for transbilayer, tail-to-tail cholesterol dimers in dipalmitoylglycerophosphocholine liposomes. Biochemistry.

[144] Mukherjee S, Chattopadhyay A (1996). Membrane organization at low cholesterol concentrations: a study using 7-nitrobenz-2-oxa-1,3-diazol-4-yl-labeled cholesterol. Biochemistry.

[145] Tulenko TN, Chen M, Mason PE, Mason RP (1998). Physical effects of cholesterol on arterial smooth muscle membranes: evidence of immiscible cholesterol domains and alterations in bilayer width during atherogenesis. J. Lipid Res..

[146] Simons K, Ikonen E (1997). Functional rafts in cell membranes. Nature.

[147] Xu X, London E (2000). The effect of sterol structure on membrane lipid domains reveals how cholesterol can induce lipid domain formation. Biochemistry.

[148] Pucadyil TJ, Chattopadhyay A (2006). Role of cholesterol in the function and organization of G-protein coupled receptors. Prog. Lipid Res..

[149] Hong C, Tontonoz P (2014). Liver X receptors in lipid metabolism: opportunities for drug discovery. Nat. Rev. Drug Discov..

[150] Radhakrishnan A, Ikeda Y, Kwon HJ, Brown MS, Goldstein JL (2007). Sterol-regulated transport of SREBPs from endoplasmic reticulum to Golgi: oxysterols block transport by binding to INSIG. Proc. Natl Acad. Sci. USA.

[151] Mitton JR, Scholan NA, Boyd GS (1971). The oxidation of cholesterol in rat liver sub-cellular particles. The cholesterol-7-alpha-hydroxylase enzyme system. Eur. J. Biochem..

[152] Cali JJ, Russell DW (1991). Characterization of human sterol 27-hydroxylase. A mitochondrial cytochrome P-450 that catalyzes multiple oxidation reaction in bile acid biosynthesis. J. Biol. Chem..

[153] Lund EG, Kerr TA, Sakai J, Li WP, Russell DW (1998). cDNA cloning of mouse and human cholesterol 25-hydroxylases, polytopic membrane proteins that synthesize a potent oxysterol regulator of lipid metabolism. J. Biol. Chem..

[154] Lappano R (2011). The cholesterol metabolite 25-hydroxycholesterol activates estrogen receptor α-mediated signaling in cancer cells and in cardiomyocytes. PLoS One.

[155] Miller WL, Strauss JF (1999). Molecular pathology and mechanism of action of the steroidogenic acute regulatory protein, StAR. J. Steroid Biochem. Mol. Biol..

[156] Temel RE, Brown JM (2015). A new model of reverse cholesterol transport: enTICEing strategies to stimulate intestinal cholesterol excretion. Trends Pharm. Sci..

[157] Yu XH (2014). ABCG5/ABCG8 in cholesterol excretion and atherosclerosis. Clin. Chim. Acta.

[158] Li G, Gu HM, Zhang DW (2013). ATP-binding cassette transporters and cholesterol translocation. IUBMB Life.

[159] Vrins C (2007). The sterol transporting heterodimer ABCG5/ABCG8 requires bile salts to mediate cholesterol efflux. FEBS Lett..

[160] Johnson BJ, Lee JY, Pickert A, Urbatsch IL (2010). Bile acids stimulate ATP hydrolysis in the purified cholesterol transporter ABCG5/G8. Biochemistry.

[161] Norlin M, Wikvall K (2007). Enzymes in the conversion of cholesterol into bile acids. Curr. Mol. Med..

[162] de Aguiar Vallim TQ, Tarling EJ, Edwards PA (2013). Pleiotropic roles of bile acids in metabolism. Cell Metab..

[163] Ridlon JM, Alves JM, Hylemon PB, Bajaj JS (2013). Cirrhosis, bile acids and gut microbiota: unraveling a complex relationship. Gut Microbes.

[164] Lee JY (2016). Crystal structure of the human sterol transporter ABCG5/ABCG8. Nature.

[165] Back SS (2013). Cooperative transcriptional activation of atp-binding cassette sterol transporters ABCG5 and ABCG8 genes by nuclear receptors including Liver-X-Receptor. BMB Rep..

[166] van der Veen JN (2009). Activation of the liver X receptor stimulates trans-intestinal excretion of plasma cholesterol. J. Biol. Chem..

[167] van der Velde AE, Brufau G, Groen AK (2010). Transintestinal cholesterol efflux. Curr. Opin. Lipido.

[168] de Boer JF (2017). Transintestinal and biliary cholesterol secretion both contribute to macrophage reverse cholesterol transport in rats-brief report. Arterioscler Thromb. Vasc. Biol..

[169] Jakulj L (2016). Transintestinal cholesterol transport is active in mice and humans and controls ezetimibe-induced fecal neutral sterol excretion. Cell Metab..

[170] Temel RE (2010). Biliary sterol secretion is not required for macrophage reverse cholesterol transport. Cell Metab..

[171] Stoger JL (2016). Deleting myeloid IL-10 receptor signalling attenuates atherosclerosis in LDLR−/− mice by altering intestinal cholesterol fluxes. Thromb. Haemost..

[172] Stender S, Frikke-Schmidt R, Nordestgaard BG, Tybjaerg-Hansen A (2014). The ABCG5/8 cholesterol transporter and myocardial infarction versus gallstone disease. J. Am. Coll. Cardiol..

[173] Vrins CLJ (2012). Trans-intestinal cholesterol efflux is not mediated through high density lipoprotein. J. Lipid Res..

[174] Le May C (2013). Transintestinal cholesterol excretion is an active metabolic process modulated by PCSK9 and statin involving ABCB1. Arterioscler. Thromb. Vasc. Biol..

[175] Stange EF, Dietschy JM (1983). Cholesterol synthesis and low density lipoprotein uptake are regulated independently in rat small intestinal epithelium. Proc. Natl Acad. Sci. USA.

[176] Tonini, C. et al. Inhibition of bromodomain and extraterminal domain (BET) proteins by JQ1 unravels a novel epigenetic modulation to control lipid homeostasis. *Int. J. Mol. Sci*. **21** (2020). 10.3390/ijms21041297 PMC7072965 32075110

[177] Malhotra P (2014). Epigenetic modulation of intestinal cholesterol transporter Niemann-Pick C1-Like 1 (NPC1L1) gene expression by DNA methylation. J. Biol. Chem..

[178] Li XJ (2021). Deficiency of histone methyltransferase SET domain-containing 2 in liver leads to abnormal lipid metabolism and HCC. Hepatology.

[179] Fan Z (2020). Brahma related gene 1 (BRG1) regulates cellular cholesterol synthesis by acting as a Co-factor for SREBP2. Front. Cell Dev. Biol..

[180] Kim H, Choi SY, Lim J, Lindroth AM, Park YJ (2020). EHMT2 inhibition induces cell death in human non-small cell lung cancer by altering the cholesterol biosynthesis pathway. Int. J. Mol. Sci..

[181] Meaney S (2014). Epigenetic regulation of cholesterol homeostasis. Front. Genet..

[182] Smith Z, Ryerson D, Kemper JK (2013). Epigenomic regulation of bile acid metabolism: emerging role of transcriptional cofactors. Mol. Cell Endocrinol..

[183] Chanda D, Xie YB, Choi HS (2010). Transcriptional corepressor SHP recruits SIRT1 histone deacetylase to inhibit LRH-1 transactivation. Nucleic Acids Res.

[184] Shafaati M, O’Driscoll R, Bjorkhem I, Meaney S (2009). Transcriptional regulation of cholesterol 24-hydroxylase by histone deacetylase inhibitors. Biochem. Biophys. Res. Commun..

[185] Blanc M (2013). The transcription factor STAT-1 couples macrophage synthesis of 25-hydroxycholesterol to the interferon antiviral response. Immunity.

[186] Gold ES (2012). ATF3 protects against atherosclerosis by suppressing 25-hydroxycholesterol-induced lipid body formation. J. Exp. Med..

[187] Libby P (2019). Atherosclerosis. Nat. Rev. Dis. Prim..

[188] Yu XH, Zhang DW, Zheng XL, Tang CK (2019). Cholesterol transport system: an integrated cholesterol transport model involved in atherosclerosis. Prog. Lipid Res..

[189] Tabas I, Williams KJ, Boren J (2007). Subendothelial lipoprotein retention as the initiating process in atherosclerosis: update and therape.utic implications. Circulation.

[190] Huang L (2019). SR-BI drives endothelial cell LDL transcytosis via DOCK4 to promote atherosclerosis. Nature.

[191] Tabas I (2010). Macrophage death and defective inflammation resolution in atherosclerosis. Nat. Rev. Immunol..

[192] Xu S (2021). Endothelial dysfunction in atherosclerotic cardiovascular diseases and beyond: from mechanism to pharmacotherapies. Pharm. Rev..

[193] Bobryshev YV (2006). Monocyte recruitment and foam cell formation in atherosclerosis. Micron.

[194] Li AC, Glass CK (2002). The macrophage foam cell as a target for therapeutic intervention. Nat. Med..

[195] Dickhout JG, Basseri S, Austin RC (2008). Macrophage function and its impact on atherosclerotic lesion composition, progression, and stability: the good, the bad, and the ugly. Arterioscler. Thromb. Vasc. Biol..

[196] Hutchins PM, Heinecke JW (2015). Cholesterol efflux capacity, macrophage reverse cholesterol transport and cardioprotective HDL. Curr. Opin. Lipido.

[197] Lao KH, Zeng L, Xu Q (2015). Endothelial and smooth muscle cell transformation in atherosclerosis. Curr. Opin. Lipido.

[198] Lusis AJ (2000). Atherosclerosis. Nature.

[199] Ho-Tin-Noe B (2017). Cholesterol crystallization in human atherosclerosis is triggered in smooth muscle cells during the transition from fatty streak to fibroatheroma. J. Pathol..

[200] Abela GS (2011). Effect of statins on cholesterol crystallization and atherosclerotic plaque stabilization. Am. J. Cardiol..

[201] Vani A, Underberg JA (2018). Lowering LDL-cholesterol and CV benefits: Is there a limit to how low ldl-c needs to be for optimal health benefits?. Clin. Pharm. Ther..

[202] Packard C, Chapman MJ, Sibartie M, Laufs U, Masana L (2021). Intensive low-density lipoprotein cholesterol lowering in cardiovascular disease prevention: opportunities and challenges. Heart.

[203] Yu D, Liao JK (2022). Emerging views of statin pleiotropy and cholesterol lowering. Cardiovasc. Res..

[204] Giugliano RP (2017). Cognitive function in a randomized trial of evolocumab. N. Engl. J. Med..

[205] Collins R (2016). Interpretation of the evidence for the efficacy and safety of statin therapy. Lancet.

[206] Ray KK (2016). Reductions in atherogenic lipids and major cardiovascular events: a pooled analysis of 10 ODYSSEY trials comparing alirocumab with control. Circulation.

[207] Cholesterol Treatment Trialists, C. (2010). Efficacy and safety of more intensive lowering of LDL cholesterol: a meta-analysis of data from 170,000 participants in 26 randomised trials. Lancet.

[208] Endo A, Kuroda M, Tsujita Y (1976). ML-236A, ML-236B, and ML-236C, new inhibitors of cholesterogenesis produced by penicillium citrinium. J. Antibiot..

[209] Scandinavian Simvastatin Survival Study Group. (1994). Randomised trial of cholesterol lowering in 4444 patients with coronary heart disease: the Scandinavian Simvastatin Survival Study (4s). Lancet.

[210] Endo A (1985). Drugs inhibiting HMG-CoA reductase. Pharm. Ther..

[211] Sirtori CR (2014). The pharmacology of statins. Pharm. Res..

[212] Welder G (2010). High-dose atorvastatin causes a rapid sustained increase in human serum PCSK9 and disrupts its correlation with LDL cholesterol. J. Lipid Res..

[213] Careskey HE (2008). Atorvastatin increases human serum levels of proprotein convertase subtilisin/kexin type 9. J. Lipid Res..

[214] Boekholdt SM (2014). Very low levels of atherogenic lipoproteins and the risk for cardiovascular events: a meta-analysis of statin trials. J. Am. Coll. Cardiol..

[215] Cholesterol Treatment Trialists C (2019). Efficacy and safety of statin therapy in older people: a meta-analysis of individual participant data from 28 randomised controlled trials. Lancet.

[216] Cholesterol Treatment Trialists C (2015). Efficacy and safety of ldl-lowering therapy among men and women: meta-analysis of individual data from 174,000 participants in 27 randomised trials. Lancet.

[217] Cholesterol Treatment Trialists C (2012). The effects of lowering ldl cholesterol with statin therapy in people at low risk of vascular disease: meta-analysis of individual data from 27 randomised trials. Lancet.

[218] Davignon J (2004). Beneficial cardiovascular pleiotropic effects of statins. Circulation.

[219] Oesterle A, Laufs U, Liao JK (2017). Pleiotropic effects of statins on the cardiovascular system. Circ. Res..

[220] Barter PJ, Brandrup-Wognsen G, Palmer MK, Nicholls SJ (2010). Effect of statins on HDL-C: a complex process unrelated to changes in LDL-C: analysis of the VOYAGER database. J. Lipid Res..

[221] Puri R (2016). The beneficial effects of raising high-density lipoprotein cholesterol depends upon achieved levels of low-density lipoprotein cholesterol during statin therapy: implications for coronary atheroma progression and cardiovascular events. Eur. J. Prev. Cardiol..

[222] Mortensen MB (2018). Statin trials, cardiovascular events, and coronary artery calcification: implications for a trial-based approach to statin therapy in mesa. *JACC Cardiovasc*. Imaging.

[223] Pedersen TR (2010). Pleiotropic effects of statins: evidence against benefits beyond LDLl-cholesterol lowering. Am. J. Cardiovasc. Drugs.

[224] Hadjiphilippou S, Ray KK (2019). Cholesterol-lowering agents. Circ. Res..

[225] Thompson PD, Panza G, Zaleski A, Taylor B (2016). Statin-associated side effects. J. Am. Coll. Cardiol..

[226] Stroes ES (2015). Statin-associated muscle symptoms: impact on statin therapy-european atherosclerosis society consensus panel statement on assessment, aetiology and management. Eur. Heart J..

[227] Howard JP (2021). Side effect patterns in a crossover trial of statin, placebo, and no treatment. J. Am. Coll. Cardiol..

[228] NICE Clinical Guidelines, No. 181. Cardiovascular disease: Risk assessment and reduction, including lipid modification National institute for health and care excellence: Guidelines (2016).

[229] Wanner C, Tonelli M (2014). Kidney Disease: Improving Global Outcomes Lipid Guideline Development Work Group, M. KDIGI Clinical Practice Guideline for Lipid Management in CKD: summary of recommendation statements and clinical approach to the patient. Kidney Int.

[230] Paseban M, Butler AE, Sahebkar A (2019). Mechanisms of statin-induced new-onset diabetes. J. Cell Physiol..

[231] Taylor FC, Huffman M, Ebrahim S (2013). Statin therapy for primary prevention of cardiovascular disease. JAMA.

[232] Tavazzi L (2008). Effect of rosuvastatin in patients with chronic heart failure (the GISSI-HF trial): a randomised, double-blind, placebo-controlled trial. Lancet.

[233] Moriarty PM (2015). Efficacy and safety of alirocumab vs ezetimibe in statin-intolerant patients, with a statin rechallenge arm: the ODYSSEY ALTERNATIVE randomized trial. J. Clin. Lipido.

[234] Stroes E (2014). Anti-PCSK9 antibody effectively lowers cholesterol in patients with statin intolerance: the GAUSS-2 randomized, placebo-controlled phase 3 clinical trial of evolocumab. J. Am. Coll. Cardiol..

[235] Mach F (2020). 2019 ESC/EAS Guidelines for the management of dyslipidaemias: lipid modification to reduce cardiovascular risk. Eur. Heart J..

[236] Knopp RH (2003). Effects of ezetimibe, a new cholesterol absorption inhibitor, on plasma lipids in patients with primary hypercholesterolemia. Eur. Heart J..

[237] Phan BA, Dayspring TD, Toth PP (2012). Ezetimibe therapy: mechanism of action and clinical update. Vasc. Health Risk Manag.

[238] Dumas LS (2017). Evaluation of antiatherogenic properties of ezetimibe using (3)h-labeled low-density-lipoprotein cholesterol and (99m)tc-cabvcam1-5 spect in apoe(-/-) mice fed the paigen diet. J. Nucl. Med..

[239] Lin X, Racette SB, Ma L, Wallendorf M, Ostlund RE (2017). Ezetimibe increases endogenous cholesterol excretion in humans. Arterioscler. Thromb. Vasc. Biol..

[240] Pandor A (2009). Ezetimibe monotherapy for cholesterol lowering in 2,722 people: systematic review and meta-analysis of randomized controlled trials. J. Intern. Med..

[241] Ouchi Y (2019). Ezetimibe lipid-lowering trial on prevention of atherosclerotic cardiovascular disease in 75 or older (EWTOPIA 75): a randomized, controlled trial. Circulation.

[242] Honda K (2018). Lipid-lowering therapy with ezetimibe decreases spontaneous atherothrombotic occlusions in a rabbit model of plaque erosion: a role of serum oxysterols. Arterioscler. Thromb. Vasc. Biol..

[243] Katzmann JL (2022). Non-statin lipid-lowering therapy over time in very-high-risk patients: effectiveness of fixed-dose statin/ezetimibe compared to separate pill combination on LDL-C. Clin. Res. Cardiol..

[244] Ballantyne CM (2020). Bempedoic acid plus ezetimibe fixed-dose combination in patients with hypercholesterolemia and high CVD risk treated with maximally tolerated statin therapy. Eur. J. Prev. Cardiol..

[245] Jones MR, Nwose OM (2013). Role of colesevelam in combination lipid-lowering therapy. Am. J. Cardiovasc. Drugs.

[246] Nissen SE (2016). Efficacy and tolerability of evolocumab vs ezetimibe in patients with muscle-related statin intolerance: the GAUSS-3 randomized clinical trial. JAMA.

[247] Kosoglou T (2005). Ezetimibe: a review of its metabolism, pharmacokinetics and drug interactions. Clin. Pharmacokinet..

[248] Norata GD, Tibolla G, Catapano AL (2014). Targeting PCSK9 for hypercholesterolemia. Annu. Rev. Pharm. Toxicol..

[249] Ding Z (2015). Cross-talk between LOX-1 and PCSK9 in vascular tissues. Cardiovasc. Res.

[250] Ding Z (2018). PCSK9 regulates expression of scavenger receptors and ox-LDL uptake in macrophages. Cardiovasc. Res.

[251] Tsouka AN, Tellis CC, Tselepis AD (2018). Pharmacology of PCSK9 inhibitors: current status and future perspectives. Curr. Pharm. Des..

[252] Migliorati JM, Jin J, Zhong X (2022). B. siRNA drug Leqvio (inclisiran) to lower cholesterol. Trends Pharmacol. Sci..

[253] AlTurki A (2019). Meta-analysis of randomized controlled trials assessing the impact of proprotein convertase subtilisin/kexin type 9 antibodies on mortality and cardiovascular outcomes. Am. J. Cardiol..

[254] Jalloh MA, Doroudgar S, Ip EJ (2018). What is the impact of the 2017 cochrane systematic review and meta-analysis that evaluated the use of PCSK9 inhibitors for lowering cardiovascular disease and mortality?. Expert Opin. Pharmacother..

[255] Karatasakis, A. et al. Effect of PCSK9 inhibitors on clinical outcomes in patients with hypercholesterolemia: a meta-analysis of 35 randomized controlled trials. *J. Am. Heart Assoc*. **6** (2017). 10.1161/JAHA.117.006910 PMC5779013 29223954

[256] Turgeon RD, Tsuyuki RT, Gyenes GT, Pearson GJ (2018). Cardiovascular efficacy and safety of PCSK9 inhibitors: systematic review and meta-analysis including the odyssey outcomes trial. Can. J. Cardiol..

[257] Ray KK (2020). Two phase 3 trials of inclisiran in patients with elevated LDL cholesterol. N. Engl. J. Med..

[258] Wang Z, Chen X, Liu J, Wang Y, Zhang S (2022). Inclisiran inhibits oxidized low-density lipoprotein-induced foam cell formation in Raw264.7 macrophages via activating the PPARγ pathway. Autoimmunity.

[259] Cicero AF, Tartagni E, Ertek S (2014). Safety and tolerability of injectable lipid-lowering drugs: a review of available clinical data. Expert Opin. Drug Saf..

[260] Markham A (2020). Bempedoic acid: first approval. Drugs.

[261] Corsini A, Scicchitano P (2021). Bempedoic acid: mechanism of action. G. Ital. Cardiol..

[262] Pinkosky SL (2016). Liver-specific ATP-citrate lyase inhibition by bempedoic acid decreases ldl-c and attenuates atherosclerosis. Nat. Commun..

[263] Laufs U (2019). Efficacy and safety of bempedoic acid in patients with hypercholesterolemia and statin intolerance. J. Am. Heart Assoc..

[264] Samsoondar JP (2017). Prevention of diet-induced metabolic dysregulation, inflammation, and atherosclerosis in LDLR^-/-^ mice by treatment with the ATP-citrate lyase inhibitor bempedoic acid. Arterioscler. Thromb. Vasc. Biol..

[265] Ballantyne CM (2018). Efficacy and safety of bempedoic acid added to ezetimibe in statin-intolerant patients with hypercholesterolemia: a randomized, placebo-controlled study. Atherosclerosis.

[266] Goldberg AC (2019). Effect of bempedoic acid vs placebo added to maximally tolerated statins on low-density lipoprotein cholesterol in patients at high risk for cardiovascular disease: the CLEAR Wisdom Randomized Clinical Trial. JAMA.

[267] Thompson PD (2016). Treatment with ETC-1002 alone and in combination with ezetimibe lowers LDL cholesterol in hypercholesterolemic patients with or without statin intolerance. J. Clin. Lipido.

[268] Powell J, Piszczatoski C (2021). Bempedoic acid: a new tool in the battle against hyperlipidemia. Clin. Ther..

[269] Ray KK (2019). Safety and efficacy of bempedoic acid to reduce LDL cholesterol. N. Engl. J. Med..

[270] Pirillo A, Catapano AL (2022). New insights into the role of bempedoic acid and ezetimibe in the treatment of hypercholesterolemia. Curr. Opin. Endocrinol. Diabetes Obes..

[271] Feng Y, Li Q, Ou G, Yang M, Du L (2021). Bile acid sequestrants: a review of mechanism and design. J. Pharm. Pharm..

[272] Ross S (2015). Effect of bile acid sequestrants on the risk of cardiovascular events: a mendelian randomization analysis. Circ. Cardiovasc. Genet..

[273] The lipid research clinics coronary primary prevention trial. (1992). Results of 6 years of post-trial follow-up. The lipid research clinics investigators. Arch. Intern. Med.

[274] The lipid research clinics coronary primary prevention trial results. I. (1984). Reduction in incidence of coronary heart disease. JAMA.

[275] The Lipid Research Clinics Program. (1983). Pre-entry characteristics of participants in the lipid research clinics’ coronary primary prevention trial. J. Chronic Dis..

[276] He L (2014). Lack of effect of colesevelam HCL on the single-dose pharmacokinetics of aspirin, atenolol, enalapril, phenytoin, rosiglitazone, and sitagliptin. Diabetes Res. Clin. Pract..

[277] Blom DJ (2017). Long-term efficacy and safety of the microsomal triglyceride transfer protein inhibitor lomitapide in patients with homozygous familial hypercholesterolemia. Circulation.

[278] Alonso R, Cuevas A, Mata P (2019). Lomitapide: a review of its clinical use, efficacy, and tolerability. Core Evid..

[279] d’Erasmo L (2021). Efficacy and safety of lomitapide in homozygous familial hypercholesterolemia: the pan-european retrospective observational study. Eur. J. Prev. Cardiol..

[280] Cuchel M (2013). Efficacy and safety of a microsomal triglyceride transfer protein inhibitor in patients with homozygous familial hypercholesterolaemia: a single-arm, open-label, phase 3 study. Lancet.

[281] Ahmad Z (2019). Inhibition of angiopoietin-like protein 3 with a monoclonal antibody reduces triglycerides in hypertriglyceridemia. Circulation.

[282] In LiverTox: clinical and research information on drug-induced liver injury (2012). 31643176

[283] Hurt-Camejo E (2020). ANGPTL3, PCSK9, and statin therapy drive remarkable reductions in hyperlipidemia and atherosclerosis in a mouse model. J. Lipid Res..

[284] Pouwer MG (2020). Alirocumab, evinacumab, and atorvastatin triple therapy regresses plaque lesions and improves lesion composition in mice. J. Lipid Res..

[285] Ling P (2021). Targeting angiopoietin-like 3 in atherosclerosis: from bench to bedside. Diabetes Obes. Metab..

[286] Hua H (2018). PPARα-independent action against metabolic syndrome development by fibrates is mediated by inhibition of STAT3 signalling. J. Pharm. Pharm..

[287] Chapman MJ, Redfern JS, McGovern ME, Giral P (2010). Niacin and fibrates in atherogenic dyslipidemia: pharmacotherapy to reduce cardiovascular risk. Pharm. Ther..

[288] Bruckert E, Labreuche J, Deplanque D, Touboul PJ, Amarenco P (2011). Fibrates effect on cardiovascular risk is greater in patients with high triglyceride levels or atherogenic dyslipidemia profile: a systematic review and meta-analysis. J. Cardiovasc. Pharm..

[289] Lee M, Saver JL, Towfighi A, Chow J, Ovbiagele B (2011). Efficacy of fibrates for cardiovascular risk reduction in persons with atherogenic dyslipidemia: a meta-analysis. Atherosclerosis.

[290] Fruchart JC (2017). Pemafibrate (K-877), a novel selective peroxisome proliferator-activated receptor alpha modulator for management of atherogenic dyslipidaemia. Cardiovasc. Diabetol..

[291] Davidson MH, Armani A, McKenney JM, Jacobson TA (2007). Safety considerations with fibrate therapy. Am. J. Cardiol..

[292] van Wijk DF (2014). Nonpharmacological lipoprotein apheresis reduces arterial inflammation in familial hypercholesterolemia. J. Am. Coll. Cardiol..

[293] Thompson G, Parhofer KG (2019). Current role of lipoprotein apheresis. Curr. Atheroscler. Rep..

[294] Hegele RA, Tsimikas S (2019). Lipid-lowering agents. Circ. Res..

[295] Zvintzou E (2017). Pleiotropic effects of apolipoprotein C3 on HDL functionality and adipose tissue metabolic activity. J. Lipid Res..

[296] Yao Z (2012). Human apolipoprotein C-III - a new intrahepatic protein factor promoting assembly and secretion of very low density lipoproteins. Cardiovasc. Hematol. Disord. Drug Targets.

[297] Gordts PL (2016). Apoc-III inhibits clearance of triglyceride-rich lipoproteins through LDL family receptors. J. Clin. Investig..

[298] Lee SJ, Campos H, Moye LA, Sacks FM (2003). LDL containing apolipoprotein CIII is an independent risk factor for coronary events in diabetic patients. Arterioscler. Thromb. Vasc. Biol..

[299] Sacks FM (2000). VLDL, apolipoproteins B, CIII, and E, and risk of recurrent coronary events in the cholesterol and recurrent events (CARE) trial. Circulation.

[300] Wyler von Ballmoos MC, Haring B, Sacks FM (2015). The risk of cardiovascular events with increased apolipoprotein CIII: a systematic review and meta-analysis. J. Clin. Lipido.

[301] Scheffer PG (2008). Increased plasma apolipoprotein C-III concentration independently predicts cardiovascular mortality: the Hoorn Study. Clin. Chem..

[302] Pollin TI (2008). A null mutation in human APOC3 confers a favorable plasma lipid profile and apparent cardioprotection. Science.

[303] Jorgensen AB, Frikke-Schmidt R, Nordestgaard BG, Tybjaerg-Hansen A (2014). Loss-of-function mutations in APOC3 and risk of ischemic vascular disease. N. Engl. J. Med.

[304] Gaudet D (2015). Antisense inhibition of apolipoprotein C-III in patients with hypertriglyceridemia. N. Engl. J. Med..

[305] Alexander VJ (2019). N-acetyl galactosamine-conjugated antisense drug to APOC3 mRNA, triglycerides and atherogenic lipoprotein levels. Eur. Heart J..

[306] Authors/Task Force M, Guidelines ESCCFP, Societies ESCNC (2019). 2019 ESC/EAS guidelines for the management of dyslipidaemias: lipid modification to reduce cardiovascular risk. Atherosclerosis.

[307] Nordestgaard BG, Langsted A (2016). Lipoprotein (a) as a cause of cardiovascular disease: Insights from epidemiology, genetics, and biology. J. Lipid Res..

[308] Kamstrup PR (2021). Lipoprotein(a) and cardiovascular disease. Clin. Chem..

[309] Thanassoulis G (2016). Lipoprotein (a) in calcific aortic valve disease: from genomics to novel drug target for aortic stenosis. J. Lipid Res..

[310] Emerging Risk Factors C (2009). Lipoprotein(a) concentration and the risk of coronary heart disease, stroke, and nonvascular mortality. JAMA.

[311] Kamstrup PR, Benn M, Tybjaerg-Hansen A, Nordestgaard BG (2008). Extreme lipoprotein(a) levels and risk of myocardial infarction in the general population: the copenhagen city heart study. Circulation.

[312] Clarke R (2009). Genetic variants associated with Lp(a) lipoprotein level and coronary disease. N. Engl. J. Med..

[313] Boffa MB, Marcovina SM, Koschinsky ML (2004). Lipoprotein(a) as a risk factor for atherosclerosis and thrombosis: mechanistic insights from animal models. Clin. Biochem..

[314] Deb A, Caplice NM (2004). Lipoprotein(a): new insights into mechanisms of atherogenesis and thrombosis. Clin. Cardiol..

[315] Langsted A, Nordestgaard BG, Kamstrup PR (2019). Elevated lipoprotein(a) and risk of ischemic stroke. J. Am. Coll. Cardiol..

[316] Nielsen LB (1999). Atherogenecity of lipoprotein(a) and oxidized low density lipoprotein: insight from in vivo studies of arterial wall influx, degradation and efflux. Atherosclerosis.

[317] Thompson GR (2022). The scientific basis and future of lipoprotein apheresis. Ther. Apher. Dial..

[318] Parish S (2018). Impact of apolipoprotein(a) isoform size on lipoprotein(a) lowering in the HPS2-THRIVE Study. Circ. Genom. Precis. Med.

[319] Santos RD (2015). Long-term efficacy and safety of mipomersen in patients with familial hypercholesterolaemia: 2-year interim results of an open-label extension. Eur. Heart J..

[320] O’Donoghue ML (2019). Lipoprotein(a), PCSK9 inhibition, and cardiovascular risk. Circulation.

[321] Tsimikas S (2020). Lipoprotein(a) reduction in persons with cardiovascular disease. N. Engl. J. Med..

[322] Tsimikas S, Moriarty PM, Stroes ES (2021). Emerging RNA therapeutics to lower blood levels of Lp(a): JACC focus seminar 2/4. J. Am. Coll. Cardiol..

[323] Yvan-Charvet L (2007). Combined deficiency of ABCA1 and ABCG1 promotes foam cell accumulation and accelerates atherosclerosis in mice. J. Clin. Investig..

[324] Wang X (2007). Macrophage ABCA1 and ABCG1, but not SR-BI, promote macrophage reverse cholesterol transport in vivo. J. Clin. Investig..

[325] Naik SU (2006). Pharmacological activation of liver X receptors promotes reverse cholesterol transport in vivo. Circulation.

[326] Joseph SB, Castrillo A, Laffitte BA, Mangelsdorf DJ, Tontonoz P (2003). Reciprocal regulation of inflammation and lipid metabolism by liver X receptors. Nat. Med..

[327] Chen J (2012). Liver X receptor activation attenuates plaque formation and improves vasomotor function of the aortic artery in atherosclerotic ApoE^-/-^ mice. Inflamm. Res..

[328] Joseph SB (2002). Synthetic LXR ligand inhibits the development of atherosclerosis in mice. Proc. Natl Acad. Sci. USA.

[329] Repa JJ (2000). Regulation of mouse sterol regulatory element-binding protein-1c gene (SREBP-1c) by oxysterol receptors, LXRα and LXRβ. Genes Dev..

[330] Yasuda T (2010). Tissue-specific liver X receptor activation promotes macrophage reverse cholesterol transport in vivo. Arterioscler. Thromb. Vasc. Biol..

[331] Li N (2017). Identification of a novel liver X receptor agonist that regulates the expression of key cholesterol homeostasis genes with distinct pharmacological characteristics. Mol. Pharm..

[332] Chen Y (2015). Inhibition of ERK1/2 and activation of LXR synergistically reduce atherosclerotic lesions in ApoE-deficient mice. Arterioscler. Thromb. Vasc. Biol..

[333] Ma C (2018). Functional interplay between liver X receptor and AMP-activated protein kinase α inhibits atherosclerosis in apolipoprotein E-deficient mice—a new anti-atherogenic strategy. Br. J. Pharm..

[334] Ma C (2021). Targeting macrophage liver X receptors by hydrogel-encapsulated T0901317 reduces atherosclerosis without effect on hepatic lipogenesis. Br. J. Pharm..

[335] Villard EF (2013). Endogenous CETP activity as a predictor of cardiovascular risk: determination of the optimal range. Atherosclerosis.

[336] Zhang J (2017). Deficiency of cholesteryl ester transfer protein protects against atherosclerosis in rabbits. Arterioscler. Thromb. Vasc. Biol..

[337] Barter PJ (2007). Effects of torcetrapib in patients at high risk for coronary events. N. Engl. J. Med..

[338] Schwartz GG (2012). Effects of dalcetrapib in patients with a recent acute coronary syndrome. N. Engl. J. Med.

[339] Lincoff AM (2017). Evacetrapib and cardiovascular outcomes in high-risk vascular disease. N. Engl. J. Med..

[340] Group HTRC (2017). Effects of anacetrapib in patients with atherosclerotic vascular disease. N. Engl. J. Med..

[341] Hovingh GK (2015). Cholesterol ester transfer protein inhibition by TA-8995 in patients with mild dyslipidaemia (TULIP): A randomised, double-blind, placebo-controlled phase 2 trial. Lancet.

[342] Mehta JL, Chen J, Hermonat PL, Romeo F, Novelli G (2006). Lectin-like, oxidized low-density lipoprotein receptor-1 (LOX-1): a critical player in the development of atherosclerosis and related disorders. Cardiovasc. Res..

[343] Hinagata J (2006). Oxidized LDL receptor LOX-1 is involved in neointimal hyperplasia after balloon arterial injury in a rat model. Cardiovasc. Res..

[344] Hu C (2008). LOX-1 deletion decreases collagen accumulation in atherosclerotic plaque in low-density lipoprotein receptor knockout mice fed a high-cholesterol diet. Cardiovasc. Res..

[345] Ishigaki Y (2008). Impact of plasma oxidized low-density lipoprotein removal on atherosclerosis. Circulation.

[346] Xu S (2012). Tanshinone II-A inhibits oxidized LDL-induced LOX-1 expression in macrophages by reducing intracellular superoxide radical generation and NF-κB activation. Transl. Res.

[347] Kang BY (2010). Curcumin reduces angiotensin II-mediated cardiomyocyte growth via LOX-1 inhibition. J. Cardiovasc. Pharm..

[348] Ou HC (2009). Ginkgo biloba extract attenuates oxLDL-induced oxidative functional damages in endothelial cells. J. Appl. Physiol..

[349] Francone OL (2009). The hydrophobic tunnel present in LOX-1 is essential for oxidized ldl recognition and binding. J. Lipid Res..

[350] Thakkar S (2015). Structure-based design targeted at LOX-1, a receptor for oxidized low-density lipoprotein. Sci. Rep..

[351] Pothineni NVK (2017). LOX-1 in atherosclerosis and myocardial ischemia: biology, genetics, and modulation. J. Am. Coll. Cardiol..

[352] Linton MF, Tao H, Linton EF, Yancey PG (2017). SR-BI: a multifunctional receptor in cholesterol homeostasis and atherosclerosis. Trends Endocrinol. Metab..

[353] Zanoni P (2016). Rare variant in scavenger receptor BI raises HDL cholesterol and increases risk of coronary heart disease. Science.

[354] Zhang X, Fernandez-Hernando C (2019). The Janus-faced role of SR-BI in atherosclerosis. Nat. Metab..

[355] Vitali C, Cuchel M (2021). Controversial role of lecithin: cholesterol acyltransferase in the development of atherosclerosis: new insights from an LCAT activator. Arterioscler. Thromb. Vasc. Biol..

[356] Kunnen S, Van Eck M (2012). Lecithin: cholesterol acyltransferase: old friend or foe in atherosclerosis?. J. Lipid Res..

[357] Oldoni F (2018). Complete and partial lecithin: cholesterol acyltransferase deficiency is differentially associated with atherosclerosis. Circulation.

[358] Shamburek RD (2016). Safety and tolerability of ACP-501, a recombinant human lecithin: cholesterol acyltransferase, in a phase 1 single-dose escalation study. Circ. Res..

[359] Bonaca MP (2021). Recombinant human lecithin-cholesterol acyltransferase in patients with atherosclerosis: phase 2a primary results and phase 2b design. Eur. Heart J. Cardiovasc. Pharmacother..

[360] Freeman LA (2017). Lecithin: cholesterol acyltransferase activation by sulfhydryl-reactive small molecules: role of cysteine-31. J. Pharm. Exp. Ther..

[361] Chen Z (2012). Small molecule activation of lecithin cholesterol acyltransferase modulates lipoprotein metabolism in mice and hamsters. Metabolism.

[362] Manthei KA (2018). Molecular basis for activation of lecithin: cholesterol acyltransferase by a compound that increases HDL cholesterol. Elife.

[363] Sasaki M (2021). Novel LCAT (Lecithin: cholesterol acyltransferase) activator DS-8190a prevents the progression of plaque accumulation in atherosclerosis models. Arterioscler. Thromb. Vasc. Biol..

[364] Krol J, Loedige I, Filipowicz W (2010). The widespread regulation of microrna biogenesis, function and decay. Nat. Rev. Genet..

[365] Canfran-Duque A, Lin CS, Goedeke L, Suarez Y, Fernandez-Hernando C (2016). Micro-RNAs and high-density lipoprotein metabolism. Arterioscler. Thromb. Vasc. Biol..

[366] Goedeke L, Wagschal A, Fernandez-Hernando C, Naar A (2016). M. miRNA regulation of LDL-cholesterol metabolism. Biochim. Biophys. Acta.

[367] Goedeke L, Aranda JF, Fernandez-Hernando C (2014). microRNA regulation of lipoprotein metabolism. Curr. Opin. Lipido.

[368] Najafi-Shoushtari SH (2010). MicroRNA-33 and the SREBP host genes cooperate to control cholesterol homeostasis. Science.

[369] Rayner KJ (2010). MiR-33 contributes to the regulation of cholesterol homeostasis. Science.

[370] Marquart TJ, Allen RM, Ory DS, Baldan A (2010). MiR-33 links SREBP-2 induction to repression of sterol transporters. Proc. Natl Acad. Sci. USA.

[371] Horie T (2010). MicroRNA-33 encoded by an intron of sterol regulatory element-binding protein 2 (SREBP2) regulates HDL in vivo. Proc. Natl Acad. Sci. USA.

[372] Rottiers V (2013). Pharmacological inhibition of a microRNA family in nonhuman primates by a seed-targeting 8-mer antimiR. Sci. Transl. Med..

[373] Price NL (2018). Genetic ablation of miR-33 increases food intake, enhances adipose tissue expansion, and promotes obesity and insulin resistance. Cell Rep..

[374] Price NL (2017). Genetic dissection of the impact of miR-33a and miR-33b during the progression of atherosclerosis. Cell Rep..

[375] Horie T (2012). MicroRNA-33 deficiency reduces the progression of atherosclerotic plaque in ApoE^-/-^ mice. J. Am. Heart Assoc..

[376] Willeit P (2017). Circulating microRNA-122 is associated with the risk of new-onset metabolic syndrome and type 2 diabetes. Diabetes.

[377] Esau C (2006). MiR-122 regulation of lipid metabolism revealed by in vivo antisense targeting. Cell Metab..

[378] Elmen J (2008). LNA-mediated microRNA silencing in non-human primates. Nature.

[379] Castoldi M (2011). The liver-specific microRNA miR-122 controls systemic iron homeostasis in mice. J. Clin. Investig..

[380] Hsu SH (2012). Essential metabolic, anti-inflammatory, and anti-tumorigenic functions of miR-122 in liver. J. Clin. Investig..

[381] Chalasani N (2012). The diagnosis and management of non-alcoholic fatty liver disease: practice Guideline by the American Gastroenterological Association, American Association for the Study of Liver Diseases, and American College of Gastroenterology. Gastroenterology.

[382] Puri P (2007). A lipidomic analysis of nonalcoholic fatty liver disease. Hepatology.

[383] Ioannou GN (2016). The role of cholesterol in the pathogenesis of nash. Trends Endocrinol. Metab..

[384] Musso G, Gambino R, Cassader M (2013). Cholesterol metabolism and the pathogenesis of non-alcoholic steatohepatitis. Prog. Lipid Res..

[385] Yasutake K (2009). Nutritional investigation of non-obese patients with non-alcoholic fatty liver disease: the significance of dietary cholesterol. Scand. J. Gastroenterol..

[386] Musso G (2003). Dietary habits and their relations to insulin resistance and postprandial lipemia in nonalcoholic steatohepatitis. Hepatology.

[387] Savard C (2013). Synergistic interaction of dietary cholesterol and dietary fat in inducing experimental steatohepatitis. Hepatology.

[388] Wouters K (2010). Intrahepatic cholesterol influences progression, inhibition and reversal of non-alcoholic steatohepatitis in hyperlipidemic mice. FEBS Lett..

[389] Li H, Yu XH, Ou X, Ouyang XP, Tang CK (2021). Hepatic cholesterol transport and its role in non-alcoholic fatty liver disease and atherosclerosis. Prog. Lipid Res..

[390] Caballero F (2009). Enhanced free cholesterol, SREBP-2 and StAR expression in human NASH. J. Hepatol..

[391] Simonen P (2011). Cholesterol synthesis is increased and absorption decreased in non-alcoholic fatty liver disease independent of obesity. J. Hepatol..

[392] Ioannou GN (2017). Cholesterol crystallization within hepatocyte lipid droplets and its role in murine NASH. J. Lipid Res..

[393] Wallace MC, Friedman SL, Mann DA (2015). Emerging and disease-specific mechanisms of hepatic stellate cell activation. Semin. Liver Dis..

[394] Khajehahmadi Z (2019). Downregulation of hedgehog ligands in human simple steatosis may protect against nonalcoholic steatohepatitis: Is TAZ a crucial regulator?. IUBMB Life.

[395] Mooring M (2020). Hepatocyte stress increases expression of yes-associated protein and transcriptional coactivator with PDZ-binding motif in hepatocytes to promote parenchymal inflammation and fibrosis. Hepatology.

[396] Wang X (2016). Hepatocyte TAZ/WWTR1 promotes inflammation and fibrosis in nonalcoholic steatohepatitis. Cell Metab..

[397] Wang X (2020). Cholesterol stabilizes TAZ in hepatocytes to promote experimental non-alcoholic steatohepatitis. Cell Metab..

[398] Musso G, Cassader M, Gambino R (2011). Cholesterol-lowering therapy for the treatment of nonalcoholic fatty liver disease: an update. Curr. Opin. Lipido.

[399] Tziomalos K (2014). Lipid-lowering agents in the management of nonalcoholic fatty liver disease. World J. Hepatol..

[400] Pastori D (2015). The efficacy and safety of statins for the treatment of non-alcoholic fatty liver disease. Dig. Liver Dis..

[401] Musso G (2014). Ezetimibe in the balance: can cholesterol-lowering drugs alone be an effective therapy for NAFLD?. Diabetologia.

[402] Takeshita Y (2014). The effects of ezetimibe on non-alcoholic fatty liver disease and glucose metabolism: a randomised controlled trial. Diabetologia.

[403] Loomba R (2015). Ezetimibe for the treatment of nonalcoholic steatohepatitis: Aassessment by novel magnetic resonance imaging and magnetic resonance elastography in a randomized trial (MOZART trial). Hepatology.

[404] Francque SM (2021). A randomized, controlled trial of the pan-PPAR agonist lanifibranor in NASH. N. Engl. J. Med..

[405] Yang X (2021). Hepatocyte SH3RF2 deficiency is a key aggravator for NAFLD. Hepatology.

[406] Rottiers V, Naar AM (2012). MicroRNAs in metabolism and metabolic disorders. Nat. Rev. Mol. Cell Biol..

[407] Haslam DW, James WP (2005). Obesity. Lancet.

[408] Kim, M., Lee, C. & Park, J. Extracellular matrix remodeling facilitates obesity-associated cancer progression. *Trends Cell Biol*. 10.1016/j.tcb.2022.02.008 (2022). 10.1016/j.tcb.2022.02.008 35307288

[409] Salem V (2022). Prevalence, risk factors, and interventions for obesity in Saudi Arabia: a systematic review. Obes. Rev..

[410] Chung S (2014). Dietary cholesterol promotes adipocyte hypertrophy and adipose tissue inflammation in visceral, but not in subcutaneous, fat in monkeys. Arterioscler. Thromb. Vasc. Biol..

[411] Lamri A, Pigeyre M, Garver WS, Meyre D (2018). The extending spectrum of NPC1-related human disorders: from Niemann-Pick C1 disease to obesity. Endocr. Rev..

[412] The adipocyte and obesity: cellular and molecular mechanisms. (1983). Abstracts from an international conference, university of toronto, 28 and 29 june, 1982. Int. J. Obes..

[413] Gregor MF, Hotamisligil GS (2011). Inflammatory mechanisms in obesity. Annu. Rev. Immunol..

[414] Villarroya F, Cereijo R, Gavalda-Navarro A, Villarroya J, Giralt M (2018). Inflammation of brown/beige adipose tissues in obesity and metabolic disease. J. Intern. Med.

[415] Curley S, Gall J, Byrne R, Yvan-Charvet L, McGillicuddy FC (2021). Metabolic inflammation in obesity-at the crossroads between fatty acid and cholesterol metabolism. Mol. Nutr. Food Res..

[416] Grover GJ (2003). Selective thyroid hormone receptor-beta activation: a strategy for reduction of weight, cholesterol, and lipoprotein (a) with reduced cardiovascular liability. Proc. Natl Acad. Sci. USA.

[417] Barbe P (2001). Triiodothyronine-mediated up-regulation of UCP2 and UCP3 mRNA expression in human skeletal muscle without coordinated induction of mitochondrial respiratory chain genes. FASEB J..

[418] Finan B (2016). Chemical hybridization of glucagon and thyroid hormone optimizes therapeutic impact for metabolic disease. Cell.

[419] Berbee JF (2015). Brown fat activation reduces hypercholesterolaemia and protects from atherosclerosis development. Nat. Commun..

[420] Baxter JD, Webb P, Grover G, Scanlan TS (2004). Selective activation of thyroid hormone signaling pathways by GC-1: a new approach to controlling cholesterol and body weight. Trends Endocrinol. Metab..

[421] Meslier V (2020). Mediterranean diet intervention in overweight and obese subjects lowers plasma cholesterol and causes changes in the gut microbiome and metabolome independently of energy intake. Gut.

[422] Ho M (2013). Impact of dietary and exercise interventions on weight change and metabolic outcomes in obese children and adolescents: a systematic review and meta-analysis of randomized trials. JAMA Pediatr..

[423] Marcus F, Gontero B, Harrsch PB, Rittenhouse J (1986). Amino acid sequence homology among fructose-1,6-bisphosphatases. Biochem. Biophys. Res. Commun..

[424] Goldberg RB (1981). Lipid disorders in diabetes. Diabetes Care.

[425] Wilson FP, Williams OT (1907). Note on the occurrence and constitution on lipoid material in diabetic blood. Biochem. J..

[426] Henderson AM (1946). A case of diabetes mellitus with hyperlipaemia and hypercholesterolaemia. Med. J. Aust..

[427] Brunham LR (2007). Beta-cell ABCA1 influences insulin secretion, glucose homeostasis and response to thiazolidinedione treatment. Nat. Med..

[428] Hao M, Head WS, Gunawardana SC, Hasty AH, Piston DW (2007). Direct effect of cholesterol on insulin secretion: a novel mechanism for pancreatic beta-cell dysfunction. Diabetes.

[429] Vergeer M (2010). Carriers of loss-of-function mutations in ABCA1 display pancreatic beta-cell dysfunction. Diabetes Care.

[430] Xu Y, Toomre DK, Bogan JS, Hao M (2017). Excess cholesterol inhibits glucose-stimulated fusion pore dynamics in insulin exocytosis. J. Cell Mol. Med..

[431] Carrasco-Pozo C (2016). The deleterious effect of cholesterol and protection by quercetin on mitochondrial bioenergetics of pancreatic beta-cells, glycemic control and inflammation: in vitro and in vivo studies. Redox Biol..

[432] Zhao YF (2010). Cholesterol induces mitochondrial dysfunction and apoptosis in mouse pancreatic beta-cell line MIN6 cells. Endocrine.

[433] Chen ZY (2014). Atorvastatin helps preserve pancreatic β cell function in obese C57BL/6 J mice and the effect is related to increased pancreas proliferation and amelioration of endoplasmic-reticulum stress. Lipids Health Dis..

[434] Cochran BJ (2016). Impact of perturbed pancreatic β-cell cholesterol homeostasis on adipose tissue and skeletal muscle metabolism. Diabetes.

[435] Siebel AL (2013). Effects of high-density lipoprotein elevation with cholesteryl ester transfer protein inhibition on insulin secretion. Circ. Res..

[436] Cederberg H (2015). Increased risk of diabetes with statin treatment is associated with impaired insulin sensitivity and insulin secretion: a 6 year follow-up study of the metsim cohort. Diabetologia.

[437] Preiss D (2011). Risk of incident diabetes with intensive-dose compared with moderate-dose statin therapy: a meta-analysis. JAMA.

[438] Sattar N (2010). Statins and risk of incident diabetes: a collaborative meta-analysis of randomised statin trials. Lancet.

[439] Shen L (2020). Atorvastatin targets the islet mevalonate pathway to dysregulate mtor signaling and reduce β-cell functional mass. Diabetes.

[440] Zhong Y (2012). Effect of ezetimibe on insulin secretion in db/db diabetic mice. Exp. Diabetes Res..

[441] Wijesekara N (2012). MiR-33a modulates ABCA1 expression, cholesterol accumulation, and insulin secretion in pancreatic islets. Diabetes.

[442] Kang MH (2013). Regulation of ABCA1 protein expression and function in hepatic and pancreatic islet cells by miR-145. Arterioscler. Thromb. Vasc. Biol..

[443] Zhang J, Liu Q (2015). Cholesterol metabolism and homeostasis in the brain. Protein Cell.

[444] Li D, Zhang J, Liu Q (2022). Brain cell type-specific cholesterol metabolism and implications for learning and memory. Trends Neurosci..

[445] Leduc V, Jasmin-Belanger S, Poirier J (2010). ApoE and cholesterol homeostasis in Alzheimer’s disease. Trends Mol. Med..

[446] Wolozin B (2004). Cholesterol and the biology of Alzheimer’s disease. Neuron.

[447] Shobab LA, Hsiung GY, Feldman HH (2005). Cholesterol in Alzheimer’s disease. Lancet Neurol..

[448] Zambon D (2010). Higher incidence of mild cognitive impairment in familial hypercholesterolemia. Am. J. Med..

[449] Silva T, Teixeira J, Remiao F, Borges F (2013). Alzheimer’s disease, cholesterol, and statins: the junctions of important metabolic pathways. Angew. Chem. Int. Ed. Engl..

[450] Xiong H (2008). Cholesterol retention in alzheimer’s brain is responsible for high beta- and gamma-secretase activities and abeta production. Neurobiol. Dis..

[451] Cutler RG (2004). Involvement of oxidative stress-induced abnormalities in ceramide and cholesterol metabolism in brain aging and Alzheimer’s disease. Proc. Natl Acad. Sci. USA.

[452] Sparks DL (1994). Induction of Alzheimer-like beta-amyloid immunoreactivity in the brains of rabbits with dietary cholesterol. Exp. Neurol..

[453] Poirier J, Apolipoprotein E (2003). and cholesterol metabolism in the pathogenesis and treatment of Alzheimer’s disease. Trends Mol. Med..

[454] Gao Y, Tan L, Yu JT, Tan L (2018). Tau in Alzheimer’s disease: mechanisms and therapeutic strategies. Curr. Alzheimer Res..

[455] Knopman DS (2021). Alzheimer disease. Nat. Rev. Dis. Prim..

[456] Villemagne VL (2019). Imaging of tau deposits in adults with Niemann-Pick type C disease: a case-control study. Eur. J. Nucl. Med. Mol. Imaging.

[457] van der Kant R (2019). Cholesterol metabolism is a druggable axis that independently regulates tau and amyloid-β in iPSC-derived Alzheimer’s disease neurons. Cell Stem Cell.

[458] Fan QW, Yu W, Senda T, Yanagisawa K, Michikawa M (2001). Cholesterol-dependent modulation of tau phosphorylation in cultured neurons. J. Neurochem..

[459] Kalia LV, Lang AE (2015). Parkinson’s disease. Lancet.

[460] Sohmiya M (2004). Redox status of plasma coenzyme Q10 indicates elevated systemic oxidative stress in Parkinson’s disease. J. Neurol. Sci..

[461] Teunissen CE (2003). Combination of serum markers related to several mechanisms in Alzheimer’s disease. Neurobiol. Aging.

[462] de Lau LM, Koudstaal PJ, Hofman A, Breteler MM (2006). Serum cholesterol levels and the risk of Parkinson’s disease. Am. J. Epidemiol..

[463] Rozani V (2018). Higher serum cholesterol and decreased Parkinson’s disease risk: a statin-free cohort study. Mov. Disord..

[464] Hu G, Antikainen R, Jousilahti P, Kivipelto M, Tuomilehto J (2008). Total cholesterol and the risk of Parkinson disease. Neurology.

[465] de Oliveira J (2013). Diphenyl diselenide prevents cortico-cerebral mitochondrial dysfunction and oxidative stress induced by hypercholesterolemia in LDL receptor knockout mice. Neurochem. Res..

[466] Thirumangalakudi L (2008). High cholesterol-induced neuroinflammation and amyloid precursor protein processing correlate with loss of working memory in mice. J. Neurochem..

[467] Bar-On P (2008). Statins reduce neuronal alpha-synuclein aggregation in in vitro models of Parkinson’s disease. J. Neurochem..

[468] Kruger R (1999). Increased susceptibility to sporadic Parkinson’s disease by a certain combined alpha-synuclein/apolipoprotein E genotype. Ann. Neurol..

[469] Fantini J, Carlus D, Yahi N (2011). The fusogenic tilted peptide (67-78) of α-synuclein is a cholesterol binding domain. Biochim. Biophys. Acta.

[470] Hsiao JT, Halliday GM, Kim WS (2017). α-synuclein regulates neuronal cholesterol efflux. Molecules.

[471] Walker FO (2007). Huntington’s disease. Lancet.

[472] Kacher R, Mounier C, Caboche J, Betuing S (2022). Altered cholesterol homeostasis in Huntington’s disease. Front. Aging Neurosci..

[473] Hooghwinkel GJ, Borri PF, Bruyn GW (1966). Biochemical studies in Huntington’s chorea. II. Composition of blood lipids. Acta Neurol. Scand..

[474] Sipione S (2002). Early transcriptional profiles in Huntingtin-inducible striatal cells by microarray analyses. Hum. Mol. Genet..

[475] Leoni V (2011). Whole body cholesterol metabolism is impaired in Huntington’s disease. Neurosci. Lett..

[476] Valenza M (2007). Cholesterol biosynthesis pathway is disturbed in YAC128 mice and is modulated by huntingtin mutation. Hum. Mol. Genet..

[477] Valenza M (2007). Progressive dysfunction of the cholesterol biosynthesis pathway in the R6/2 mouse model of huntington’s disease. Neurobiol. Dis..

[478] Valenza M (2010). Cholesterol defect is marked across multiple rodent models of Huntington’s disease and is manifest in astrocytes. J. Neurosci..

[479] Valenza M (2005). Dysfunction of the cholesterol biosynthetic pathway in Huntington’s disease. J. Neurosci..

[480] Suzuki S (2007). Brain-derived neurotrophic factor regulates cholesterol metabolism for synapse development. J. Neurosci..

[481] Gauthier LR (2004). Huntingtin controls neurotrophic support and survival of neurons by enhancing BDNF vesicular transport along microtubules. Cell.

[482] Karasinska JM, Hayden MR (2011). Cholesterol metabolism in Huntington disease. Nat. Rev. Neurol..

[483] del Toro D (2010). Altered cholesterol homeostasis contributes to enhanced excitotoxicity in Huntington’s disease. J. Neurochem..

[484] Sierra S (2011). Statins as neuroprotectants: a comparative in vitro study of lipophilicity, blood-brain-barrier penetration, lowering of brain cholesterol, and decrease of neuron cell death. J. Alzheimers Dis..

[485] van der Most PJ, Dolga AM, Nijholt IM, Luiten PG, Eisel UL (2009). Statins: mechanisms of neuroprotection. Prog. Neurobiol..

[486] Kumar A, Sharma N, Mishra J, Kalonia H (2013). Synergistical neuroprotection of rofecoxib and statins against malonic acid induced Huntington’s disease like symptoms and related cognitive dysfunction in rats. Eur. J. Pharm..

[487] Yan J, Sun J, Huang L, Fu Q, Du G (2014). Simvastatin prevents neuroinflammation by inhibiting N-methyl-D-aspartic acid receptor 1 in 6-hydroxydopamine-treated PC12 cells. J. Neurosci. Res..

[488] Jick H, Zornberg GL, Jick SS, Seshadri S, Drachman DA (2000). Statins and the risk of dementia. Lancet.

[489] Friedhoff LT, Cullen EI, Geoghagen NS, Buxbaum JD (2001). Treatment with controlled-release lovastatin decreases serum concentrations of human beta-amyloid (A beta) peptide. Int. J. Neuropsychopharmacol..

[490] Li G (2007). Statin therapy is associated with reduced neuropathologic changes of Alzheimer disease. Neurology.

[491] Rea TD (2005). Statin use and the risk of incident dementia: the cardiovascular health study. Arch. Neurol..

[492] Wolozin B (2007). Simvastatin is associated with a reduced incidence of dementia and Parkinson’s disease. BMC Med.

[493] Bykov K, Yoshida K, Weisskopf MG, Gagne JJ (2017). Confounding of the association between statins and parkinson disease: systematic review and meta-analysis. Pharmacoepidemiol. Drug Saf..

[494] Lee YC (2013). Discontinuation of statin therapy associates with parkinson disease: a population-based study. Neurology.

[495] Liu G (2017). Statins may facilitate Parkinson’s disease: insight gained from a large, national claims database. Mov. Disord..

[496] Rozani V (2017). Statin adherence and the risk of Parkinson’s disease: a population-based cohort study. PLoS One.

[497] Refolo LM (2001). A cholesterol-lowering drug reduces β-amyloid pathology in a transgenic mouse model of Alzheimer’s disease. Neurobiol. Dis..

[498] Whitney KD (2002). Regulation of cholesterol homeostasis by the liver X receptors in the central nervous system. Mol. Endocrinol..

[499] Chen JY (2016). Partial amelioration of peripheral and central symptoms of Huntington’s disease via modulation of lipid metabolism. J. Huntingt. Dis..

[500] Xu P (2013). LXR agonists: new potential therapeutic drug for neurodegenerative diseases. Mol. Neurobiol..

[501] Cermenati G (2013). Liver X receptors, nervous system, and lipid metabolism. J. Endocrinol. Investig..

[502] Yao J (2012). Neuroprotection by cyclodextrin in cell and mouse models of Alzheimer disease. J. Exp. Med..

[503] Bar-On P (2006). Effects of the cholesterol-lowering compound methyl-beta-cyclodextrin in models of alpha-synucleinopathy. J. Neurochem..

[504] Ikonen E (2008). Cellular cholesterol trafficking and compartmentalization. Nat. Rev. Mol. Cell Biol..

[505] Mooberry LK, Sabnis NA, Panchoo M, Nagarajan B, Lacko AG (2016). Targeting the SR-BI receptor as a gateway for cancer therapy and imaging. Front. Pharm..

[506] Ahn J (2009). Prediagnostic total and high-density lipoprotein cholesterol and risk of cancer. Cancer Epidemiol. Biomark. Prev..

[507] Riscal R, Skuli N, Simon MC (2019). Even cancer cells watch their cholesterol!. Mol. Cell.

[508] Tosi MR, Tugnoli V (2005). Cholesteryl esters in malignancy. Clin. Chim. Acta.

[509] Xu D (2020). The gluconeogenic enzyme PCK1 phosphorylates INSIG1/2 for lipogenesis. Nature.

[510] Furuta E (2008). Fatty acid synthase gene is up-regulated by hypoxia via activation of Akt and sterol regulatory element binding protein-1. Cancer Res.

[511] Huang B, Song BL, Xu C (2020). Cholesterol metabolism in cancer: mechanisms and therapeutic opportunities. Nat. Metab..

[512] Pontini L, Marinozzi M (2021). Shedding light on the roles of liver X receptors in cancer by using chemical probes. Br. J. Pharm..

[513] Xu H, Zhou S, Tang Q, Xia H, Bi F (2020). Cholesterol metabolism: new functions and therapeutic approaches in cancer. Biochim. Biophys. Acta Rev. Cancer.

[514] Han M (2020). Therapeutic implications of altered cholesterol homeostasis mediated by loss of CYP46A1 in human glioblastoma. EMBO Mol. Med..

[515] Oni TE (2020). SOAT1 promotes mevalonate pathway dependency in pancreatic cancer. J. Exp. Med..

[516] Zhu, Y. et al. P53 deficiency affects cholesterol esterification to exacerbate hepatocarcinogenesis. *Hepatology*. 10.1002/hep.32518 (2022). 10.1002/hep.32518 PMC11186660 35398929

[517] Ma X (2019). Cholesterol induces CD8^+^ T cell exhaustion in the tumor microenvironment. Cell Metab..

[518] Goossens P (2019). Membrane cholesterol efflux drives tumor-associated macrophage reprogramming and tumor progression. Cell Metab..

[519] Garwood ER (2010). Fluvastatin reduces proliferation and increases apoptosis in women with high grade breast cancer. Breast Cancer Res. Treat..

[520] Clendening JW (2010). Exploiting the mevalonate pathway to distinguish statin-sensitive multiple myeloma. Blood.

[521] Longo J (2019). An actionable sterol-regulated feedback loop modulates statin sensitivity in prostate cancer. Mol. Metab..

[522] Seckl MJ (2017). Multicenter, phase III, randomized, double-blind, placebo-controlled trial of pravastatin added to first-line standard chemotherapy in small-cell lung cancer (LUNGSTAR). J. Clin. Oncol..

[523] Lim SH (2015). A randomised, double-blind, placebo-controlled multi-centre phase III trial of XELIRI/FOLFIRI plus simvastatin for patients with metastatic colorectal cancer. Br. J. Cancer.

[524] Jouve JL (2019). Pravastatin combination with sorafenib does not improve survival in advanced hepatocellular carcinoma. J. Hepatol..

[525] Kim ST (2014). Simvastatin plus capecitabine-cisplatin versus placebo plus capecitabine-cisplatin in patients with previously untreated advanced gastric cancer: a double-blind randomised phase 3 study. Eur. J. Cancer.

[526] Luo Y, Liu L, Li X, Shi Y (2020). Avasimibe inhibits the proliferation, migration and invasion of glioma cells by suppressing linc00339. Biomed. Pharmacother..

[527] Bemlih S, Poirier MD, El Andaloussi A (2010). Acyl-coenzyme A: cholesterol acyltransferase inhibitor Avasimibe affect survival and proliferation of glioma tumor cell lines. Cancer Biol. Ther..

[528] Jiang Y (2019). Proteomics identifies new therapeutic targets of early-stage hepatocellular carcinoma. Nature.

[529] Pommier AJ (2010). Liver X receptor activation downregulates AKT survival signaling in lipid rafts and induces apoptosis of prostate cancer cells. Oncogene.

[530] Solomon KR (2009). Ezetimibe is an inhibitor of tumor angiogenesis. Am. J. Pathol..

[531] Miura K (2019). Ezetimibe suppresses development of liver tumors by inhibiting angiogenesis in mice fed a high-fat diet. Cancer Sci..

[532] Yuan J (2021). Potentiating CD8^+^ T cell antitumor activity by inhibiting PCSK9 to promote LDLR-mediated tcr recycling and signaling. Protein Cell.

[533] Song S, Guo Y, Yang Y, Fu D (2022). Advances in pathogenesis and therapeutic strategies for osteoporosis. Pharm. Ther..

[534] Paschalis EP (2017). Vitamin D and calcium supplementation for three years in postmenopausal osteoporosis significantly alters bone mineral and organic matrix quality. Bone.

[535] Yamaguchi T (2002). Plasma lipids and osteoporosis in postmenopausal women. Endocr. J..

[536] Yerges-Armstrong LM (2013). Decreased bone mineral density in subjects carrying familial defective apolipoprotein B-100. J. Clin. Endocrinol. Metab..

[537] Jeong TD (2014). Relationship between serum total cholesterol level and serum biochemical bone turnover markers in healthy pre- and postmenopausal women. Biomed. Res. Int..

[538] Tarakida A (2011). Hypercholesterolemia accelerates bone loss in postmenopausal women. Climacteric.

[539] Parhami F (2001). Atherogenic high-fat diet reduces bone mineralization in mice. J. Bone Min. Res..

[540] You L (2011). High cholesterol diet increases osteoporosis risk via inhibiting bone formation in rats. Acta Pharm. Sin..

[541] Pelton K (2012). Hypercholesterolemia promotes an osteoporotic phenotype. Am. J. Pathol..

[542] Cutillas-Marco E, Prosper AF, Grant WB, Morales-Suarez-Varela MM (2013). Vitamin D status and hypercholesterolemia in spanish general population. Dermatoendocrinology.

[543] Luegmayr E (2004). Osteoclast formation, survival and morphology are highly dependent on exogenous cholesterol/lipoproteins. Cell Death Differ..

[544] Sanbe T (2007). Oral administration of vitamin C prevents alveolar bone resorption induced by high dietary cholesterol in rats. J. Periodontol..

[545] Lin SM, Wang JH, Liang CC, Huang HK (2018). Statin use is associated with decreased osteoporosis and fracture risks in stroke patients. J. Clin. Endocrinol. Metab..

[546] An T (2017). Efficacy of statins for osteoporosis: a systematic review and meta-analysis. Osteoporos. Int..

[547] Leutner M (2019). Diagnosis of osteoporosis in statin-treated patients is dose-dependent. Ann. Rheum. Dis..

[548] Oryan A, Kamali A, Moshiri A (2015). Potential mechanisms and applications of statins on osteogenesis: current modalities, conflicts and future directions. J. Control Release.

[549] Brown DA, London E (2000). Structure and function of sphingolipid- and cholesterol-rich membrane rafts. J. Biol. Chem..

[550] Ripa I, Andreu S, Lopez-Guerrero JA, Bello-Morales R (2021). Membrane rafts: portals for viral entry. Front. Microbiol..

[551] Schroeder C (2010). Cholesterol-binding viral proteins in virus entry and morphogenesis. Subcell. Biochem..

[552] Osuna-Ramos JF, Reyes-Ruiz JM, Del Angel RM (2018). The role of host cholesterol during flavivirus infection. Front. Cell Infect. Microbiol..

[553] Palmer M (2004). Cholesterol and the activity of bacterial toxins. FEMS Microbiol. Lett..

[554] Louie AY (2022). Dietary cholesterol causes inflammatory imbalance and exacerbates morbidity in mice infected with influenza A virus. J. Immunol..

[555] Wang, Y. et al. Pseudorabies virus inhibits expression of liver X receptors to assist viral infection. *Viruses* **14** (2022). 10.3390/v14030514 PMC8954865 35336921

[556] Palacios-Rapalo SN (2021). Cholesterol-rich lipid rafts as platforms for SARS-CoV-2 entry. Front Immunol..

[557] Radenkovic D, Chawla S, Pirro M, Sahebkar A, Banach M (2020). Cholesterol in relation to COVID-19: Should we care about it?. J. Clin. Med..

[558] Sanders DW (2021). SARS-CoV-2 requires cholesterol for viral entry and pathological syncytia formation. Elife.

[559] Lu Y, Liu DX, Tam JP (2008). Lipid rafts are involved in SARS-CoV entry into Vero E6 cells. Biochem. Biophys. Res. Commun..

[560] Li GM, Li YG, Yamate M, Li SM, Ikuta K (2007). Lipid rafts play an important role in the early stage of severe acute respiratory syndrome-coronavirus life cycle. Microbes Infect..

[561] Meher G, Bhattacharjya S, Chakraborty H (2019). Membrane cholesterol modulates oligomeric status and peptide-membrane interaction of severe acute respiratory syndrome coronavirus fusion peptide. J. Phys. Chem. B.

[562] Fasolato S (2020). PCSK9 levels are raised in chronic HCV patients with hepatocellular carcinoma. J. Clin. Med..

[563] Andriulli A (2008). Meta-analysis: the outcome of anti-viral therapy in HCV genotype 2 and genotype 3 infected patients with chronic hepatitis. Aliment. Pharm. Ther..

[564] Hyrina A (2017). Treatment-induced viral cure of hepatitis C virus-infected patients involves a dynamic interplay among three important molecular players in lipid homeostasis: circulating microRNA (miR)-24, miR-223, and proprotein convertase subtilisin/kexin type 9. EBioMedicine.

[565] Li Z, Liu Q (2018). Proprotein convertase subtilisin/kexin type 9 inhibits hepatitis C virus replication through interacting with NS5A. J. Gen. Virol..

[566] Li Z, Liu Q (2018). Hepatitis C virus regulates proprotein convertase subtilisin/kexin type 9 promoter activity. Biochem. Biophys. Res. Commun..

[567] Gan ES (2020). Dengue virus induces PCSK9 expression to alter antiviral responses and disease outcomes. J. Clin. Investig..

[568] Diczfalusy U (2013). On the formation and possible biological role of 25-hydroxycholesterol. Biochimie.

[569] Liu SY (2013). Interferon-inducible cholesterol-25-hydroxylase broadly inhibits viral entry by production of 25-hydroxycholesterol. Immunity.

[570] Li C (2017). 25-hydroxycholesterol protects host against zika virus infection and its associated microcephaly in a mouse model. Immunity.

[571] Xiang Y (2015). Identification of cholesterol 25-hydroxylase as a novel host restriction factor and a part of the primary innate immune responses against hepatitis C virus infection. J. Virol..

[572] Zu S (2020). 25-hydroxycholesterol is a potent SARS-CoV-2 inhibitor. Cell Res.

[573] Zang R (2020). Cholesterol 25-hydroxylase suppresses SARS-CoV-2 replication by blocking membrane fusion. Proc. Natl Acad. Sci. USA.

[574] Wang Q (2014). Identification of interferon-gamma as a new molecular target of liver X receptor. Biochem. J..

[575] Liu Y (2018). Activation of liver X receptor plays a central role in antiviral actions of 25-hydroxycholesterol. J. Lipid Res..

[576] Liu Y (2018). 25-hydroxycholesterol activates the expression of cholesterol 25-hydroxylase in an LXR-dependent mechanism. J. Lipid Res..

[577] Li B (2020). Prevalence and impact of cardiovascular metabolic diseases on COVID-19 in china. Clin. Res. Cardiol..

[578] Wu Z, McGoogan JM (2020). Characteristics of and important lessons from the coronavirus disease 2019 (COVID-19) outbreak in China: Summary of a report of 72314 cases from the Chinese center for disease control and prevention. JAMA.

[579] Gorabi AM (2020). Antiviral effects of statins. Prog. Lipid Res..

[580] Zhang XJ (2020). In-hospital use of statins is associated with a reduced risk of mortality among individuals with COVID-19. Cell Metab..

[581] Proto MC (2021). Lipid homeostasis and mevalonate pathway in COVID-19: basic concepts and potential therapeutic targets. Prog. Lipid Res..

[582] Torres-Pena JD (2021). Prior treatment with statins is associated with improved outcomes of patients with COVID-19: Data from the semi-COVID-19 registry. Drugs.

[583] Huang W, Xiao J, Ji J, Chen L (2021). Association of lipid-lowering drugs with COVID-19 outcomes from a mendelian randomization study. Elife.

[584] Subir R, Jagat JM, Kalyan KG (2020). Pros and cons for use of statins in people with coronavirus disease-19 (COVID-19). Diabetes Metab. Syndr..

[585] Reiner Z (2020). Statins and the COVID-19 main protease: in silico evidence on direct interaction. Arch. Med. Sci..

[586] Boccara F (2020). Evolocumab in HIV-infected patients with dyslipidemia: primary results of the randomized, double-blind BEIJERINCK study. J. Am. Coll. Cardiol..

[587] Vuorio A, Watts GF, Kovanen PT (2020). Familial hypercholesterolaemia and COVID-19: triggering of increased sustained cardiovascular risk. J. Intern. Med..

[588] Vuorio A, Kovanen PT (2020). Prevention of endothelial dysfunction and thrombotic events in COVID-19 patients with familial hypercholesterolemia. J. Clin. Lipido.

